# Re-assessing the diversity of negative strand RNA viruses in insects

**DOI:** 10.1371/journal.ppat.1008224

**Published:** 2019-12-12

**Authors:** Simon Käfer, Sofia Paraskevopoulou, Florian Zirkel, Nicolas Wieseke, Alexander Donath, Malte Petersen, Terry C. Jones, Shanlin Liu, Xin Zhou, Martin Middendorf, Sandra Junglen, Bernhard Misof, Christian Drosten

**Affiliations:** 1 Institute of Virology, Charité-Universitätsmedizin Berlin, Corporate Member of Free University, Humboldt-University and Berlin Institute of Health, Berlin, Germany; 2 Center for Molecular Biodiversity Research, Zoological Research Museum Alexander Koenig, Bonn, Germany; 3 Institute of Virology, University of Bonn Medical Centre, Bonn, Germany; 4 Swarm Intelligence and Complex Systems Group, Department of Computer Science, Leipzig University, Leipzig, Germany; 5 Senckenberg Biodiversity and Climate Research Centre, Senckenberg Gesellschaft für Naturforschung, Frankfurt am Main, Germany; 6 Center for Pathogen Evolution, Department of Zoology, University of Cambridge, Cambridge, United Kingdom; 7 BGI-Shenzhen, China Beishan Industrial Zone, Shenzhen, Guangdong Province, China; 8 College of Food Science and Nutritional Engineering, China Agricultural University, Beijing, China; 9 Beijing Advanced Innovation Center for Food Nutrition and Human Health, China Agricultural University, Beijing, China; 10 German Center for Infection Research (DZIF), associated partner site Charité, Berlin, Germany; University of Cambridge, UNITED KINGDOM

## Abstract

The spectrum of viruses in insects is important for subjects as diverse as public health, veterinary medicine, food production, and biodiversity conservation. The traditional interest in vector-borne diseases of humans and livestock has drawn the attention of virus studies to hematophagous insect species. However, these represent only a tiny fraction of the broad diversity of Hexapoda, the most speciose group of animals. Here, we systematically probed the diversity of negative strand RNA viruses in the largest and most representative collection of insect transcriptomes from samples representing all 34 extant orders of Hexapoda and 3 orders of Entognatha, as well as outgroups, altogether representing 1243 species. Based on profile hidden Markov models we detected 488 viral RNA-directed RNA polymerase (RdRp) sequences with similarity to negative strand RNA viruses. These were identified in members of 324 arthropod species. Selection for length, quality, and uniqueness left 234 sequences for analyses, showing similarity to genomes of viruses classified in *Bunyavirales* (n = 86), *Articulavirales* (n = 54), and several orders within *Haploviricotina* (n = 94). Coding-complete genomes or nearly-complete subgenomic assemblies were obtained in 61 cases. Based on phylogenetic topology and the availability of coding-complete genomes we estimate that at least 20 novel viral genera in seven families need to be defined, only two of them monospecific. Seven additional viral clades emerge when adding sequences from the present study to formerly monospecific lineages, potentially requiring up to seven additional genera. One long sequence may indicate a novel family. For segmented viruses, cophylogenies between genome segments were generally improved by the inclusion of viruses from the present study, suggesting that *in silico* misassembly of segmented genomes is rare or absent. Contrary to previous assessments, significant virus-host codivergence was identified in major phylogenetic lineages based on two different approaches of codivergence analysis in a hypotheses testing framework. In spite of these additions to the known spectrum of viruses in insects, we caution that basing taxonomic decisions on genome information alone is challenging due to technical uncertainties, such as the inability to prove integrity of complete genome assemblies of segmented viruses.

## Author summary

The diversity of insect viruses is relevant to medical, environmental, and food sciences. Our knowledge of insect viruses is highly biased because medical research has focused on mosquitoes and a few other blood-feeding species. While insects are the most diversified group of animals on the planet, the great majority of all insect species remain completely unexamined for viruses. Here we searched the most comprehensive and most evenly composed collection of insects for negative strand RNA viruses based on full transcriptomes. In 1243 insect species of all orders, we found 488 independent viral sequences encoding an RNA-directed RNA polymerase, a signature gene for RNA viruses. These data add considerably to our knowledge on viral diversity, and reveal that viruses have coevolved with insect hosts. However, our results also provide a reminder of the pitfalls associated with virus discovery and taxonomic classification in the age of metagenomics.

## Introduction

Negative strand RNA viruses contain major groups of pathogenic viruses that cause rabies, hemorrhagic fevers, respiratory infections, measles, as well as a large range of important diseases and economically important conditions in livestock and plants [1–4]. Our current knowledge of negative strand RNA viruses is biased by the interest in medical disciplines and provides an incomplete image when it comes to more fundamental questions in viral evolution, such as the contribution of codivergence in the formation of major viral genetic lineages. These questions can only be addressed by systematic studies of larger taxonomic units of viral hosts, corresponding to whole orders or classes of animals, which is complicated by the difficulty to establish representative sample collections. Samples utilized for viral diversity studies are often collected on an opportunistic basis or repurposed from other studies, resulting in imbalance in host species representation, uncertainty in host classification, and uncertain assignment of samples. This is especially true for studies of insects that show an enormous genetic and morphological diversity.

Insects are the most speciose group of animals. Their origin has been dated to the early Ordovician, 479 million years ago, a time that predates the formation of terrestrial ecosystems [5]. Insects engage in symbiotic and parasitic relationships with a multitude of plants and animals, and are a vital component of the diet of animals, potentially facilitating virus transmission. Nevertheless, research on insect viruses has been mainly driven by interest in vector-borne diseases, resulting in virological studies that have focused on blood-feeding species, with rare exceptions [4, 6, 7]. However, blood-feeding insects represent only a minute fraction of the biological diversity of insects. Studies using massively parallel sequencing of collections of invertebrates have yielded an unprecedented diversity of novel RNA viruses [4, 6, 8]. However, the samples used in these studies only covered a limited range of insect species, contained many other groups of invertebrates such as spiders, worms, and molluscs, and were generated by sample pooling. Uncertain knowledge of host associations in these and other studies have caused a tendency to abandon host association as an important auxiliary criterion for taxonomic classification [9].

Here, we systematically probed the diversity of negative strand RNA viruses in the largest and most representative collection of full transcriptome datasets of arthropods. The collection is designed to represent all extant lineages of Hexapoda without representational bias. This transcriptome database was first utilized for a phylogenomic re-assessment of the Hexapoda phylogeny in 2014, based on 103 full transcriptomes [9]. Since that time, the collection has been significantly extended to now cover 1243 full transcriptome datasets. All datasets including their corresponding unassigned contigs and scaffolds were screened for negative strand RNA viruses. The collection represents all orders of Insecta (insects, n = 1178), the orders Collembola (springtails, n = 23), Protura (coneheads, n = 4), and Diplura (n = 14) of Entognatha, as well as 24 outgroup species pertaining to Crustacea (n = 10), Myriapoda (n = 11), and Chelicerata (n = 3).

## Results

We based our search on conserved sequence motifs within the RdRp gene that is present in the genomes of all replicating RNA viruses without a DNA stage except deltaviruses, and is not present in the genome of the eukaryotic or prokaryotic cell. We utilized profile hidden Markov models (pHMMs) to search for candidate viral RdRp motifs within 42,618,061 contigs and scaffolds which were 66 to 20,314 amino acids long. pHMMs were trained on template amino acid alignments covering the core conserved RdRp regions of representative viruses assigned to the families *Rhabdoviridae*, *Paramyxoviridae*, *Filoviridae*, *Nyamiviridae*, and *Orthomyxoviridae*, as well as the genera *Orthonairovirus*, *Mammarenavirus*, *Jonvirus*, *Orthohantavirus*, *Orthobunyavirus*, *Tospovirus*, *Herbevirus*, *Phlebovirus*, and *Goukovirus*.

**Fig 1:**
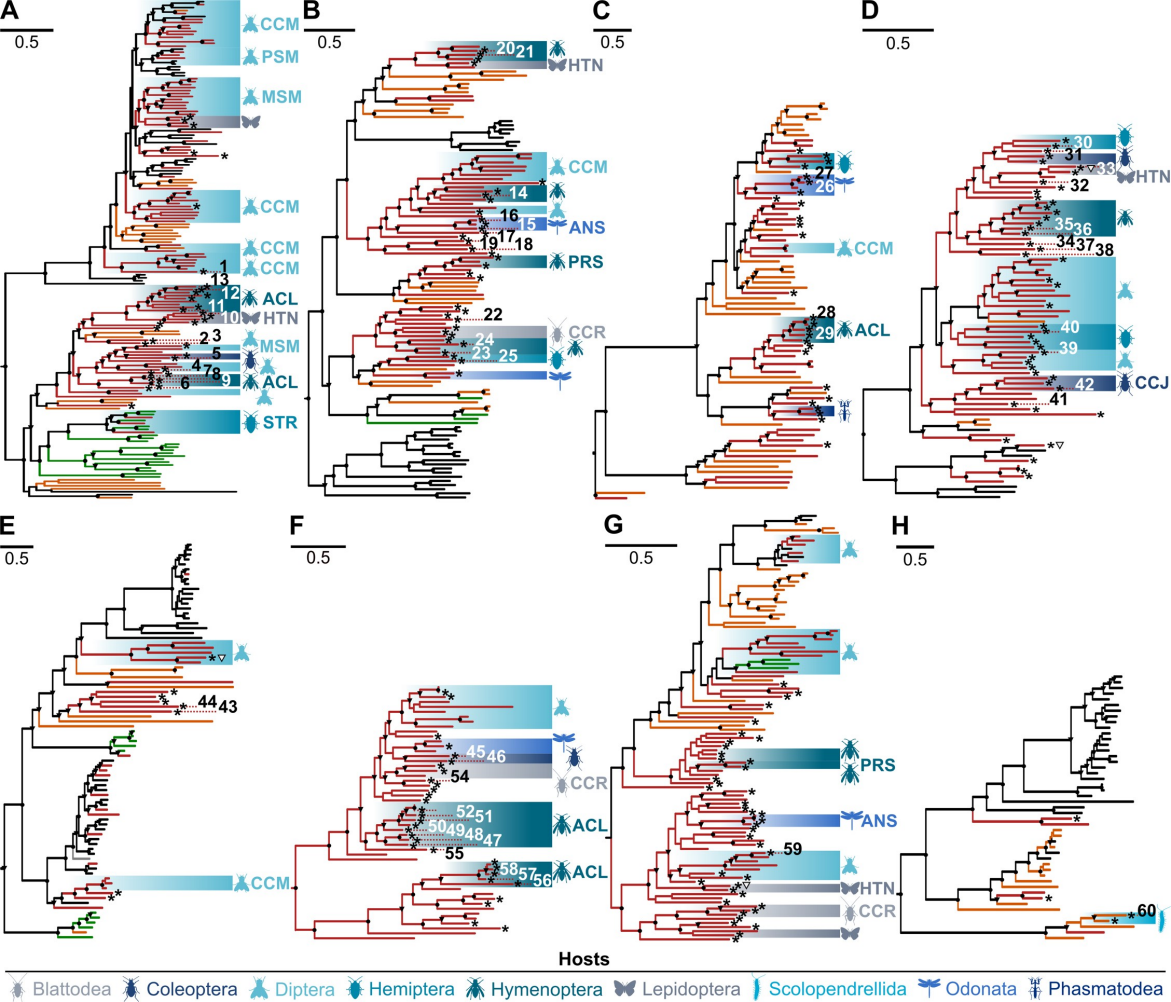
Maximum likelihood phylogenies of viruses found in the present study, viruses defined by ICTV as species, as well as selected unclassified viruses. Novel viruses from the present study are identified by an asterisk. Novel viruses to be considered for taxonomic proposals because of unique phylogenetic position and availability of a coding-complete genome are identified by numbers corresponding to **Table 1**. Trees were inferred in RAxML based on alignments of viral sequences pertaining to: A: *Rhabdoviridae*; B: *Xinmoviridae*, *Nyamiviridae*, *Bornaviridae*, *Artoviridae*, *Lispiviridae*, *Paramyxoviridae*, *Sunviridae*, *Filoviridae*, and *Pneumoniviridae*; C: *Chuviridae*, *Qinviridae*, *and Yueviridae*; D: *Orthomyxoviridae*; E: *Hantaviridae*, *Cruliviridae*, *Peribunyaviridae*, and *Fimoviridae*; F: *Phasmaviridae*; G: *Phenuiviridae*; H: *Arenaviridae*, *Mypoviridae*, *Nairoviridae*, and *Wupedeviridae*. Branch colors show host associations: Black, viruses associated with vertebrates; red, insects; orange, invertebrates other than insects; green, plants. Bootstrap supports on tree nodes are shown by circles (> = 95) and triangles (>70). Designated host infraorders for Blattodea: CCR: Cockroaches; for Coleoptera: CCJ: Cucujiformia; for Diptera: CCM: Culicomorpha, MSM: Muscomorpha, PSM: Psychodomorpha; for Hemiptera: STR: Stenorrhyncha; for Hymenoptera: ACL: Aculeata, PRS: Parasitica; for Lepidoptera: HTN: Heteroneura; for Odonata: ANS: Anisoptera.

According to the results of contig assembly, we initially detected 488 viral RdRp sequences. These were identified in 324 arthropod species belonging to all insect orders and several outgroup taxa. The host associations, exact taxonomic classification, as well as sampling sites of hosts for the viral genomes that appear in the phylogenetic trees in **Fig 1** are summarized in **S1 Table**.

A large proportion of the viral sequences were co-detected with different pHMMs, owing to the nature of the search algorithm which makes the detection of distant homologs possible. This is of particular relevance since the template alignments, as well as the pHMM searches were done before the release of any of the sequences described in [4] and [8]. All data were later re-examined using BLASTp, with the inclusion of the data of [4] and [8], but no additional matches were retrieved. This confirms the sensitivity of the pHMM search approach and demonstrates that the search strategy is not biased by a virus reference library that stems from a fragmentary sample of insect species.

From the obtained contigs, 234 large sequences were selected for further analysis based on length, quality, and dissimilarity toward other sequences in the dataset. These sequences were later found to have highest similarity to members of *Bunyavirales* (n = 86), *Articulavirales* (n = 54), or *Haploviricotina* (n = 94), respectively. For non-segmented viruses, full genome assembly was often successful. For viruses with segmented genomes, assembly focused on the RdRp-encoding segment was later on complemented by BLAST-based searches for other genome segments expected. Thereby, 218 coding sequences from genes that are not encoded on the same segment as the RdRp gene, such as glycoproteins, nucleoproteins, polymerase subunits, and proteins with unknown function were identified. Complete or coding-complete genomes were assembled in 61 cases. Many additional large but incomplete genome assemblies were obtained that contain open reading frames of unknown function and represent unknown genome architectures.

Of note, poly-A purification was applied on all samples due to the intended use for transcriptome analysis [5]. We expected a general loss of sensitivity in detecting viral sequences but did not expect a huge bias against viral genomes as opposed to viral mRNAs. For instance, even during single-cycle infection, more viral genomic RNA than viral mRNA is obtained from poly-A preparation in mononegaviruses, in spite their mRNAs but not genomes contain poly-A tails [10]. In natural infections an even larger excess of genomes over mRNA is expected, because genomic RNA accumulates once packed, while viral mRNA is degraded at a rate that weighs against new synthesis. Also in members of *Bunyavirales* a poly-A–related bias is not expected because neither mRNAs nor genomes of these viruses have poly-A tails.

Novel viruses found in this study are named after their host order, related viral family, and the designation “OKIAV” (for 1KITE insect-associated virus), followed by a number (*e*.*g*., Hemipteran orthomyxo-related virus OKIAV183). All sequences, including genome annotations, host associations, and sampling sites, are available on the Dryad Digital Repository under https://doi.org/10.5061/dryad.87vt6hm. All sequences will be released in GenBank along with the release of transcriptomes from the 1KITE project (BioProject PRJNA183205: 'The 1KITE project: evolution of insects').

### Phylogeny and implications on taxonomy

To enable phylogenetic analysis, contigs were translated and grouped into eight alignments based on preliminary sequence matching and phylogenies. The eight resulting trees as shown in **Fig 1** were generated following intense optimization of alignments by trimming and focusing on conserved domains, with the aim to leave sufficient information in alignments while allowing large taxonomic groups of viruses to be compared. The trees A-H in **Fig 1** cover the *Rhabdoviridae* (**Fig 1A**), all other *Mononegavirales* (**Fig 1B**), *Chu*-, *Qin*-, and *Yueviridae* (**Fig 1C**), *Orthomyxoviridae* (**Fig 1D**), *Hanta*-, *Cruli*-, *Peribunya*-, and *Fimoviridae* (**Fig 1E**), *Phasmaviridae* (**Fig 1G**), *Phenuiviridae* (**Fig 1G**), as well as *Arena*-, *Mypo*-, *Nairo*-, and *Wupedeviridae* (**Fig 1H**). Detailed phylogenies including host associations down to the host species level, as well as viral taxonomy information are shown in **Supporting Information S1–S3** and **S5–S33 Figs**. A detailed description of current taxonomy including the novel virus findings, as well as classification suggestions resulting from the present data are provided in **Supporting Information S1 Text**.

The tree structures in **Fig 1** suggest a remarkable separation between vertebrate and insect viruses, as noted already on the basis of a less inclusive sample of insect diversity [4, 8]. With the exception of the subtree of non-plant-associated rhabdoviruses that still remains star-like and may thus be undersampled, many insect-associated clades now appear well-differentiated with a balanced proportion of intermediate versus terminal branches. In spite of the more inclusive insect sampling contributed by the present study, novel insect viruses remain absent in well-known clades of pathogenic vertebrate viruses, such as the genus *Lyssavirus*, the families *Paramyxoviridae*, *Bornaviridae*, *Filoviridae*, *Hantaviridae*, and *Arenaviridae*. Also, some major groups of pathogenic arboviruses do not show an expansion of host associations following our search. For instance, the phleboviruses and orthobunyaviruses that are known to be mosquito-, sandfly-, midge-, or tick-borne, do not yield any novel insect-associated viruses in our sample in spite of its enormous genetic diversity (note that there are no sandfly, no mosquito, and only two midge species in our sample). This absence is remarkable as also the studies of [4] and [8] did not find any novel phleboviruses in the insects they sampled, while they did find novel phleboviruses in ticks. There may exist an ecologically-driven association of these viruses with blood-feeding insects. The additional association with ticks makes it possible that viruses could be exchanged between insects and ticks based on common bloodmeal sources.

Classification criteria exist only for a minority of viral genera. For instance, amino acid sequence distances of 4% and 5% have been proposed for species demarcation in orthobunyaviruses and phleboviruses, respectively. Our distance-based selection of sequences for inclusion in trees exceeds this distance criterion, making it likely that all novel sequences identified by an asterisk in **Fig 1** could be classified as novel species. While many recently-described viruses still form solitary lineages in trees, a deep topological separation and structured host association emerges after inclusion of data from the present study. Large subclades within viral families are often associated with insect orders or suborders, indicating an important auxiliary criterion for subdivision of these viral families into genera. Structured host associations become particularly obvious in the families *Rhabdoviridae*, *Xinmoviridae*, *Nyamiviridae*, *Artoviridae*, *Lispiviridae*, *Chuviridae*, *Phasmaviridae*, and *Feraviridae* (**Supporting Information S1 Text**). **Table 1** lists those virus clades that we propose to be considered for classification on the genus level based on sequence distance and host associations, while taking into account the completeness of available genome sequences. All in all, the analyzed sequences suggest a potential for classifying at least 27 novel genera based on coding-complete virus genomes, 20 of them without any previously known representative, and identify deep-branching viral lineages that in the future may be classified as three novel families or subfamilies. Host associations are especially informative for subclassification of rhabdo- and xinmoviruses, chuviruses, orthomyxoviruses, and phasmaviruses. Furthermore, we add the first independent description of a full qinvirus genome (Collembolan qin-related virus OKIAV112), detected in the entognath *Anurida maritima* (seashore springtail, class Collembola) (**Fig 1C**). Detailed taxonomical considerations are provided in the **Supporting Information S1 Text.**

**Table 1:** List of phylogenetic groups to be considered for taxonomic proposals.

Superordinate taxon	Putative taxonomic level	Clade annotation	Included tentative species (full genomes)	Source	Remarks	No. in Fig 1
***Rhabdoviridae***	Genus	ARR	Hymenopteran almendra- related virus OKIAV1	This study	Monospecific	1
	Genus	MBAR	Blattodean rhabdo-related virus OKIAV14, Mantodean rhabdo-related virus OKIAV15	This study		2 3
	Genus	DHCR	Dipteran rhabdo-related virus OKIAV19, Coleopteran rhabdo-related virus OKIAV28, Wuhan mosquito virus 9*	This study and Li *et al*.[4]		4 5
	Genus	CAR	Coleopteran rhabdo-related virus OKIAV20	This study	Monospecific	6
	Genus	HAR2	Hymenopteran rhabdo-related virus OKIAV22, -OKIAV23, -OKIAV24	This study		7 8 9
	Genus	LAR	Lepidopteran rhabdo-related virus OKIAV34	This study		10
	Genus	HAR1	Hymenopteran rhabdo-related virus OKIAV38, -OKIAV46, -OKIAV109, Hubei rhabdo-like virus 1	This study and Shi *et al*.[8]		11 12 13
***Xinmoviridae***	Genus	*Anphevirus* lineage I	Xincheng anphevirus** Aedes aegypti anphevirus**, Hymenopteran anphe-related virus OKIAV71	This study, Shi *et al*.[8], and Di Giallonardo *et al*.[11]		14
	Genus	*Anphevirus* lineage II	Odonatan anphe-related virus OKIAV57, -OKIAV59	This study		15 16
	Genus	*Anphevirus* lineage III	Coleopteran anphe-related virus OKIAV54	This study	Subcomplete genome	17
	Genus	*Anphevirus* lineage V	Odonatan anphe-related virus OKIAV90, Mantodean anphe-related virus OKIAV92, *Orthopteran anphevirus*	This study and Shi *et al*. [8]		18 19
***Nyamiviridae***	Genus	*Orinovirus* lineage I	Hymenopteran orino-related virus OKIAV85, -OKIAV87	This study		20 21
***Lispiviridae***	Genus	*Arlivirus* lineage I	Strepsipteran aril-related virus OKIAV104, Hubei arlivirus	This study and Shi *et al*.[8]		22
	Genus	*Arlivirus* lineage III	Hymenopteran arli-related virus OKIAV98, -OKIAV99	This study		23 24
	Genus	*Arlivirus* lineage IV	Hemipteran aril-related virus OKIAV94	This study		25
***Chuviridae***	Genus	OAM	Odonatan chu-related virus OKIAV136, -OKIAV137, *Odonate mivirus*	This study and Shi *et al*.[8]		26 27
	Genus	HyAM	Hymenopteran chu-related virus OKIAV123, -OKIAV124	This study		28 29
***Orthomyxoviridae***	Genus	O1	Hemipteran orthomyxo-related virus OKIAV183, Coleopteran orthomyxo-related virus OKIAV184	This study		30 31
	Genus	O2	Blattodean orthomyxo-related virus OKIAV181, Lepidopteran orthomyxo-related virus OKIAV178	This study		32 33
	Genus	O3	Dermapteran orthomyxo-related virus OKIAV162, Hymenopteran orthomyxo-related virus OKIAV171, Phasmatodean orthomyxo-related virus OKIAV172	This study		34 35 36
	Genus	O4	Siphonapteran orthomyxo-related virus OKIAV157, Coleopteran orthomyxo-related virus OKIAV158	This study	Only 4 segments	37 38
	Genus	O5	Dipteran orthomyxo-related virus OKIAV164	This study		39
	Genus	O6	Hemipteran orthomyxo-related virus OKIAV188	This study		40
	Genus	O7	Dipteran orthomyxo-related virus OKIAV199, Coleopteran orthomyxo-related virus OKIAV200, Hubei orthomyxo-like virus 2	This and Shi *et al*.[8]		41 42
***Bunyavirales***	Family	Novel group	Dipluran hanta-related virus OKIAV217, -OKIAV218	This study	Only 2 segments	43 44
***Bunyavirales***	Family	Not annotated	Collembolan phasma-related virus OKIAV223	This study	Only L-gene	Fig 2
***Phasmaviridae***	Genus	CAP	Coleopteran phasma-related virus OKIAV235, -OKIAV236	This study		45 46
	Genus	HAP	Hymenopteran phasma-related virus OKIAV227, -OKIAV229, -OKIAV230, -OKIAV228, -OKIAV233, -OKIAV234, -OKIAV232, Ganda bee virus	This study and Schoonvaere *et al*.[12]		47 48 49 50 51 52 53
	Genus	MAP1	Coleopteran phasma-related virus OKIAV243	This study		54
	Genus	DAP2	Dipteran phasma-related virus OKIAV226	This study		55
	Genus	HAF	Hymenopteran phasma-related virus OKIAV244, -OKIAV250, -OKIAV252	This study		56 57 58
***Phenuiviridae***	Subfamily	Putative subfamily	Dipteran phenui-related virus OKIAV273, Salarivirus, Shuangao insect virus 3	This study and Li *et al*.[4]		59
***Bunyavirales***	Family	Not annotated	Myriapodan Negavirus OKIAV320, Jiangxia mosquito virus 1	This study and Li *et al*.[4]	Genome status uncertain	60

*Wuhan mosquito virus 9, but none of the other members of the clade, is an endogenous viral element

**Xincheng anphevirus and Aedes aegypti anphevirus, but none of the other members of the clade, are likely to be endogenous viral elements.

ARR: Almendra-related rhabdovirus; DHCR: Diptera-, Hemiptera-, Coleoptera-related rhabdovirus; HAR: Hymenoptera-associated rhabdovirus; LAR: Lepidoptera-associated rhabdovirus; MBAR: Mantodea-/Blattodea-associated rhabdovirus; CAR: Coleoptera-associated rhabdovirus; OAM: Odonata-associated Mivirus; HyAM: Hymenoptera-associated Mivirus; O1-O7: Orthomyxovirus clades 1–7; CAP: Coleoptera-associated phasmaviruses; HAP: Hymenoptera-associated phasmaviruses; MAP1: Multiple host-associated phasmaviruses clade 1; DAP2: Diptera-associated phasmaviruses clade 2; HAF: Hymenoptera-associated feraviruses.

**Fig 2:**
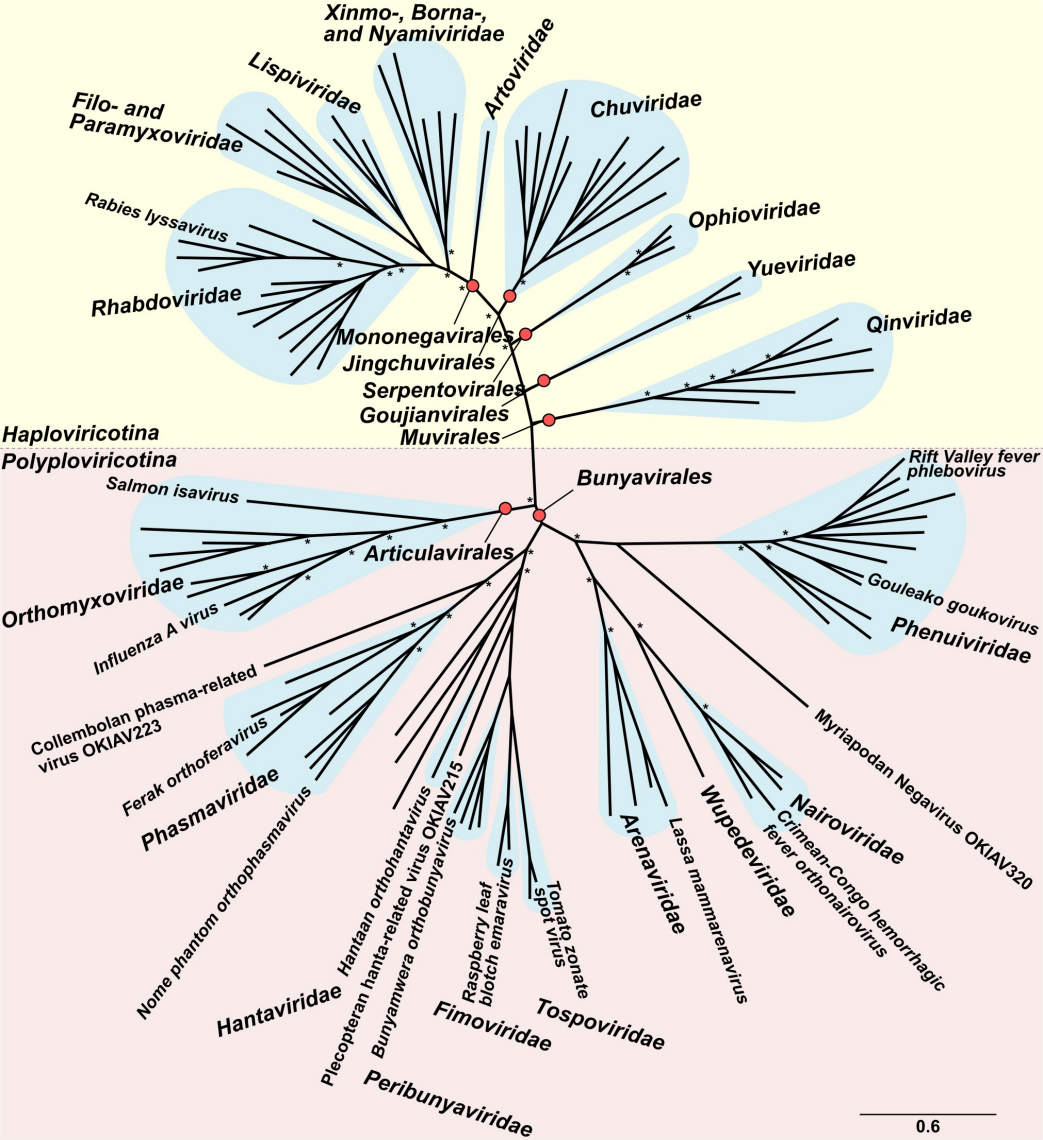
Bayesian phylogeny of negative strand RNA viruses based on MrBayes. Red dots identify virus orders according to current taxonomy. Blue clouds show virus families. Selected viral species are identified for orientation. Asterisks indicate posterior probabilities > 0.9.

The discovery of a large diversity of novel lineages warrants a re-assessment of the overall topology of negative strand viruses. Based on a manually curated alignment, we inferred a tree as shown in **Fig 2** using Bayesian phylogeny. Only few of the topological relationships differ from those in **Fig 1**, which incorporates more alignment information specific for the smaller units of diversity covered therein. For instance, there is weak support for the branching point of rhabdoviruses, as also observed by [13]. As in Shi *et al*. [8] and as implied by current taxonomy, but unlike the results by Wolf *et al*. [13], the members of the order *Articulovirales* branch to the exclusion of all members of *Bunyavirales*, while the deep topology of *Bunyavirales* is well supported. A noteworthy finding is Collembolan phasma-related virus OKIAV223, a large sequence of 8154 nucleotides extending beyond the L-gene ORF, albeit not covering segment termini. It clusters with *Phasmaviridae* and branches from the phasmavirus lineage short after the split from the last common ancestor of *Peribunyaviridae* and *Phasmaviridae* (**Fig 2**). It is therefore the most appropriate outgroup for the peribunyavirus tree (**Fig 1E**), which has been considered for rooting that tree. It is interesting to note that this topology suggests acquisitions of tospo- and emaraviruses by plants from invertebrates, rather than an evolution of peribunyaviruses from plant viruses as suggested by alternative tree topologies. A number of other findings that indicate deeply diverged and novel virus groups are described in **Supporting Information S1 Text**.

### Genome composition

**Fig 3:**
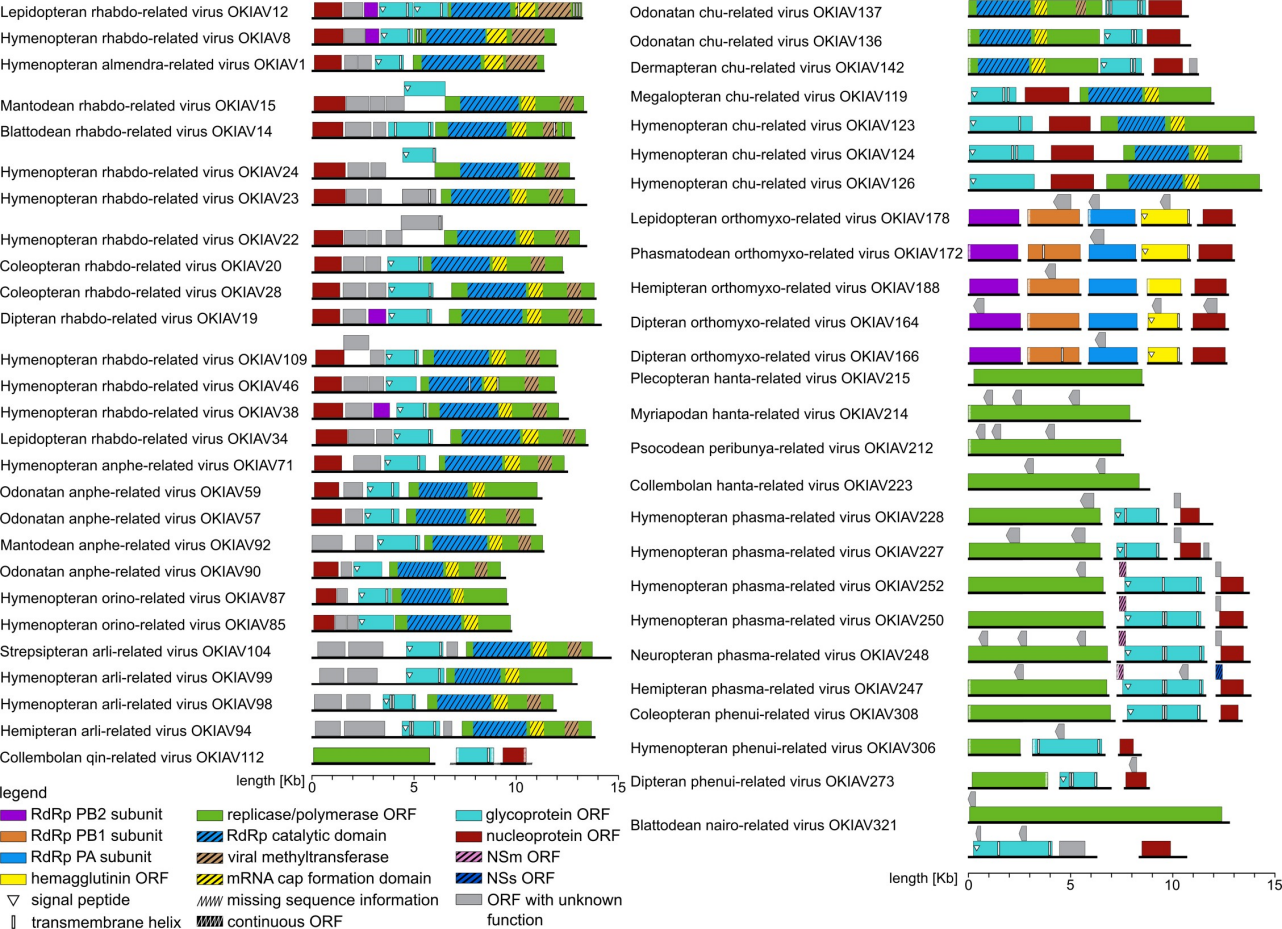
Annotations of full or nearly-full genomes and selected full bunyaviral L-segments found in the present study. Annotation was done using InterProScan [14]. Bunyaviral putative nonstructural genes, as well as other significant subgenomic reading frames were annotated manually.

Tentative structures of complete or nearly-complete genomes are summarized in **Fig 3**. It is noteworthy that genomes of chuviruses were found to appear in linear, circular, and segmented circular forms [4]. **Fig 1C** includes an additional 25 exemplary chuviruses from the present study, including seven with at least one complete segment and one with two complete segments. According to the mapping of raw RNAseq reads, all genomes or genome segments of these viruses are linear. Gene order is L-G-N, or N-G-L (**Fig 3**), confirming the two principal gene orders described in Li *et al*. [4]. Genomes with a missing glycoprotein gene or over-assembled contigs, as described in the same work, are not observed in the present study. To check for circular genome organization, we have re-mapped all raw RNAseq reads to consensus alignments of chuviral sequences joined head to tail. This approach did not find any reads crossing the potential head/tail sequence boundaries, as would be expected in the case of genome segments that are circular. While we do not claim to refute circular genomes in chuviruses, we cannot confirm this genome conformation based on our data (**Supporting Information S34 Fig**) and recommend further experimental validation.

The genome segment termini in members of *Bunyavirales* form complementary panhandle structures [15, 16]. These short sequences are identical within, and similar between genome segments of a given viral genome, and are usually conserved in viruses that belong to one same genus. Because segment termini in most of the members of recently-defined novel viral genera have not been analyzed (including in the present study; refer to **Supporting Information S1 Text**), we determined segment co-segregation as an indicator of grouping congruence of genome elements. Tanglegrams are shown in **Supporting Information S15**, **S19**, **S24**, **S28**, **S29**, and **S33 Figs**. In most major clades there is congruence among segments. Some clades, such as clade C of the orthomyxoviruses (**Supporting Information S15 Fig**) or the clades that define *Shanga-* and *Herbevirus* in the peribunyaviruses, show signs of reassortment in lineage precursors, as topological incongruence is observed for all members of the respective clades. In cases where individual incongruences are seen, such as in Dipteran phasma-related virus OKIAV224, Zorapteran phasma-related virus OKIAV242, or Coleopteran phasma-related virus OKIAV243, we cannot discriminate between *in silico* misassembly of genomes and actual reassortment based on the present data. Confirmation by virus isolation or re-sequencing including genome ends will be necessary.

**Fig 4:**
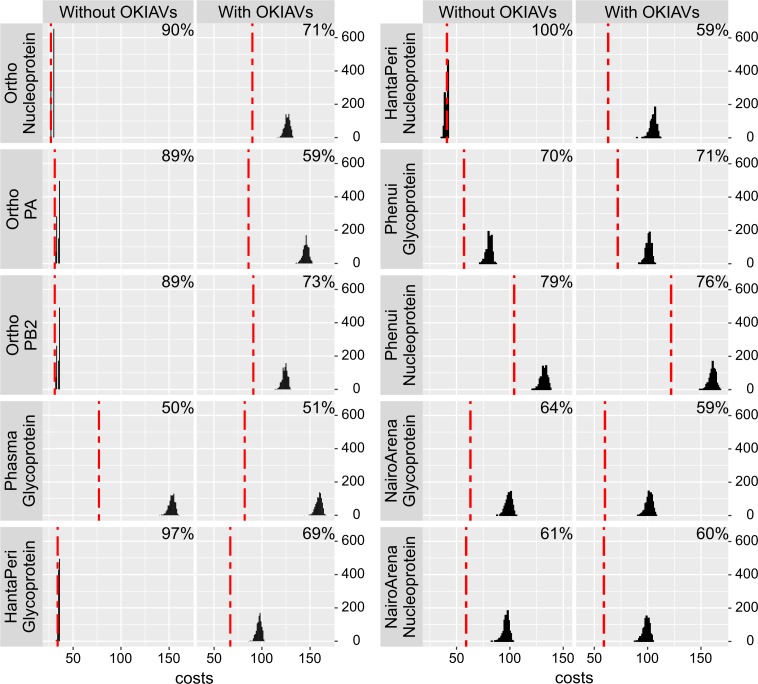
Genome segment cophylogeny costs. Red dotted lines show lowest possible costs for breaches of cophylogeny when using Jane [17] to optimize the tip opposition of RdRp-encoding genome segment trees with the corresponding trees for genome segments indicated in each figure panel name. Black curves show the same costs accrued during each of 1000 different randomizations of the dataset. Percent values in panels show the cophylogeny costs of the real cophylogeny dataset divided by the costs with randomization (medians from 1000 randomizations). Lower percent values indicate better agreement between the RdRp and the respective genome segment cophylogeny.

To obtain an overall impression of segment co-segregation in newly-discovered segmented RNA viruses, we analyzed cophylogenies of RdRp-encoding segments and other segments from the same putative viral genomes using Jane [17]. We compared cophylogeny costs against that of datasets with randomized segment associations. As summarized in **Fig 4**, addition of the present findings rather improved the cophylogeny costs except in cophylogenies between L- and M-segments (RdRp- and glycoprotein-encoding) of phasma- and phenuiviruses where there was no relevant change (**Fig 4**). Also, in some viral trees the addition of the present data reveal segment cosegregation where this was not evident from the genomes of previously described viruses. We thus assume that our segmented genome findings overall do not suffer from *in silico* misassembly or other artifacts. However, the genomes of exemplary strains defining novel genera should be confirmed experimentally.

### Host-virus co-segregation

Recent studies on invertebrate-associated RNA viruses found no evidence of host-virus co-segregation, and proposed frequent cross-host transmission of viruses between insect hosts that co-occupy the same ecological niches [4]. However, these results were based on a limited and spatially restricted sample of insects and other invertebrates. For the present study we have subjected all viral phylogenies to formal cophylogenetic comparisons on the basis of resolved and updated phylogenies of insects as in [5].

**Fig 5:**
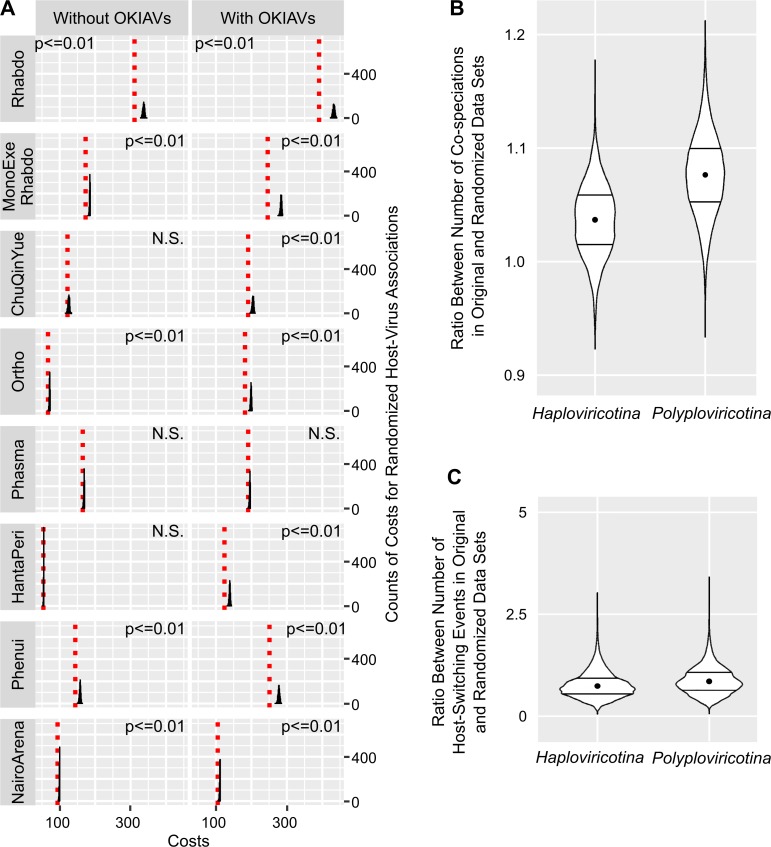
Analysis of host-virus phylogenetic co-segregation. A: Histograms of costs for co-segregation of randomized host associations (1000 iterations) compared to the original host associations (red dotted lines) for all phylogenies, without (left) and with (right) OKIAV sequence inclusion, calculated with Jane [17]. The p-values of all z-tests indicate an increase in costs of over 5% above the original costs. N.S. indicates non-significant cost differences. B: Ratio between number of co-speciations in original and randomized data sets calculated for *Haploviricotina* and *Polyploviricotina* with CoRe-PA [18]. C: Ratio between number of host switching events in original and randomized data sets calculated for *Haploviricotina* and *Polyploviricotina* with CoRe-PA.

The phylogenies shown in **Fig 1** were subjected to tests of breaches of cophylogeny using Jane [17] (refer to **Supporting Information S1 Table** for host associations). To determine the contribution of the novel sequences, separate analyses were done without the OKIAV sequences but incorporating all known and novel viruses as per ICTV taxonomy update end of 2018. The limited knowledge of host associations in most studies restricted the resolution of these analyses to the level of insect orders. Significant virus-host co-segregation was identified in both analyses (with and without OKIAV findings) for the majority of trees (**Fig 5A**). For the trees including *Chuviridae* and *Hanta-/Peribunyaviridae*, significant co-segregation could only be detected when including findings from the present study. Only for the tree summarizing members of *Phasmaviridae*, no host-virus co-segregation could be detected.

Because analysis in Jane does not take branch lengths into account, does not incorporate topological uncertainty, and mainly penalizes breaches of phylogenetic congruence between virus and host tree, an alternative algorithm, CoRe-PA [18], was applied to further examine host-virus co-segregation. This more sophisticated algorithm takes into account the reconstruction of co-speciation and host-switching separately, and also discriminates these from duplication events. To exclude potential bias introduced by the analysis of small phylogenetic trees, we utilized trees that represent the complete tree topologies of *Haploviricotina* and *Polyploviricotina*, including topological uncertainty based on 1000 bootstrap replicates. All possible rootings (i.e., tree versions rooted on every branch) of all replicate trees were modeled in order to exclude the impact of false co-segregation signals among outgroup branches in insect and host trees. Randomization of host associations was performed as previously and used as null hypotheses in separate formal tests of co-segregation and host switching, respectively. As summarized in **Fig 5B** and **5C**, trees of both virus subphyla showed significantly more co-segregation events with real host associations as opposed to randomized host associations. In contrast, tests for host-switching events were not different when analyzing real versus randomized host associations. This suggests that earlier indications of predominance of host-switching were likely to have been caused by uneven sampling, incorrect host attributions, or issues with viral phylogenies.

## Discussion

Our study summarizes curated results from the most comprehensive metagenomic virus screen of a whole class of animals. The host dataset is resolved to all known orders of insects and involves an evenly selected representation of insect families. Because every sample has been genetically classified, the present work provides exact knowledge of host associations for a large range of insect-associated viruses for the first time. This knowledge is essential to study viruses as potential agents of vector-borne diseases in animals or plants, as well as to understand insect viruses in the context of biodiversity conservation and food production [19]. It also offers important criteria to define novel viral species and higher taxa. In this and other regards, our work complements the recent work by Li *et al*. [4] and Shi *et al*. [8] that was mainly focused on the description of viral diversity. For several taxa described in these studies, host associations are specified for the first time. Furthermore, some of the recent taxonomic proposals or re-classifications by ICTV are reconciled by the present data.

While our search has revealed an even greater diversity of positive strand RNA viruses, it has been challenging to curate large numbers of viral sequences while keeping track with taxonomy as it is being changed. The present frequency of taxonomy revisions makes it difficult to design basic analyses, such as phylogenies, so that they incorporate the latest viral taxonomy. The process of taxonomic classification owes to be more conservative and undergo slower cycles of renewal. It should allow time for independent reproduction of sequence findings before these go into taxonomic classification. Independent reproduction is becoming more important as ICTV has recently decided to allow classification on the sole basis of genome sequence data. As our study exemplifies, classification may be uncertain when full genomes cannot reliably be obtained, *e*.*g*., in segmented viruses. Genome segment termini should be tested for complementarity among segments, but are often not covered in sequencing studies [4, 8, 20]. Even in the few cases where genome ends are known and identical, this does not prove that detected segments stem from one viral genome, because the ends of *e*.*g*., bunyaviral segments are identical between viral species and can agree across genera and even families [21]. The study of viral sequences derived from mixtures of insects is particularly difficult, as they may be assembled into chimeric genomes whose fragments may, more likely, stem from different viruses than one virus. The ICTV executive board has stated, with regards to the danger of assembling chimeric genomes, that “These are all caveats that must be addressed experimentally for MG [metagenomic] sequence data to be used for classification purposes” [9]. From a virological view, segmented viruses that are to be classified as species based only on sequence data should have full genome coverage including genome ends, and should stem from individual insects. They should also be examined for phylogenetic congruence between genome segments. Multiple infections should be considered as a source of potential mis-classification. In the present study we were able to infer phylogenies of several segments other than RdRp-encoding segments and perform analyses on co-segregation between the RdRp and the proteins encoded by those segments. While many clades of accepted genera formed monophyletic groups in segment trees, the topology between trees was not always congruent (**Supporting Information S15**, **S19**, **S24**, **S28**, **S29**, and **S33 Figs**). This suggests that reassortment was involved in the formation of major viral taxa. Topological conflicts in individual virus genomes appeared to occur more often in clades consisting of recently discovered viruses ([4, 8, 20], and our study), than in known and functionally-characterized viruses. Even if the present data were not found to disturb topological congruence in phylogenetic analyses of segment association, this emphasizes the general concern regarding hasty classification of viruses identified in metagenomic studies in the absence of experimental evidence.

In many instances, the comprehensiveness of our dataset allows us to estimate the extent of genetic space occupied by taxonomic entities below the family level. Issues such as long-branch attraction and loss of information by reduction of alignments [22] are alleviated by the addition of novel members to formerly solitary lineages, even if their genome sequences are incomplete [23]. The delineation of taxon-specific genetic space is further assisted by reliance on the assumption of long-term co-segregation. In the absence of any other criteria, the knowledge of host associations can provide valuable information to define taxonomic units when newly discovered viruses are added to formerly monogeneric and monospecific families. In light of the obvious under-sampling within viral phylogenies, we regard it as an omission to not use host associations as assistance for virus classification decisions. Host associations allow the intuitive use of the principle of co-segregation that is confirmed for distinct, but related, insect-associated viruses in the present study. Several monophyletic clades in a number of viral families were found to be associated with defined orders of insects, yet all these associations were previously undiscovered [4, 8].

Our data suggest a potential to classify at least 27 novel genera (20 of them without any previously known species), and probably three novel families. Regarding viruses for which complete genomes or live isolates could not be obtained, the knowledge of host associations will orient future efforts to identify or isolate viruses in a more targeted manner. This will be of particular importance in insect groups that have relevance in food production or act as vectors of disease, as well as insect species that change their distribution and abundance due to environmental change.

The utilization of host associations for species classification is an accepted approach in virology, even if, in rare cases, spillovers or dual host associations will have to be taken into account and corrected for [24]. We expect cross-host transmission to be comparatively rare based on our analyses, and challenge the notion of frequent occurrence of cross host-virus transmission in the context of ecological and geographic proximity. The available studies are based on under-sampled viral trees and may suffer from other issues related to host assignment and topological correctness, in particular of viral phylogenies [4]. Tests of co-segregation need to take topological uncertainty into account and should be contrasted to null hypotheses.

We furthermore have concerns regarding the sole reliance on distance-based classification tools in the latest taxonomy proposals for negative strand RNA viruses [25, 26]. The utilized tool, DEmARC [27], analyzes concatenated sets of homologous protein domains that are conserved across the family of interest. It detects local regions of discontinuity in the pairwise distance spectrum within protein primary sequence alignments. Regions of discontinuity define putative limits of taxonomic units below the family level. It has only been evaluated for three families of vertebrate-infecting viruses (*Picornaviridae*, *Coronaviridae*, *Filoviridae*) and not for taxa that contain more than one family. Results for some of the larger virus groups, such as the genera *Enterovirus* versus *Rhinovirus*, have been controversial [28]. If applied to taxonomic units that exceed the family level, changes in genome architecture are common, and it becomes unclear whether protein-encoding genes other than the RdRp are homologous or have instead been acquired by lateral gene transfer [4, 8, 29]. The rate of recombination in newly-discovered viruses is unknown, and in segmented viruses this problem is aggravated by genome segment reassortment. Notably, DEmARC has not been validated at all for segmented viruses. On this basis, the analysis by distance-based tools has to be restricted to the RdRp gene and thus is hardly different from a phylogeny based only on RdRp genes. Other tools, such as the recently-proposed GRAViTy algorithm that incorporates genome composition, should be used and evaluated on the same problem [30].

As demonstrated in the present study and elsewhere [4, 8, 13], there is considerable topological uncertainty in large RNA virus phylogenies that comprise units of genetic diversity corresponding to viral orders. While genetic distance can be estimated in trees, its estimation is based on evolutionary models that cannot accommodate all biological factors of sequence evolution. For instance, viral population sizes, host generation times and infection or coinfection rates are expected to vary considerably between major lineages of viruses carried by hosts as different as mammals, birds, reptiles, fish, insects, spiders, crustaceans, worms, and protists. As these host associations may have been in place since millions of years, effects such as substitution saturation or footprints of recombination are expected to influence the inference of deep phylogenetic relationships. Considering the shortcomings of evolutionary distance as a single classification criterion, additional biological criteria including host associations should be included in taxonomic considerations, if only as a test of plausibility.

Like all other approaches to detect viruses based on sequence information, our work has several additional limitations. For instance, it remains difficult to differentiate endogenous viral elements from replicating viruses. Our approach to extract virus sequence data from insect transcriptomes makes it highly unlikely that integrated, non-transcribed viruses are falsely assigned as viral matches. Earlier studies have relied on abundance of viral transcripts in comparison to total RNA content in RNAseq datasets, without the knowledge of complete transcriptome data. However, none of these approaches can exclude endogenous viral elements with certainty. For this reason, independent confirmation of findings is necessary before taxonomic conclusions are drawn. Our exact knowledge of host associations will enable host genome, as well as virus isolation studies that will ultimately exclude endogenization of viruses in the host germ line and confirm viral replication.

## Materials and methods

### Insect transcriptome data

We screened 1243 insect transcriptomes sequenced within the 1KITE project [5] including species from all recognized extant insect orders and additional arthropod orders. Samples were collected worldwide and RNAseq data were sequenced in an Illumina HiSeq2000 platform. Raw RNAseq data were assembled using SOAPdenovo-Trans-31kmer (version 1.01) [31], and checked for quality and cross contaminations with VecScreen (www.ncbi.nlm.nih.gov/tools/vecscreen/), and UniVec database build 7.0 (www.ncbi.nlm.nih.gov/tools/vecscreen/univec/).

### Viral sequence generation and sorting

Template alignments for building profile hidden Markov models (pHMMs) were created using characterized RdRp amino acid sequences of negative strand RNA virus families. Due to the high sequence divergence of viral genes, even for closely related species, the sequence search was conducted at the amino acid level. Sequences were downloaded from the NCBI database, in October 2014, and aligned with the web-based alignment tool T-coffee in Expresso mode [32]. Transcriptome assemblies were translated in all six ORFs with the *fastatranslate* program within the package EXONERATE (version 2.2.0) [33]. This ORF library was scanned using HMMER version 3 [34] and only sequences with contiguous ORFs were regarded as viral matches. HMMER builds a pHMM from a template alignment and uses it to extract sequences that match the underlying probabilities of the model. This allows for detection of evolutionary distantly related sequences, with the advantage of remote RdRp homolog detection, but also the disadvantage of inflating the results with redundant duplicate sequences. Viral amino acid matches were checked for redundancy with a twofold approach: a) matches from each RdRp-pHMM were aligned to the original template alignments with MAFFT version 7.123, *E-INS-i* [35]. Poorly aligned regions and sequences that were too short and did not overlap with the selected alignment region were removed using trimAl [36] or manually upon inspection of the alignment in Geneious (Geneious v.9.1.8, Biomatters, Auckland, New Zealand, https://www.geneious.com), always complying with the preservation of known RdRp motifs. Trees were inferred with PhyML v.3.2.0 [37], using 1000 bootstrap replicates and Blosum62 amino acid substitution matrix. b) viral hits were compared with BLAST+ v.2.2.28+ [38] against the non-redundant NCBI protein database which had previously been filtered for viral sequences. This twofold approach enabled sorting the viral hits, thus removing any redundancy introduced by the HMM-search among different virus families.

### Inference of alignments and phylogenies

To compose alignments for phylogeny, viral sequence hits (OKIAVs) from the present study were compared to GenBank as of August 2018 using BLASTP with an e-value cutoff of 10^−6^. Sequences over 30% similarity to any OKIAV were selected. All species and genera listed in the ICTV taxonomy table as of end of 2018 were added. During the revision, selected additional species from the ICTV species update released in February 2019 were added. The following literature contributions were additionally consulted and relevant unclassified viruses were added: [39–57].

In total, 234 of the RdRp sequences found in insects in the present study were used for phylogenetic analyses (**Supporting Information S1 Table**). Alignments were calculated anew and refined with trimAl as described above. Model testing in MrBayes identified Blosum62 to be the amino acid substitution matrix compatible with all alignments. Trees were inferred in RAxML-NG version 0.7.0 BETA [58] plotting the transfer bootstrap expectation values [59]. Confirmatory phylogenetic analyses were done in PhyML v.3.2.0 [37] and MrBayes v3.2.6 [60], using the same substitution model and four different substitution rate categories with gamma distribution. For RAxML and PhyML, 1000 bootstrap replicates were computed, and for MrBayes chains were run until fully converged. All trees were plotted and annotated using the R package *ggtree* [61].

### Virus genome organization

All ORFs of the full-length viral hits were annotated after comparing them against our customized viral database as well as with the InterProScan protein domain search tool [14].

### Phylogenetic co-segregation of virus segments

Considering that orthomyxo- and bunyaviruses have segmented genomes, we additionally searched for proteins encoded by other segments (nucleoprotein, glycoprotein, PB2, and PA). For this search, we used the available protein sequences of the respective genera (NCBI) for a BLASTp search within transcriptomes we had detected the RdRp segments in already. Trees shown in **Supporting Information S15**, **S19**, **S24**, **S28**, **S29**, and **S33 Figs** include only those taxa for which additional protein genes were found. The R package *dendextend* [62], was used to create tanglegram figures, that allow examination of topological consistency among the trees. Jane [17] was used to match trees of the RdRp-encoding genome segments to trees of the other segments, based on costs for breaches of cophylogeny (best match = lowest costs). Costs were also determined when segment-segment associations were randomized and these pairs of trees were then subjected to cophylogeny optimization in Jane. To obtain a quantitative measure of topological congruence, the costs associated with the real datasets were divided by the costs with randomization (median from 1000 randomizations). The resulting value is a percentage that expresses the cophylogeny cost relative to a randomly-associated cophylogeny of same tree size and structure (resulting in 0 for perfect cophylogeny and 1 for absence of cophylogeny). These relative costs are expressed as percentages in **Fig 4**. Because adding branches to cophylogenies is expected to increase cophylogeny costs in randomized datasets, this was tested by Wilcoxon´s paired samples test. In all comparisons, the differences were highly significant.

### Analysis of co-segregation of viruses with their hosts

Host-virus associations for each phylogenetic tree were examined to assess concurrent phylogenetic relationships using Jane [17]. As a basis for the host tree we used a modified version of the arthropod phylogeny from Misof *et al*. [5]. Since Jane needs a host for each taxon of the virus tree, unknown or undefined hosts cannot be assessed. Therefore, we added outlier branches of unidentified insect, unidentified arthropod, and non-arthropod hosts, to enable mapping to non-arthropod and pooled insect/arthropod hosts. The co-evolution costs of the original phylogenies were compared to 1000 iterations of randomized host-virus associations. A one-sided z-test (implemented in the R package *BSDA* [63]) was used to test whether the randomized costs are at least 5% higher than the original costs. This threshold was set to ensure that miniscule cost changes do not lead to false interpretations.

CoRe-PA [18] was used to evaluate the co-evolutionary dependencies of the two major virus subphyla of *Haploviricotina* and *Polyploviricotina* with their corresponding insect hosts. Given a co-segregating scenario, CoRe-PA aims to find the most parsimonious reconciliation between host and virus trees by evaluating four co-evolutionary events: co-speciation, sorting, duplication, and host-switching. Each type of event is assigned a certain cost and the co-phylogenetic assessment that minimizes the total cost of events is accepted. Since both *Haploviricotina* and *Polyploviricotina* trees were unrooted, all possible rooted versions were evaluated, meaning that for every edge a rooted tree was created. Insect phylogenies were rooted to the arthropod order Chelicerata. For each of the previously 1000 unrooted RAxML bootstrap trees, 229 and 266 rooted trees were created for *Haploviricotina* and *Polyploviricotina* respectively. A reconciliation for each of these trees to the corresponding insect phylogeny was computed. To determine the strength and significance of host-virus co-evolution, each reconciliation was compared against a randomized association of each co-phylogenetic scenario, keeping the tree topologies unchanged. 100 randomized scenaria were computed by randomly renaming the host tree tips. This preserved the structure of host-virus associations, while avoiding bias introduction from sampling random trees. To estimate the fit of each randomized scenario, reconciliations were computed with the following costs: 0 for sorting and duplication, -1000 for co-speciation, and -0.001 for a host-switching event.

### Completeness of genome segments

The completeness of viral segments was assessed for all segmented-related findings. Segments with size similar to known relative viruses were regarded as at least coding-complete regions, if the segment ORF was terminated by a stop codon within the segment. Bunyaviruses have segments that form panhandles, with conserved, species-specific termini [15, 16]. We examined the genome termini for complementarity, and also evaluated whether the termini of one segment match those of the other segments.

### Cytochrome oxidase subunit 1 (COI) barcode analysis

To investigate the possibility that other organisms were accidentally collected and therefore whether the hosts of the OKIAV viruses might not actually be the intended sampled organisms, a barcode search was conducted based on two databases. First, 2,534,455 cytochrome c oxidase subunit 1 (COI) gene sequences from GenBank sequences were retrieved on October 10, 2019 with the query “txid2759[Organism:exp] AND cytochrome oxidase subunit 1[All fields]”. Second, the German Barcode of Life (GBOL) database, which contains barcode sequences from species recovered in Germany (Animalia: 287,377 barcodes including 261,015 hexapods; Plantae 7,884 barcodes, Fungi 1,038 barcodes). Contigs assembled from the 1243 insect transcriptomes were matched against these databases using BLAST+ (version 2.6.0). The BLAST results were filtered for matches of length at least 500 nucleotides, with a nucleotide identity of at least 98%.

Of the 1243 insect transcriptomes, 34 (2.73%) have at least one contig that matches a non-Hexapoda barcode. The non-Hexapoda barcodes fall into 20 phylum/class categories, as shown in **Supporting Information S3 Table**.

Four of these 34 transcriptomes contained one or more negative strand RNA OKIAV, comprising a total of nine negative strand RNA viruses out of a total of 488 (1.8%) negative strand RNA viruses identified overall. Details of these four assemblies, their nine OKIAV viruses, and the matched non-Hexapoda barcodes are shown in **Supporting Information S4 Table**. Of the nine viruses, four are shown in the phylogenies in **Fig 1**, marked with an empty triangle to indicate the presence of a non-Hexapoda barcode in the associated assembly.

## Supporting information

S1 Text Supplementary results and discussion text. (DOCX)

S1 Table Additional data for the identified viral genomes. Information such as the host taxonomy, insect sample location, and collection date is provided.
(XLSX)

S2 Table Read count and insect transcriptome library size for the full and nearly-full OKIAV genomes. (XLSX)

S3 Table Non-Hexapoda COI barcodes found across all insect transcriptome assemblies. Twenty combinations of non-Hexapoda phylum/class were found across all 1243 assemblies. Matches were required to be of at least 500 nucleotides, with at least a 98% nucleotide identity level.
(XLSX)

S4 Table Detection of non-Hexapoda COI barcodes. Four assemblies (from which a total of nine OKIAV viruses were recovered) contained contigs that matched non-Hexapoda barcode sequences from the GBOL and NCBI databases (see Materials and Methods). Four of those nine viruses appear in phylogenies in **Fig 1**, where they are marked with an empty triangle. The five other viruses, indicated by asterisks, do not appear in **Fig 1** because they were not included in trees due to criteria of length, quality, and uniqueness. The table columns show, in order: the assembly identifier, the list of OKIAV viruses found in the assembly, the phylum and class of the non-Hexapoda organism(s) whose barcode was matched, and then for both GBOL and NCBI databases (when matches were found) the percentage nucleotide identity of the match and the length of the match.
(XLSX)

S1 Fig Viruses pertaining to *Rhabdoviridae*. Maximum likelihood phylogenies based on RAxML. Black branches show ICTV-accepted taxa, grey branches show unclassified taxa, and red branches show OKIAVs. Columns on the right summarize contig length, genome completeness, taxonomic grouping of hosts, and viral genus and family. The outgroup taxon (not shown) is *Mammalian 1 orthobornavirus* (*Bornaviridae*). Analyses based on PhyML and MrBayes can be found in **S2** and **S3 Figs**.
(TIF)

S2 Fig Maximum likelihood phylogenies with PhyML of viruses pertaining to *Rhabdoviridae*. Black branches show ICTV-accepted taxa, grey branches show unclassified taxa, and red branches show OKIAVs. Columns on the right side summarize contig length, genome completeness, taxonomic grouping of hosts, and viral genus and family. The outgroup taxon not shown) is *Mammalian 1 orthobornavirus* (*Bornaviridae*).
(TIF)

S3 Fig Bayesian phylogeny inference with MrBayes of viruses pertaining to *Rhabdoviridae*. Black branches show ICTV-accepted taxa, grey branches show unclassified taxa, and red branches show OKIAVs. Columns on the right side summarize contig length, genome completeness, taxonomic grouping of hosts, and viral genus and family. The outgroup taxon not shown) is *Mammalian 1 orthobornavirus* (*Bornaviridae*).
(TIF)

S4 Fig Alignment of *Almendravirus* viroporins and the potential precursor ORF of Hymenopteran almendra-related virus OKIAV1. Reference sequences belong to *Arboretum almendravirus* (ABTV), *Puerto Almendras almendravirus* (PTAMV), *Coot Bay almendravirus* (CBV), *Balsa almendravirus* (BALV), and *Rio Chico almendravirus* (RCHV). The hydrophobic stretches with multiple leucins (L) and isoleucins (I) interact with the cell membrane to facilitate cell entry [1].
(TIF)

S5 Fig Subtree of the sister clade of *Cyto*-, *Nucleo*-, *Dichorhabdo*-, and *Varicosavirus*. Maximum likelihood phylogenies based on RaxML (A) and Bayesian inference of phylogeny with MrBayes (B). Grey branches show unclassified taxa, and red branches show OKIAVs. Columns on the right side summarize contig length, genome completeness, and taxonomic grouping of hosts.
(TIF)

S6 Fig Viruses pertaining to *Xinmoviridae, Nyamiviridae, Bornaviridae, Artoviridae, Lispiviridae, Paramyxoviridae, Sunviridae, Filoviridae*, and *Pneumoviridae*. Maximum likelihood phylogenies based on RAxML. Black branches show ICTV-accepted taxa, grey branches show unclassified taxa, and red branches show OKIAVs. Columns on the right summarize contig length, genome completeness, taxonomic grouping of hosts, and viral genus and family. The outgroup taxon (not shown) is *Salmonid rhabdovirus* (*Rhabdoviridae*). Analyses based on PhyML and MrBayes can be found in **S7** and **S8 Figs**.
(TIF)

S7 Fig Maximum likelihood phylogeny with PhyML of viruses pertaining to *Xinmoviridae, Nyamiviridae, Bornaviridae, Artoviridae, Lispiviridae, Paramyxoviridae, Sunviridae, Filoviridae*, and *Pneumoviridae*. Black branches show ICTV-accepted taxa, grey branches show unclassified taxa, and red branches show OKIAVs. Columns on the right side summarize contig length, genome completeness, taxonomic grouping of hosts, and viral genus and family. The outgroup taxon (not shown) is *Salmonid rhabdovirus* (*Rhabdoviridae*).
(TIF)

S8 Fig Bayesian phylogeny inference with MrBayes of viruses pertaining to *Xinmoviridae, Nyamiviridae, Bornaviridae, Artoviridae, Lispiviridae, Paramyxoviridae, Sunviridae, Filoviridae*, and *Pneumoviridae*. Black branches show ICTV-accepted taxa, grey branches show unclassified taxa, and red branches show OKIAVs. Columns on the right side summarize contig length, genome completeness, taxonomic grouping of hosts, and viral genus and family. The outgroup taxon (not shown) is *Salmonid rhabdovirus* (*Rhabdoviridae*).
(TIF)

S9 Fig Viruses pertaining to *Chuviridae*, *Qinviridae*, and *Yueviridae*. Maximum likelihood phylogenies based on RAxML. Black branches show ICTV-accepted taxa, grey branches show unclassified taxa, and red branches show OKIAVs. Columns on the right summarize contig length, genome completeness, number of segments, taxonomic grouping of hosts, and viral genus and family. Genomic protein-coding regions are: R = RdRp, G = glycoprotein, N = nucleoprotein, Hy = hypothetical protein with unknown function. Segment length and organization are shown in parentheses: linear (L) or circular (C). For linear segments, information on the segment ends is given as: (n) = segment ends not matching the ends of the RdRp segment, (y) = segment ends matching the ends of the RdRp segment, (p) = segment ends partially matching the ends of the RdRp segment. The tree is rooted to *Yuevirus* (*Yueviridae*). Analyses based on PhyML and MrBayes can be found in **S10** and **S11 Figs**.
(TIF)

S10 Fig Maximum likelihood phylogeny with PhyML of viruses pertaining to *Chuviridae*, *Qinviridae*, and *Yueviridae*. Black branches show ICTV-accepted taxa, grey branches show unclassified taxa, and red branches show OKIAVs. Columns on the right side summarize contig length, genome completeness, number of segments, taxonomic grouping of hosts, and viral genus and family. Genomic protein-coding regions are: R = RdRp, G = glycoprotein, N = nucleoprotein, Hy = hypothetical protein with unknown function. Segment length and organization are shown in parentheses: linear (L) or circular (C). For linear segments, information on the sequence similarity of segment ends among different genome segments is given as well: (n) = segment ends not matching the ends of the RdRp segment, (y) = segment ends matching the ends of the RdRp segment, (p) = segment ends partially matching the ends of the RdRp segment. The tree is rooted to *Yuevirus* (*Yueviridae*).
(TIF)

S11 Fig Bayesian phylogeny inference with MrBayes of viruses pertaining to *Chuviridae*, *Qinviridae*, and *Yueviridae*. Black branches show ICTV-accepted taxa, grey branches show unclassified taxa, and red branches show OKIAVs. Columns on the right side summarize contig length, genome completeness, number of segments, taxonomic grouping of hosts, and viral genus and family. Genomic protein-coding regions are: R = RdRp, G = glycoprotein, N = nucleoprotein, Hy = hypothetical protein with unknown function. Segment length and organization are shown in parentheses: linear (L) or circular (C). For linear segments, information on the sequence similarity of segment ends among different genome segments is given as well: (n) = segment ends not matching the ends of the RdRp segment, (y) = segment ends matching the ends of the RdRp segment, (p) = segment ends partially matching the ends of the RdRp segment. The tree is rooted to *Yuevirus* (*Yueviridae*).
(TIF)

S12 Fig Viruses pertaining to *Orthomyxoviridae*. Maximum likelihood phylogenies based on RAxML. Black branches show ICTV-accepted taxa, grey branches show unclassified taxa, and red branches show OKIAVs. Columns on the right summarize contig length, genome completeness, number of segments, taxonomic grouping of hosts, and viral genus and family. Genomic protein-coding regions are: PB1 = polymerase subunit PB1, PB2 = polymerase subunit PB2, PA = polymerase subunit PA, G = glycoprotein, N = nucleoprotein, H = hemagglutinin, NA = neuraminidase, M = matrix protein, NS = non-structural protein, and Hy = hypothetical protein with unknown function. Segment lengths are shown in parentheses. Information on the segment ends is indicated by: (n) = segment ends not matching the ends of the PB1 segment, (y) = segment ends matching the ends of the PB1 segment, (p) = segment ends partially matching the ends of the PB1 segment. The outgroup taxon (not shown) is *Salmon isavirus* (*Isavirus*, *Orthomyxoviridae*). Analyses based on PhyML and MrBayes can be found in **S13** and **S14 Figs**.
(TIF)

S13 Fig Maximum likelihood phylogeny with PhyML of viruses pertaining to *Orthomyxoviridae*. Black branches show ICTV-accepted taxa, grey branches show unclassified taxa, and red branches show OKIAVs. Columns on the right side summarize contig length, genome completeness, number of segments, taxonomic grouping of hosts, and viral genus and family. Genomic protein-coding regions are: PB1 = polymerase subunit PB1, PB2 = polymerase subunit PB2, PA = polymerase subunit PA, G = glycoprotein, N = nucleoprotein, H = hemagglutinin, NA = neuraminidase, M = matrix protein, NS = non-structural protein, and Hy = hypothetical protein with unknown function. Segment lengths are shown in parentheses. Information on the sequence similarity of segment ends among different genome segments is given as well: (n) = segment ends not matching the ends of the PB1 segment, (y) = segment ends matching the ends of the PB1 segment, (p) = segment ends partially matching the ends of the PB1 segment. The outgroup taxon (not shown) is *Salmon isavirus* (*Isavirus*, *Orthomyxoviridae*).
(TIF)

S14 Fig Bayesian phylogeny inference with MrBayes of viruses pertaining to *Orthomyxoviridae*. Black branches show ICTV-accepted taxa, grey branches show unclassified taxa, and red branches show OKIAVs. Columns on the right side summarize contig length, genome completeness, number of segments, taxonomic grouping of hosts, and viral genus and family. Genomic protein-coding regions are: PB1 = polymerase subunit PB1, PB2 = polymerase subunit PB2, PA = polymerase subunit PA, G = glycoprotein, N = nucleoprotein, H = hemagglutinin, NA = neuraminidase, M = matrix protein, NS = non-structural protein, and Hy = hypothetical protein with unknown function. Segment lengths are shown in parentheses. Information on the sequence similarity of segment ends among different genome segments is given as well: (n) = segment ends not matching the ends of the PB1 segment, (y) = segment ends matching the ends of the PB1 segment, (p) = segment ends partially matching the ends of the PB1 segment. The outgroup taxon (not shown) is *Salmon isavirus* (*Isavirus*, *Orthomyxoviridae*).
(TIF)

S15 Fig Phylogenetic co-segregation between PB1 and PB2, PB1 and PA, and PB1 and nucleoprotein of the viruses pertaining to *Orthomyxoviridae*. Topologically congruent clades are highlighted in color. Branches in black indicate taxa that do not share a common topological pattern in the respective tree pairs. Not all genomic segments have been identified for all viral taxa; therefore, the number of taxa varies among the tree pairs. Clade H, mainly composed of the *Orthomyxoviridae* genera, is not congruent between the PB1 and the nucleoprotein trees and shows two different topologies within *Thogotovirus*. The PB1 segments of Coleopteran orthomyxo-related virus OKIAV196 and -200 are both found within the same insect transcriptome (*Ips typographus*). Within this transcriptome we have also identified one PB2, one PA, and one nucleoprotein segments. The co-segregation analysis allowed us to assign all the latter segments to Coleopteran orthomyxo-related virus OKIAV200 rather than -196.
In the phylogenies of PB1 and PA, 52 of 54 taxa are distributed in eight monophyletic clades. Clade B consists of three distinct inner clades that are topology-wise stable and share similarities within their inner parts. Whereas clades D and E are direct sisters in the PB1 tree, clade D is not directly linked to clade E in the PA tree. However, the PA tree has a higher support in this region of the tree. The position of clade G is maintained across all trees, except of Coleopteran orthomyxo-related virus OKIAV196 in the PA tree, which actually belongs next to Zygentoman orthomyxo-related virus OKIAV204 (clade A). Clade H consists exclusively of ICTV-accepted genera of *Orthomyxoviridae*, except *Hubei orthoptera virus 6*. These clades (G and H) show identical topology in both trees.
In the phylogenies of PB1 and PB2, 43 of 46 taxa are distributed in six monophyletic clades. Clades D and E are merely represented by Archaeognathan orthomyxo-related virus OKIAV189, Hemipteran orthomyxo-related virus OKIAV188, and Neuropteran orthomyxo-related virus OKIAV190. Clade B shows a similar topology as in PA and PB1, and Clade G maintains its phylogenetic position, except of Coleopteran orthomyxo-related virus OKIAV196 in the PB2 tree, because it belongs next to Zygentoman orthomyxo-related virus OKIAV204 of clade A.
For the nucleoprotein, 45 of 57 taxa were distributed in eight monophyletic clades. Clade G maintains its topology in this phylogeny as well, showing additionally that the identified N-segment is indeed part of Zygentoman orthomyxo-related virus OKIAV204. Clade E is a sister to clade G on the nucleoprotein tree, in contrast to the PB1 tree where it is sister to clade D.
(TIF)

S16 Fig Viruses pertaining to *Hantaviridae, Cruliviridae, Peribunyaviridae*, and *Fimoviridae*. Maximum likelihood phylogenies based on RAxML. Black branches show ICTV-accepted taxa, grey branches show unclassified taxa, and red branches show OKIAVs. The outgroup taxon (not shown) is Collembolan hanta-related virus OKIAV223 (shown in **S20 Fig**). Analyses based on PhyML and MrBayes can be found in **S17** and **S18 Figs**.
(TIF)

S17 Fig Maximum likelihood phylogeny with PhyML of viruses pertaining to *Hantaviridae, Cruliviridae, Peribunyaviridae*, and *Fimoviridae*. Black branches show ICTV-accepted taxa, grey branches show unclassified taxa, and red branches show OKIAVs. Columns on the right side summarize contig length, genome completeness, number of segments, taxonomic grouping of hosts, and viral genus and family. Genomic protein-coding regions are: R = RdRp, G = glycoprotein, N = nucleoprotein, Hy = hypothetical protein with unknown function. Segment lengths are shown in parentheses. Information on sequence similarity of the segment ends among different genome segments is given as well: (n) = segment ends not matching the ends of the RdRp segment, (y) = segment ends matching the ends of the RdRp segment, (p) = segment ends partially matching the ends of the RdRp segment. The outgroup taxon (not shown) is Collembolan hanta-related virus OKIAV223 (shown in **S20 Fig**).
(TIF)

S18 Fig **Maximum likelihood phylogeny based on RAxML (A) and Bayesian phylogeny inference with MrBayes (B) of viruses pertaining to *Hantaviridae*, *Cruliviridae*, *Peribunyaviridae*, and *Fimoviridae*.** Black branches show ICTV-accepted taxa, grey branches show unclassified taxa, and red branches show OKIAVs. Columns on the right side summarize contig length, genome completeness, number of segments, taxonomic grouping of hosts, and viral genus and family. Genomic protein-coding regions are: R = RdRp, G = glycoprotein, N = nucleoprotein, Hy = hypothetical protein with unknown function. Segment lengths are shown in parentheses. Information on sequence similarity of the segment ends among different genome segments is given as well: (n) = segment ends not matching the ends of the RdRp segment, (y) = segment ends matching the ends of the RdRp segment, (p) = segment ends partially matching the ends of the RdRp segment. The outgroup taxon (not shown) is Collembolan hanta-related virus OKIAV223 (shown in **S20 Fig**).
(TIF)

S19 Fig Phylogenetic co-segregation between RdRp and glycoprotein, and RdRp and nucleoprotein of the viruses pertaining to *Hantaviridae, Cruliviridae, Peribunyaviridae*, and *Fimoviridae*. Topologically congruent clades are highlighted in color. Branches in black indicate taxa that do not share a common topological pattern in the respective tree pairs.
In the phylogenies of RdRp and glycoprotein, 55 of 59 taxa were distributed in five monophyletic clades represented by the genera *Orthobunyavirus*, *Herbevirus*, *Tospovirus*, *Orthohantavirus*, and *Emaravirus*. Despite spanning four different families, the phylogenies are well-supported and some of the (sub)topologies can be confirmed. *Tospovirus* and *Emaravirus* have a completely congruent topology, while *Orthobunyavirus* and *Orthohantavirus* have some topologically stable subclades. Noteworthy are not only the congruent topologies, but also the very similar branch lengths of *Tospovirus*, *Orthohantavirus*, and *Emaravirus*. Based only on the tree topology, an assignment of the M-segment to either Dipluran hanta-related virus OKIAV217 or -218 cannot be assessed. In the phylogenies of RdRp and nucleoprotein, 44 of 49 taxa distributed in the genera *Orthobunyavirus*, *Herbevirus*, *Tospovirus*, *Orthohantavirus*, and *Emaravirus*. *Tospovirus* is the only genus that retains its inner topological structure among all trees, except of the position of *Bean necrotic mosaic virus*, that in the nucleoprotein tree is sister to all other tospoviruses. However, the position of *Tospovirus* within *Peribunyaviridae* is not supported by the nucleoprotein phylogeny. Apart from that, all genera are still monophyletic. The position of *Khurdun virus* as the first split from *Herbevirus* can be confirmed by both nucleoprotein and glycoprotein phylogenies.
(TIF)

S20 Fig Representative viruses of *Bunyavirales*. Bayesian inference of phylogeny based on MrBayes. Black branches show selected reference taxa, and red branches show some OKIAVs.
(TIF)

S21 Fig Viruses pertaining to *Phasmaviridae*. Maximum likelihood phylogenies based on RAxML. Black branches show ICTV-accepted taxa, grey branches show unclassified taxa, and red branches show OKIAVs. Columns on the right summarize contig length, genome completeness, number of segments, taxonomic grouping of hosts, and viral genus and family. Genomic protein-coding regions are: R = RdRp, G = glycoprotein, N = nucleoprotein, Hy = hypothetical protein with unknown function. Segment lengths are shown in parentheses. Information on the segment ends is indicated by: (n) = segment ends not matching the ends of the RdRp segment, (y) = segment ends matching the ends of the RdRp segment, (p) = segment ends partially matching the ends of the RdRp segment. The tree was rooted using a hantavirus outgroup. The outgroup was then removed, the tree recalculated, and the rooting between *Orthophasmavirus* vs. (HAF, *Feravirus*, *Wuhivirus*, and *Jonvirus)* was maintained. Analyses based on PhyML and MrBayes can be found in **S22** and **S23 Figs**.
(TIF)

S22 Fig Maximum likelihood phylogeny with PhyML of viruses pertaining to *Phasmaviridae*. Black branches show ICTV-accepted taxa, grey branches show unclassified taxa, and red branches show OKIAVs. Columns on the right side summarize contig length, genome completeness, number of segments, taxonomic grouping of hosts, and viral genus and family. Genomic protein-coding regions are: R = RdRp, G = glycoprotein, N = nucleoprotein, Hy = hypothetical protein with unknown function. Segment lengths are shown in parentheses. Information on sequence similarity of the segment ends among different genome segments is given as well: (n) = segment ends not matching the ends of the RdRp segment, (y) = segment ends matching the ends of the RdRp segment, (p) = segment ends partially matching the ends of the RdRp segment. The tree is rooted to the exclusion of the lower clade of *Phasmaviridae* (HAF, *Feravirus*, *Wuhivirus*, *Jonvirus*) following preliminary analyses using a hantavirus outgroup.
(TIF)

S23 Fig Bayesian phylogeny inference with MrBayes of viruses pertaining to *Phasmaviridae*. Black branches show ICTV-accepted taxa, grey branches show unclassified taxa, and red branches show OKIAVs. Columns on the right side summarize contig length, genome completeness, number of segments, taxonomic grouping of hosts, and viral genus and family. Genomic protein-coding regions are: R = RdRp, G = glycoprotein, N = nucleoprotein, Hy = hypothetical protein with unknown function. Segment lengths are shown in parentheses. Information on sequence similarity of the segment ends among different genome segments is given as well: (n) = segment ends not matching the ends of the RdRp segment, (y) = segment ends matching the ends of the RdRp segment, (p) = segment ends partially matching the ends of the RdRp segment. The tree is rooted to the lower clade of *Phasmaviridae* (HAF, *Feravirus*, *Wuhivirus*, *Jonvirus*) following preliminary analyses using a hantavirus outgroup.
(TIF)

S24 Fig Phylogenetic co-segregation between RdRp and glycoprotein, and RdRp and nucleoprotein of the viruses pertaining to *Phasmaviridae*. Topologically congruent clades are highlighted in color. Branches in black indicate taxa that do not share a common topological pattern in the respective tree pairs. In the phylogenies of RdRp and nucleoprotein, 34 of 40 taxa were distributed in three monophyletic clades that consist of the ICTV-accepted genera *Feravirus*, *Wuhivirus*, and *Orthophasmavirus*. In the nucleoprotein phylogeny, clades DAP and HAP form inner congruent monophyletic groups within *Orthophasmavirus*. The same pattern applies to 30 of 36 taxa in the glycoprotein phylogeny. Within *Orthophasmavirus*, HAP is the most stable clade, with a subclade of five taxa that have identical topology among the phylogenies. However, the bootstrap support of the *Orthophasmavirus* subclades is below 80%. The clear subdivision into clades A and B on the RdRp and nucleoprotein trees is not verified in the glycoprotein phylogeny, yet *Feravirus* and HAF group together with a high support (99%), and maintain their inner topological structures. Most of the *Orthophasmavirus* taxa have not been subjected to laboratory studies. Additionally, the OKIAV sequences are only fragmentarily assembled. Obtaining stable and congruent phylogenies among genomic segments can thus not be expected.
(TIF)

S25 Fig Viruses pertaining to *Phenuiviridae*. Maximum likelihood phylogenies based on RAxML. Black branches show ICTV-accepted taxa, grey branches show unclassified taxa, and red branches show OKIAVs. Columns on the right summarize contig length, genome completeness, number of segments, taxonomic grouping of hosts, and viral genus and family. Genomic protein-coding regions are: R = RdRp, G = glycoprotein, N = nucleoprotein, Hy = hypothetical protein with unknown function. Segment lengths are shown in parentheses. Information on the segment ends is indicated by: (n) = segment ends not matching the ends of the RdRp segment, (y) = segment ends matching the ends of the RdRp segment, (p) = segment ends partially matching the ends of the RdRp segment. The tree is rooted to the putative subfamily. Analyses based on PhyML and MrBayes can be found in **S26** and **S27 Figs**.
(TIF)

S26 Fig Maximum likelihood phylogeny with PhyML of viruses pertaining to *Phenuiviridae*. Black branches show ICTV-accepted taxa, grey branches show unclassified taxa, and red branches show OKIAVs. Columns on the right side summarize contig length, genome completeness, number of segments, taxonomic grouping of hosts, and viral genus and family. Genomic protein-coding regions are: R = RdRp, G = glycoprotein, N = nucleoprotein, Hy = hypothetical protein with unknown function. Segment lengths are shown in parentheses. Information on sequence similarity of the segment ends among different genome segments is given as well: (n) = segment ends not matching the ends of the RdRp segment, (y) = segment ends matching the ends of the RdRp segment, (p) = segment ends partially matching the ends of the RdRp segment. The tree is rooted to the putative subfamily.
(TIF)

S27 Fig Bayesian phylogeny inference with MrBayes of viruses pertaining to *Phenuiviridae*. Black branches show ICTV-accepted taxa, grey branches show unclassified taxa, and red branches show OKIAVs. Columns on the right side summarize contig length, genome completeness, number of segments, taxonomic grouping of hosts, and viral genus and family. Genomic protein-coding regions are: R = RdRp, G = glycoprotein, N = nucleoprotein, Hy = hypothetical protein with unknown function. Segment lengths are shown in parentheses. Information on sequence similarity of the segment ends among different genome segments is given as well: (n) = segment ends not matching the ends of the RdRp segment, (y) = segment ends matching the ends of the RdRp segment, (p) = segment ends partially matching the ends of the RdRp segment. The tree is rooted to the putative subfamily.
(TIF)

S28 Fig Phylogenetic co-segregation between RdRp and glycoprotein, and RdRp and nucleoprotein of the viruses pertaining to *Phenuiviridae*. Topologically congruent clades are highlighted in color. Branches in black indicate taxa that do not share a common topological pattern in the respective tree pairs. The lack of complete genomes for most of the taxa that appear on the trees causes high topological conflict between the phylogenies of the different segments. The glycoprotein- and nucleoprotein-segments have not been identified for most of the OKIAV. The bootstrap support on the clades of the single-species genera *Hudovirus*, *Pidchovirus*, *Hudivirus*, *Beidivirus*, and *Horwuvirus* is low in comparison to the rest of the tree. Thus, the phylogenetic signal is probably not sufficient to draw meaningful conclusions on co-segregations for these genera. Additionally, within the putative subfamily, the lack of genomic segments for co-segregation analysis does not allow us drawing conclusions either.
In the phylogenies of RdRp and nucleoprotein, 45 of 64 taxa are distributed in topologically stable monophyletic clades within the genera *Phasivirus*, *Wubeivirus*, *Tenuivirus*, *Phlebovirus* (with the exception of clade B), *Banyangvirus*, *Goukovirus*, and additionally the unclassified clades A, E, and F. Within *Phlebovirus*, clade B is sister to clade A and includes the genus *Tenuivirus*. Clade C is topologically congruent among all three phylogenies. Both clades C and D maintain their taxa composition across all three trees as well as their relation to *Banyangvirus*. The topological stability of the *Banyangvirus* clade within the *Phlebovirus* clade, suggests that *Banyangvirus* should rather be classified as a sub-genus of *Phlebovirus*.
In the phylogenies of RdRp and glycoprotein, 35 of 41 taxa are distributed in topologically stable monophyletic clades that are accepted genera and the unclassified clades D, E, and F. *Hudivirus* and *Beidivirus* group together in this case, indicating a close relationship. A doubtful classification is the one of *Wubeivirus*: it is monophyletic only in the glycoprotein phylogeny, but groups consistently with *Phasivirus* in all phylogenies.
(TIF)

S29 Fig Phylogenetic co-segregation between RdRp and glycoprotein, and RdRp and nucleoprotein of the viruses pertaining to *Phlebovirus* and *Banyangvirus*. Topologically congruent clades are highlighted in color. Branches in black indicate taxa that do not share a common topological pattern in the respective tree pairs. *Banyangvirus* is sister to the main *Phlebovirus* clades in the RdRp phylogeny, indicating that *Banyangvirus* should not be regarded as an independent genus.
(TIF)

S30 Fig Viruses pertaining to *Arenaviridae, Mypoviridae, Nairoviridae*, and *Wupedeviridae*. Maximum likelihood phylogenies based on RAxML. Black branches show ICTV-accepted taxa, grey branches show unclassified taxa, and red branches show OKIAVs. Columns on the right summarize contig length, genome completeness, number of segments, taxonomic grouping of hosts, and viral genus and family. Genomic protein-coding regions are: R = RdRp, G = glycoprotein, N = nucleoprotein, Hy = hypothetical protein with unknown function. Segment lengths are shown in parentheses. Information on the segment ends is indicated by: (n) = segment ends not matching the ends of the RdRp segment, (y) = segment ends matching the ends of the RdRp segment, (p) = segment ends partially matching the ends of the RdRp segment. The outgroup taxon (not shown) is *Rift Valley fever phlebovirus* (*Phenuiviridae*). Analyses based on PhyML and MrBayes can be found in **S31** and **S32 Figs**.
(TIF)

S31 Fig Maximum likelihood phylogeny with PhyML of viruses pertaining to *Arenaviridae, Mypoviridae, Nairoviridae*, and *Wupedeviridae*. Black branches show ICTV-accepted taxa, grey branches show unclassified taxa, and red branches show OKIAVs. Columns on the right side summarize contig length, genome completeness, number of segments, taxonomic grouping of hosts, and viral genus and family. Genomic protein-coding regions are: R = RdRp, G = glycoprotein, N = nucleoprotein, Hy = hypothetical protein with unknown function. Segment lengths are shown in parentheses. Information on sequence similarity of the segment ends among different genome segments is given as well: (n) = segment ends not matching the ends of the RdRp segment, (y) = segment ends matching the ends of the RdRp segment, (p) = segment ends partially matching the ends of the RdRp segment. The outgroup taxon (not shown) is *Rift Valley fever phlebovirus* (*Phenuiviridae*).
(TIF)

S32 Fig Bayesian phylogeny inference with MrBayes of viruses pertaining to *Arenaviridae, Mypoviridae, Nairoviridae*, and *Wupedeviridae*. Black branches show ICTV-accepted taxa, grey branches show unclassified taxa, and red branches show OKIAVs. Columns on the right side summarize contig length, genome completeness, number of segments, taxonomic grouping of hosts, and viral genus and family. Genomic protein-coding regions are: R = RdRp, G = glycoprotein, N = nucleoprotein, Hy = hypothetical protein with unknown function. Segment lengths are shown in parentheses. Information on sequence similarity of the segment ends among different genome segments is given as well: (n) = segment ends not matching the ends of the RdRp segment, (y) = segment ends matching the ends of the RdRp segment, (p) = segment ends partially matching the ends of the RdRp segment. The outgroup taxon (not shown) is *Rift Valley fever phlebovirus* (*Phenuiviridae*).
(TIF)

S33 Fig Phylogenetic co-segregation between RdRp and glycoprotein, and RdRp and nucleoprotein of the viruses pertaining to *Arenaviridae, Mypoviridae, Nairoviridae*, and *Wupedeviridae*. Topologically congruent clades are highlighted in color. Branches in black indicate taxa that do not share a common topological pattern in the respective tree pairs.
In the phylogenies of RdRp and nucleoprotein, 31 of 38 taxa are distributed in the monophyletic genera *Orthonairovirus*, *Reptarenavirus*, and *Mammarenavirus*. *Reptarenavirus* is the only genus that consistently has a congruent topology among all trees. There are very few viruses that are not formally accepted by ICTV in this tree, and the ones that are new fit well in between the established genera, resulting in a stable backbone of the phylogeny. In the phylogenies of RdRp and glycoprotein, 30 of 39 taxa are distributed in *Orthonairovirus*, *Reptarenavirus*, and *Mammarenavirus*. The glycoprotein phylogeny shows similar topology to the nucleoprotein one, but only *Reptarenavirus* maintains its topological position. The biggest disagreement is the positioning of *Reptarenavirus* within the *Nairoviridae* clade. The backbones of the trees are not in agreement, therefore the positions of the single-species genera *Shaspivirus*, *Wumivirus*, and *Hubevirus* cannot be confirmed. However, in both phylogenies, the positions of *Striwavirus*, Blattodean nairo-related virus OKIAV321, and *Xinzhou spider virus* are stable.
(TIF)

S34 Fig Read mapping on the genome of Odonatan chu-related virus OKIAV137. The sequence is joined head-to-tail, genome start and end are colored and indicated by arrows. The end-to-start gap is solely bridged by two flanking nucleotides of four reads (marked in red). The ORFs encoding for glycoprotein (G), nucleoprotein (N), and RdRp, as well as the read coverage are shown.
(TIF)
