# Supplementary material for: Re-assessing the diversity of negative strand RNA viruses in insects
Source: PLoS Pathog. 2019 Dec 12;15(12):e1008224. doi: 10.1371/journal.ppat.1008224 (PMC6932829; doi:10.1371/journal.ppat.1008224)
Supplement: S1 Text — (DOCX) [file ppat.1008224.s001.docx]

**Supporting Information**

**S1 Text: Supplementary results and discussion text.**

***Rhabdoviridae***

This family includes 18 genera (**S1 Fig**). The tree has a bifurcation from which mainly plant-associated viruses, such as those assigned to the genera *Cytorhabdovirus* and *Nucleorhabdovirus*, split to the exclusion of animal viruses, such as those assigned to the genera *Sigmavirus* and *Lyssavirus*. The genus *Novirhabdovirus* is a phylogenetic outlier.

**S1 Fig** shows 33 selected novel rhabdovirus sequences from the present study that do not cluster with members of the established genera. A comparatively small number of viruses are grouped with dimarhabdoviruses, which is a historical classification for a now paraphyletic group of viruses found in diptera and mammalia. Full genomes of novel dimarhabdovirus-related viruses (Lepidopteran rhabdo-related virus OKIAV12 and Hymenopteran rhabdo-related virus OKIAV8) suggest a typical rhabdovirus gene order comprising nucleoprotein - phosphoprotein - matrix protein - glycoprotein - large protein (including RdRp, **Fig 3**).

A particularly interesting finding is Hymenopteran almendra-related virus OKIAV1, a full genome with typical rhabdovirus organization (**Fig 3**). It is positioned in sister relationship to all members of *Almendravirus*, a recently established genus [1]. The type isolates stem from mosquitoes (order Diptera), while Hymenopteran almendra-related virus OKIAV1 is from a hymenopteran species, thus pertaining to a sister order to Diptera. Almendraviruses have a viroporin protein encoded between the G and L genes [1]; Hymenopteran almendra-related virus OKIAV1 has a region of hydrophobic residues, as in viroporins, but none of the remaining salient features and no start codon (**S4 Fig**). Degeneration of a viroporin gene after divergence from a common ancestor with almendraviruses seems possible. Hymenopteran almendra-related virus OKIAV1 and almendraviruses provide one of the many examples of putative virus-host co-segregation in negavirus evolution.

A multitude of novel rhabdovirus sequences branch in sister relationship to a clade formed by cyto-, nucleo-, dichorhabdo-, as well as varicosaviruses (**S1 Fig**). The clades shown are well-supported but different phylogenetic algorithms yield alternative topologies. We therefore conducted additional phylogenetic studies focused only on this clade, as shown in **S5 Fig**. The resulting tree suggests the need for at least eight distinct taxa comprising 14 full or partial sequences recently identified in metagenomic studies [2, 3], as well as 12 full and 14 partial genome sequences from the present study. All have typical rhabdovirus genome architectures. Five of these tentative genera are defined by the present study (**Table 1**).

***Xinmoviridae***

The novel family *Xinmoviridae* comprises only the formerly unassigned genus *Anphevirus*. We find 13 OKIAVs related to this family (**S6 Fig**). Five sequences represent complete genomes (**Fig 3**). These sequences stem from a broad range of hosts, belonging to 13 orders of insects (Blattodea, Coleoptera, Diptera, Hemiptera, Hymenoptera, Lepidoptera, Mantodea, Neuroptera, Odonata, Orthoptera, Psocodea, Trichoptera, Zygentoma; consult **S1 Table** for geographic origins). While the family is not formally subdivided into genera, a deep topological separation and structured host association emerges after addition of data from the present study (**S6 Fig**). To orient future taxonomic classification, we have assigned six different *Anphevirus* lineages, two of which contain the sequences previously detected in a mosquito and a fly [2], as well as in pooled insects [3]. Two clades can be considered to define potential novel genera based on full genome data from the present study (**Table 1**). Lineage I appears to be associated with Hymenoptera, Hemiptera, and Diptera, while lineage III is specific for Odonata. Lineage I contains endogenous viral elements of mosquitos but is represented by full genomes from other insect orders. Together with the recent reconciliation of insect phylogeny that incorporates molecular data as well as the fossil record [4], this provides an interesting scenario for studies of viral germline integration.

***Nyamiviridae*** is a newly-defined family that was recently extended to contain six genera [5]. In the genus *Nyavirus*, the type species *Nyamanini-* and *Midway nyavirus* are both known to infect birds and ticks, causing phenotypic changes typical of arboviruses [6, 7]. *Sierra Nevada nyavirus* is another virus that was recently assigned to *Nyavirus*, and is known to infect ticks [8]. In the genus *Socyvirus*, the only known member is *Soybean cyst nematode socyvirus* that replicates and is detected in at least two developmental stages of nematodes [9]. No biological knowledge is available for any member of the genera *Tapwovirus*, *Orinovirus*, *Crustavirus*, and *Berhavirus*, which are derived from metagenomic studies [3, 10]. As shown in **S6 Fig**, our data contribute four novel viruses related to the monospecific genus *Orinovirus*, two of them with full genomes. Lepidopteran orino-related virus OKIAV84 is the closest relative to *Orinoco* *orinovirus* and stems from an insect of the same order (Lepidoptera, although from a different family) [4]. Hymenopteran orino-related virus OKIAV85, -87, and -88 form a monophyletic clade and are associated with Hymenoptera. A very recent sequence description from ants (Formica fusca virus 1) also falls in this clade, providing independent confirmation of association with Hymenoptera [10]. The deep divergence and consistent association with a specific insect order suggests this clade may constitute a separate genus (termed “*Orinovirus* lineage 1” in **S6 Fig**). In that case, only Lepidopteran orino-related virus OKIAV84 would group with the genus *Orinovirus* (termed “*Orinovirus* lineage II” in **S6 Fig**).

The family ***Bornaviridae*** has three established genera (*Orthobornavirus*, *Carbovirus*, and *Cultervirus*) that occur in mammals, birds, fish, and reptiles. The absence of bornaviruses in our extensive insect dataset is compatible with a divergence of these viruses in primordial vertebrates that pre-dated extant reptiles and mammals.

The family ***Artoviridae*** only contains one genus, *Peropuvirus*. The type species is *Pteromalus puparum peropuvirus*, which is vertically transmitted in parasitoid wasps and regulates the sex ratio by decreasing the number of female offspring [11]. Our data add three novel viruses associated with three orders of insects (Diptera, Hymenoptera, Trichoptera). The closest relative to *Pteromalus puparum peropuvirus*, Hymenopteran arto-related virus OKIAV1338, also stems from a parasitoid wasp. The other host associations suggest that further subdivision of the family *Artoviridae* should be considered. Based on host order associations, for instance, phylogeny suggests the existence of three additional genera in insects. However, classification is impossible at this point because of the lack of full genome sequences.

The family ***Lispiviridae*** contains the formerly unassigned genera *Arlivirus*, *Chengtivirus*, and *Wastrivirus*, detected in a tick, a spider, and a water strider in China [2]. The International Committee on Taxonomy of Viruses (ICTV) established and subsequently withdrew a part of these genera within the last two years [5, 12]. The novel family *Lispiviridae* is now monogeneric and defined by six type species that have all been associated with the genus *Arlivirus*. The present study adds viruses from 11 orders of insects sampled worldwide (Archaeognatha, Blattodea, Coleoptera, Diptera, Hemiptera, Hymenoptera, Isoptera, Megaloptera, Neuroptera, Odonata, and Strepsiptera). As shown in the phylogenetic tree in **S6 Fig**, we have identified 13 viral sequences, four of which have complete genomes (**Fig 3**). The host associations of insect viruses in the present data suggest a criterion for separating this family into nine distinct genetic lineages at the genus level. Lineage III is specific for Hymenoptera, lineage IV for Hemiptera, and lineage VII for Odonata. Four full genomes of lispiviruses were obtained. Due to the phylogenetic distance, genome annotation is challenging, and it remains unknown which proteins other than G and L are encoded [10, 13]. Availability of full genomes justifies formal consideration of two of these lineages as newly discovered putative genera (lineages III, IV in **S6 Fig**, **Table 1**). Interestingly, the hymenopteran association of lineage III was very recently confirmed by a sequence termed Linepithema humile rhabdo-likevirus 1, found in ants [13].

***Mymonaviridae***

The fungal mononegavirus *Sclerotinia sclerotimonavirus* is the type species of the monogeneric family *Mymonaviridae* (genus *Sclerotimonavirus* [14]). With the exception of *Hubei sclerotimonavirus,* found in a mix of arthropods from the Hubei province in China [3], all other mymonaviruses are fungal viruses. Our study does not identify any mymonavirus in insects.

***Filo-*, *Paramyxo-*, and *Pneumoviridae***

The families *Filoviridae* (genera *Ebolavirus*, *Marburgvirus*, *Cuevavirus, Striavirus, Thamnovirus*), *Paramyoxoviridae* (subfamilies *Avula-, Metaparamyxo-, Orthparamyxo-, and Rubulavirinae*), *Pneumoviridae* (genera *Metapneumovirus* and *Orthopneumovirus*), and *Sunviridae* (genus *Sunshinevirus*) form a monophyletic clade of vertebrate-pathogenic virus families. While earlier studies in mammals and other vertebrates have contributed a significant extension of the diversity of *Paramyxo-* and *Pneumoviridae* [15, 16], the present study, covering an extremely broad host genetic space, does not yield any insect-associated members of these families.

***Chuviridae***

The family *Chuviridae* is a novel family of arthropod-associated viruses that has been ascribed as sole element of the novel class *Monjiviricetes* and the novel order *Jingchuvirales*. The only genus in the family, termed *Mivirus*, has 29 species. The genomes of chuviruses were found to appear in linear, circular, and segmented circular forms [2]. **S9 Fig** shows an additional 25 exemplary sequences from the present study, including seven with at least one full segment and one with two full segments. We have encountered 10 chuviruses with segmented genomes.

Most, or all, of the chuviral sequences discovered in the present study are diversified enough to define independent species. The addition of our data reveals a structure of three major clades within *Chuviridae* (A, B, and C in **S9 Fig**), each divided into monophyla associated with insects and lineages in other non-vertebrate hosts. The long-branching position occupied by viruses from non-insect hosts may be a result of under-sampling of these hosts. Four subclades show associations with members of the insect orders Odonata (OAM - Odonata-associated *Mivirus*), Hemiptera (HeAM - Hemiptera-associated *Mivirus*), Hymenoptera (HyAM - Hymenoptera-associated *Mivirus*), and Phasmatodea (PAM - Phasmatodea-associated *Mivirus*). These clades provide an assessment of the depth of a genetic unit of chuviruses that could be classified as genera (**Fig 3**).

Within the OAM clade, Odonatan chu-related virus OKIAV136 and -137 have coding-complete genomes and group with *Odonate mivirus*. As well as the predicted proteins of *Odonate mivirus*, the two OKIAV genomes show nucleoprotein-coding predictions for the third ORF (**Fig 3**). Beneath the OAM clade, the sister taxon of Wenling chuvirus-like virus 1, Dermapteran chu-related virus OKIAV142, holds an extra viral segment which is predicted to encode a nucleoprotein. Dermapteran chu-related virus OKIAV142 encodes an RdRp and glycoprotein on its other genomic segment. Other miviruses of the same clade have two genomic segments but only encode RdRp and nucleoprotein. At least OAM (Odonatan chu-related virus OKIAV136 and -137 with full genomes) and HyAM (Hymenopteran chu-related virus OKIAV123, -124, and -126 with full genomes) should be considered as representatives of novel genera.

***Qinviridae***

The family *Qinviridae* is a group of novel arthropod-associated viruses that has been ascribed as sole member of the novel order *Muvirales* and to the novel class *Chunqiuviricetes*. The only genus in the family, termed *Yingvirus*, has eight species. Two species, *Wuhan yingvirus* and *Hubei yingvirus*, occur in insects while other yingviruses occur in crustaceans, helminths, and lophotrochozoa. Our study provides the first independent confirmation of the existence of *Qinviridae* by adding the longest so-far known qinvirus sequence (Collembolan qin-related virus OKIAV112) that stems from the hexapod *Anurida maritima* (seashore springtail). It is equidistant from the aforementioned taxa and therefore likely constitutes a separate genus. Its genome organization agrees with the proposed bi-segmented genome organization of the non-insect *Qinviridae* (L and N-G, see **Fig 3**), and it adds a protein prediction for the second segment of insect qinviruses.

The topology of the *Qinviridae* tree may reflect that of the corresponding hosts, as viruses in sister relationship to insect-associated viruses are found in invertebrates (crustaceans, helminths, and lophotrochozoans) and insect-associated viruses show a branching pattern reflecting that of their insect hosts. This is also confirmed by the very recent description of Linepithema humile qinvirus-like virus 1 [13], an incomplete qinvirus sequence found in ants.

***Orthomyxoviridae***

The *Orthomyxoviridae* family contains seven genera: *Alpha-*, *Beta-*, *Gamma-*, and *Deltainfluenzavirus*, *Isavirus*, *Thogotovirus,* and *Quaranjavirus*, which comprise nine type species altogether. With the exception of the influenza viruses, orthomyxoviruses are transmitted by arthropod vectors [17, 18]. Isaviruses infect fish and are considered to be transmitted by sea lice [19]. Thogotoviruses and quaranjaviruses are transmitted to mammals by ticks [20]. The genus *Quaranjavirus* contains viruses that appear to cycle between soft ticks (family Argasidae) and aquatic birds [17]. All genera are summarized in **S12 Fig** and genome organizations are shown in **Fig 3**. Not included in the figure is the Tilapia virus (species *Tilapia tilapinevirus),* recently detected in tilapia aquacultures (*Oreochromis sp.*) [21]. This virus was classified into the novel viral order *Articulavirales* and the solitary monogeneric family *Amnoonviridae* (genus *Tilapinevirus*).

The segmentation of orthomyxovirus genomes makes it difficult to assemble full genome sequences based on RNAseq studies. The orthomyxovirus RdRp is encoded on three segments, PB1, PB2, and PA, of which PB1 contains the conserved motifs for our pHMM search as well as phylogeny. Our study contributes 46 viruses with up to five segments. Among those, 31 PB2 and 41 PA segments are found, thus providing a complete RdRp for 31 viral sequences. Only a minority of the viral sequences that we identified group together with members of established genera. Most of the sequences from the present study fall in previously undescribed clades. Long-branching viral taxa are mostly derived from members of basal insect groups, or outgroup taxa including Zygentoma (silverfish), Myriapoda, and Archaeognatha (jumping bristletails). Based on the criteria that genomes should at least comprise five segments, topology of several phylogenetic analyses should agree, and diversity should be comparable to that within established genera, we can define at least seven clades that may determine novel putative genera (designated O1-O7 in **S12 Fig**). Five of these are newly described in the present study and have five genome segments (**Table 1**).

The diversity and the number of assigned members of the genera *Thogotovirus* and *Quaranjavirus* are also expanded by the addition of sequences from the present study. The genome termini in seven novel viruses are partially sequenced and confirmed to be reverse complementary. However, in the absence of live virus isolates, the completeness of segmented genomes cannot be confirmed. A recently described virus (Sinu virus, [22]) is related to one of the novel viruses, Neuropteran orthomyxo-related virus OKIAV210, but not shown in the tree figure.

Phylogenies of each RdRp subunit were inferred separately and then compared to assess topological congruence (**S15 Fig**). Overall, there is congruence between segments in major clades. In some clades, such as clade C of **S15 Fig**, reassortment of at least some of the segments seems to have occurred in lineage precursors, as topological incongruence is observed for all members of the clade. In cases where individual incongruences are suggested, we cannot discriminate between *in silico* misassembly of genomes and actual reassortment based on the present data.

**Novel** ***Orthohantavirus*-related lineages**

Novel unclassified sequences related to hantaviruses have recently been detected in diverse insects [3]. The unclassified clades in sister relationship to *Hantaviridae* are paraphyletic in some of our analyses (**S16 Fig**) but monophyletic in others (**S17 Fig**, **S18 Fig**). Of all these viruses, only one virus, termed Hubei myriapoda virus 6*,* has more than one genome segment and compatible segment termini. None of the viruses have the three segments that would be expected in hantaviruses and related bunyaviruses. Our data contribute the first full L-gene sequence in the subclade that is most closely related to hantaviruses (Coleopteran hanta-related virus OKIAV221). Comparison of genome termini identifies only minimal agreement with genome termini of hantaviruses. This makes it likely that these viruses can be classified in a new genus, once the first full genomes with overlapping segment ends become available.

**Novel group in the order *Bunyavirales***

A diversified clade of bunyaviruses branches as outgroup to members of *Hantavirus* and *Hantavirus*-related lineages, containing viruses associated with basal insect sequences and insect outgroups (Plecoptera, Odonata, Diplura, Campodea, and Myriapoda, **S16 Fig**). Some algorithms place it in sister relationship to *Peribunyaviridae*. Given the phylogenetic position of this monophyletic clade, and its distance to the neighbouring clades, it could be considered a new genus in the family *Peribunyaviridae* or a new family within the order *Bunyavirales* (**Table 1**). The lengths of Myriapodan hanta-related virus OKIAV214 and Plecopteran hanta-related virus OKIAV215 suggest detection of a full viral L-segment. An M-segment was detected for either Dipluran hanta-related virus OKIAV217 or -218. However, as these sequences stem from the same insect, it is unclear to which L-segment this M-segment belongs. The possibility of double infection with different viruses is another reason why segmented virus sequences should not be ascribed to genomes without experimental proof of viral replication.

The two recently classified genera, *Actinovirus* and *Agnathovirus*, found in fish, were not represented in any insect transcriptome in the present study [16, 23].

***Cruliviridae***

A solitary virus genome from a mix of crustaceans has been the basis for the designation of a novel viral family, *Cruliviridae* [3, 24]. The genome termini of the type species, *Crustacean lincruvirus* (Wenling crustacean virus 9), are virtually identical to those of the genus *Emaravirus* (family *Fimoviridae*). This suggests that bunyavirus genome termini can be conserved even across families (AGUAGA(A/G)), therefore this is an insufficient criterion for the definitive delineation of putatively novel genera [25, 26]. No cruliviruses are found in our large sample of insects.

***Peribunyaviridae***

The family *Peribunyaviridae* contains the genera *Orthobunyavirus*, *Herbevirus*, and *Shangavirus* (see below). A recently-added genus *Pacuvirus* is very closely related to *Orthobunyavirus* [16]. Orthobunyaviruses often infect vertebrates and are transmitted by mosquitoes, midges, phlebotomines, and ticks. Despite the comprehensive sample of insects tested, the present study identifies only two viruses found in Diptera (not included in **S5 Fig**), and thus does not add any new hosts, in terms of insect orders, for these viruses. Pacuviruses were also absent. The genus *Herbevirus* is a sister group to *Orthobunyavirus.* Herbeviruses have been fully characterized in cell culture, and their replication properties suggest a restriction to invertebrates [27]. The present data add novel sequences that clearly belong to *Herbevirus* and are derived from hosts as distant as lacewings and bark lice. This suggests that the dipteran-associated orthobunyaviruses may have evolved from a wider peribunyaviral diversity distributed in precursors to a range of insect orders. According to the present data, this range includes two groups basal to Diptera: Psocodea and Neuroptera. In view of the absence of herbe-/orthobunyaviruses in other major groups of insects it should be considered that tick-associated orthobunyaviruses may have been secondarily acquired from viremic vertebrate hosts.

It is noteworthy that Khurdun virus, isolated in Russia from coots at high prevalence and suggested to be an orthobunyavirus [28, 29], reliably groups with herbeviruses. The genome termini do not provide additional information for classification, as they are highly similar across orthobunya- and herbeviruses, including Khurdun virus. According to the present analysis, Khurdun virus is closer to herbeviruses, and in particular Psocodean peribunya-related virus OKIAV212 and Neuropteran peribunya-related virus OKIAV213, than to orthobunyaviruses [28]. Potential vertebrate tropism of Psocodean peribunya-related virus OKIAV212 and Neuropteran peribunya-related virus OKIAV213, or related viruses should be studied. Khurdun virus may in fact belong to herbeviruses, which would then suggest that herbeviruses may be able to infect vertebrates.

A recent ICTV report has designated a novel genus named *Shangavirus*, based on the solitary genome sequence of *Insect shangavirus* [24]. The phylogenetic position of *Shangavirus* is ambiguous, as shown by RdRp-based phylogenies. Some analyses place it in sister relationship to *Herbevirus* including Khurdun virus (**S16 Fig**), while others place it in sister relationship to *Herbe-* and *Orthobunyavirus* (**S16 Fig**, **S17 Fig**, **S18 Fig**). It should be noted that full genome analysis, as shown in the latest ICTV taxonomy report (2017.012M, [24]), is difficult to utilize as the combination of genome segments has not been confirmed by a live virus isolate. Segments of orthobunya- and herbeviruses normally have identical or highly similar reverse complementary ends within a viral genome, but this is not the case for *Insect shangavirus*. The M-segment tree topology of *Shangavirus* is congruent with that of the L-segment while congruence with the S-segment sequence (N-gene) is not supported (**S19 Fig**). As the related *Herbevirus* is also congruent between L- and M-, but not between L- and N-genes, this suggests that reassortment in lineage precursors to *Herbe-* and *Shangavirus* may have been involved in initial speciation and separation of these genera.

**Relationships among *Hanta-, Peribunya-, Tospo-,* and *Fimoviridae***

Collembolan phasma-related virus OKIAV223 is a large sequence of 8154 nucleotides, extending beyond the L-gene-ORF, but its genome termini are not convincingly shown to induce a reverse complementary panhandle structure. Further research is necessary to define whether a novel bunyaviral family should be established based on this deeply-branching sequence. Based on conserved motifs, the RdRp of Collembolan phasma-related virus OKIAV223 clusters with members of *Phasmaviridae* and branches from close to the last common ancestor of all members of *Peribunyaviridae* and *Phasmaviridae* (**S20 Fig**). If used as an outgroup for the peribunyavirus tree (**S16 Fig**), this outgroup re-defines the internal structure of the tree, which is also confirmed by analysis in MrBayes (alternative topologies are shown in **S17 Fig** and **S18 Fig A**, **B**). Since tospoviruses and emaraviruses are plant-associated viruses, two independent transitions from plants into metazoa may then have to be considered as compared to the simpler scenario of *Peribunyaviridae*, but not *Hantaviridae*, having evolved from plant viruses (**S16 Fig** and **S20 Fig**).

***Phasmaviridae***

The type species of this family were initially discovered in transcriptome sequences of phantom midges [30], establishing the current genus *Orthophasmavirus*. The family *Phasmaviridae* has recently been extended to include the genera *Orthophasmavirus*, *Inshuvirus*, *Feravirus*, *Wuhivirus*, and *Jonvirus* [24]. *Feravirus* and *Jonvirus* were designated as separate families in 2016 [31], but this classification was revised in 2017 [24].

The isolation in cell culture of the type species of the genera *Feravirus* and *Jonvirus* (Ferak and Jonchet virus, respectively) is still the only virological proof of phasmaviruses as replicating viruses [32]. Evidence of the formation of 21 nt small RNAs, indicating infection, was found in-vivo for a phasmavirus-like sequence in Drosophila [33]. The replication and transcription strategy of phasmaviruses has been characterized [32]. Both *Jonchet jonvirus* and *Ferak feravirus* have complementary genome segment ends that are identical between L-, M-, and S-segments. This criterion is not fulfilled for any of the sequence-based viruses classified as shown in **S21 Fig**, except for *Kigluaik phantom orthophasmavirus*. Experimental proof of replication should be gathered, especially in putative viruses detected in pooled samples [2].

Our data confirm many findings described in [3], and add a number of novel viruses that enable proposals for genus classification based on distance and, often, host association. These clades are named in **S21 Fig.** Four of them can be considered for the definition of novel genera, based on distance and availability of three-segmented genomes (**Table 1**). Dipteran-associated phasmaviruses (DAP) are defined by Wuhan mosquito orthophasmavirus 1 as well as sequences from the present study. All DAP members show tree congruence between L- and N-gene sequences but unclear topological resolution for the M-segment (glycoprotein gene) sequences (**S21 Fig**, **S24 Fig**). Li *et al.* and Shi *et al.* have found the viral genomes in this clade to only consist of L- and S-segments, with no M-segment [2, 3]. However, our data contain members of the same clades that do have M-segments, which raises the question of whether viruses with only two segments actually exist. RNAseq studies can fail to identify the full set of genome segments.

Odonatan phasma-related virus OKIAV254 confirms the host association of two viruses described in [3], Hubei odonate virus 8 and -9 (summarized in **S21 Fig**), here named Odonate-associated phasmaviruses (OAP). Three segments are known. Coleopteran phasma-related virus OKIAV236 and -235 stem from beetles, suggesting a Coleoptera-associated phasmavirus clade (CAP, with 3 segments known and congruence between L- and N-segment trees detected, **S24 Fig**). Blattodean phasma-related virus OKIAV239 and -238 confirm the host association of Wuchang cockroach virus 1, defining the Blattodea-associated phasmavirus clade (BAP). We find a large clade of OKIAV Hymenoptera-associated phasmaviruses (HAP), whose only additional member is a virus found recently in Hymenoptera, Ganda bee virus [34]. All HAP are topologically congruent between L-, N-, and M-segment phylogenies (**S24 Fig**). The only exception is Hymenopteran phasma-related virus OKIAV227 whose glycoprotein placement suggests reassortment or false assignment due to coinfection. There are two clades with mixed host associations that are sufficiently phylogenetically separate and have three known genome segments, altogether justifying consideration as novel genera: MAP1 (mixed host-associated phasmavirus 1) and MAP2 (mixed host-associated phasmavirus 2). DAP2 is another dipteran-associated lineage defined by a solitary virus (Dipteran phasma-related virus OKIAV226) with a fully-sequenced three-partite genome, and may constitute a new genus (**S21 Fig**).

A novel sister clade to all members of the genus *Feravirus* is entirely defined by viruses from the present study. For two viruses, Hymenopteran phasma-related virus OKIAV252 and -250, complete L-segments are available. Hymenopteran phasma-related virus OKIAV252 has a matching S-segment sequence whose 5´-end aligns with that of the L-segment. We use the clade designation HAF for these Hymenoptera-associated *Feravirus*-related viruses. For almost all HAF, three genome segments have been identified, and one of them has complete (and identical) genome segment termini (Hymenopteran phasma-related virus OKIAV252). Classification of HAF as a genus may be formally possible on this basis. Of note, a very recent description of a partial L-segment of Notori virus suggests this virus may belong to HAF. However, in view of its evolutionary distance and association with Diptera instead of Hymenoptera, it may as well represent another genus. Also this virus cannot be classified because it lacks a full genome sequence.

Hemipteran phasma-related virus OKIAV247 and Neuropteran phasma-related virus OKIAV248 are novel feraviruses with complete genome sequences and reverse complementary genome termini. While feraviruses have only been found in mosquitoes (Diptera) so far, the novel viruses stem from a lacewing (Neuroptera) and a shieldbug (Hemiptera). Notably, whereas diversified clades around members of *Ferak feravirus* emerge, *Jonchet jonvirus* remains the sole member of a deep-branching lineage (genus *Jonvirus*). Considering that members of the species *Jonchet jonvirus* were repeatedly found in mosquitoes by our group, this suggests tight host restriction (no mosquitos are represented in the current dataset).

The recently classified genus *Wuhivirus* was identified in water striders and a mix of insects in China [2, 24]. We find viral sequences which group together with *Wuhivirus*, thus confirming the finding. The host associations of this clade are highly diverse since viral sequences are derived from members of two different families of Hemiptera, Lepidoptera, and mixes of water striders and Odonata. We did not find identical genome termini in either the known or the novel members of this tentative genus. In summary, 10 novel genera may be considered within the family *Phasmaviridae* based on the present data.

***Phenuiviridae***

The genus *Phlebovirus* in the newly defined family *Phenuiviridae* classifies some of the most medically-relevant bunyaviruses. Viruses belong to over 70 antigenically distinct serotypes and include several human and livestock pathogens, such as *Rift Valley fever phlebovirus*, and several sandfly fever viruses. Most phleboviruses are arboviruses, and most are mosquito-, sandfly-, or tick-borne.

A novel genus termed *Banyangvirus* has recently been proposed and accepted in the most recent ICTV taxonomy update (proposal 2017.012M, [24]). An additional proposal suggests the expansion of *Banyangvirus* by adding further viral taxa (proposal 2018.013M, [35]). However, our phylogenetic analysis finds that the members of *Banyangvirus* are in sister relationship to members of the classical genus *Phlebovirus*. As it appears, mammalian and dipteran members of *Phlebovirus* group together with *Banyangvirus*, to the exclusion of *Uukuniemi*- and other tick-associated phleboviruses. The existence of *Banyangvirus* makes *Phlebovirus* a paraphyletic genus (**S25 Fig**). This issue has not been addressed by the proposal 2017.012M, in which the re-organisation of the order *Bunyavirales* has been promoted. In this proposal, the phylogenetic inference includes only the type species of the genera, therefore overlooking the within-genus relationships and inevitably rendering *Phlebovirus* and *Banyangvirus* as sister clades.

The ICTV proposal 2018.013M is based on an analysis of nucleotide sequences, while amino acid sequence analysis is more robust against errors caused by substitution saturation. Indeed, the phylogenetic support for the L-gene analysis in 2018.013M is at the borderline of significance. The alignment of structural gene portions might be better resolved, but the topological incongruence implied by the overall phylogeny (in our and earlier analyses compared to 2018.013M) suggests reassortment within the phlebo- and banyangvirus stem lineages. Consequently, members of *Phlebovirus* and *Banyangvirus* seem to have undergone recent speciation and still carry footprints of genetic exchange. This argues against the classification of each as a separate genus, but rather for designating *Banyangvirus* as a subgenus of *Phlebovirus*. Additional supports for this ranking are the phylogenies derived from the nucleoprotein and glycoprotein of phlebo- and banyangviruses, which show a very similar topology (**S28 Fig**).

Interestingly, we do not find any novel insect-associated phleboviruses. This is remarkable as the studies of [2] and [3] also did not reveal any novel phleboviruses in insects, but did find phleboviruses in ticks. Odonatan phenui-related virus OKIAV258 and Hymenopteran phenui-related virus OKIAV306 fall between phlebo- and tenuiviruses, together with several sequence findings from [2] and [3]. However, none of the genomes of these viruses is fully sequenced.

Two newly classified genera, *Hudovirus* and *Pidchovirus,* have been defined in the family *Phenuiviridae*. The genome of *Lepidopteran hudovirus*, type species of *Hudovirus*, has 3 segments whose ends are reverse-complementary and identical among all 3 segments. The genome of *Pidgey pidchovirus*, type species of *Pidchovirus*, has segment termini that are neither reverse-complementary nor identical for any segment. We find fragments of viral sequences for all L-, M-, and S-segments, but none of them have known genome termini. The sequences of *Hudo*- and *Pidchovirus* were both found in Lepidoptera, unlike our findings which are all found in various insect orders.

Four additional novel genera have been assigned by ICTV to *Phenuiviridae*: *Phasi-*, *Hudi-*, *Beidi-*, and *Wubeivirus*. We do not find viral sequences that cluster with any of those genera. The genomes of *Hudi-*, *Beidi-*, and *Wubeivirus* do not have complementary genome segment termini and were initially found in mixed samples of Diptera (**S25 Fig**). Of note, the two viruses assigned to *Wubeivirus* appear paraphyletic in all our analysis, which argues against their classification in the same genus. Phylogenetic analyses in now-accepted classification proposals includes M- and S-segment sequences. However, the use of concatenated alignments in this approach ignores the possibility that the segments evolved as independent genomic units. Our analysis of segment co-segregation does not find topological congruence between L-, M-, and S-segments in these viruses (**S28 Fig**). With the exception of *Badu phasivirus*, none of the sequences pertaining to the members of these genera have complementary genome segment termini. In addition, for all members, including *Badu phasivirus,* the genome termini among segments are not identical. Because most of the sequences were detected in mixed samples, it is possible that a mixture of segments from different viruses was isolated. Therefore, none of these sequences should be regarded as concrete viral genomes.

The genus *Goukovirus* is named after *Gouleako goukovirus*, a fully-characterized virus based on cell culture isolates [36]. The genome termini of *Gouleako goukovirus* are reverse-complementary, over ~14nt, and identical among S-, M-, and L-segments. Host associations of the viral sequences from the present study suggest a predominant, but not exclusive, representation in Hymenoptera while the type isolates stem from mosquitoes (Diptera).

Coleopteran phenui-related virus OKIAV293 represents a novel lineage branching as deep as typical for a novel genus but cannot be classified as such because it does not constitute a coding-complete genome.

At the bottom of the phylogenetic tree in **S25 Fig**, a large clade within the family *Phenuiviridae* contains 10 sequences from [2] and [3], *Mothra mobuvirus*, and 26 novel sequences from the present study. *Mothra mobuvirus* is the type species of the novel genus *Mobuvirus* and has been detected in moths [4]. The phylogenetic diversity and the genetic distance of this large monophyletic group suggest the definition of several genera that may then form a subfamily within *Phenuiviridae*. The host associations of this clade’s viruses are diverse, spanning 10 different insect orders. Among all of these viruses, Hemipteran phenui-related virus OKIAV285 and Blattodean phenui-related virus OKIAV266 are the only sequences with complementary L-segment genome termini. Each of them is found in one of two subclades of the new monophyletic group, differing considerably from each other and from *Goukovirus* and *Phlebovirus*. Across all members of this large clade, the number of genome segments ranges from one to three. Wherever two segments are found, those represent the L- and M-, or L- and S-segments. This points to the presence of three segments in all viruses. Given the tremendous evolutionary distance between S- and M-segments among bunyaviruses, it is likely that virus identification based on RNAseq data will not correctly, if at all, detect missing diverse segments. Of note, *Mothra mobuvirus* has only two segments and should thus be subjected to experimental confirmation of replication to confirm this unusual genome architecture.

***Arenaviridae***

The family *Arenaviridae* consists of the genera *Mammarenavirus*, *Reptarenavirus*, *Antennavirus*, and *Hartmanivirus*. The genus *Mammarenavirus* includes important human pathogens such as *Lassa virus* and the highly pathogenic New World arenaviruses. All species in the genera *Reptarenavirus* and *Hartmanivirus* occur only in snakes. As shown in **S30 Fig**, Dipteran mypo-related virus OKIAV322, found in australian long-legged flies (*Heteropsilopus ingenuus*), groups together with *Myriapod hubavirus*. The latter is the sole member of the newly assigned genus *Hubavirus* and family *Mypoviridae*. The phylogenetic distance in this newly-formed clade matches the intra-genus distance of *Mammarenavirus*, thus supporting the assignment of *Hubavirus*.

***Nairoviridae***

The family *Nairoviridae* includes tick-transmitted arboviruses such as the *Crimean-Congo hemorrhagic fever orthonairovirus*, classified in the genus *Orthonairovirus*. Arthropod viruses related to this genus are almost exclusively found in ticks [2, 3]. In the most recent classification proposal (ICTV proposal 2018.017M, [23]), the sequence of *Strider striwavirus* found in a mix of water striders, is assigned to the monospecific genus *Striwavirus*. Our data confirm the existence of this clade by contributing another insect-associated viral sequence, Blattodean nairo-related virus OKIAV321, which groups together with *Strider striwavirus*. Blattodean nairo-related virus OKIAV321 has three genome segments, like *Strider striwavirus*, which are however incomplete, and do not allow for genome termini examination. Our finding does not stem from a water strider, but from the cockroach species *Paratemnopterix couloniana* that is widely used by pet keepers as a food source for scorpions, spiders, and other insect-fed companion animals. The clustering of the unclassified Xinzhou spider virus within this clade suggests that this virus may fall into this tentative genus.

The absence of members of the genus *Orthonairovirus* in our vast sample of insects suggests that orthonairoviruses may be exclusively associated with ticks, while the source of nairovirus diversity spans a wide invertebrate host range.

Viral sequences found in myriapods in the present study, as well as in crabs from [3], group together into a highly distinct sister clade to arena- and nairoviruses (**S30 Fig**). While Jiangxia mosquito virus 1 could be considered a type species with two known segments, none of the members of this clade have been formally assigned to a novel genus or family. Its phylogenetic distance is similar to that of other families in the tree, suggesting that it may define a novel family.

Overall, the co-segregation of segments shows some topologically congruent clades that are larger compared to the other phylogenies in this study (**S33 Fig**). This might be explained by the lack of novel sequences and the appropriate annotation and characterization of the viruses backed by laboratory experiments.

**Results of cytochrome oxidase subunit 1 (COI) barcode analysis**

We conducted searches for contaminating non-hexapod sequences in all transcriptome assemblies used in the study. The search was based on ~2.5M Eukaryote cytochrome c oxidase subunit 1 gene sequences from GenBank and ~0.5M additional gene barcodes from the German Barcode of Life project. All findings are summarized in **Supporting Information S3** and **S4 Tables**. Four OKIAV viruses assembled from transcriptomes in which non-hexapod sequences were detected are included in phylogenies of **Fig 1**, marked with an empty triangle. Based on the distribution of contaminated assemblies matching non-hexapod organisms, we regard the possibility of having detected a clade of viruses that actually belongs to contaminant organisms instead of hexapods as very unlikely.

## References

1. Contreras MA, Eastwood G, Guzman H, Popov V, Savit C, Uribe S, et al. Almendravirus: A Proposed New Genus of Rhabdoviruses Isolated from Mosquitoes in Tropical Regions of the Americas. Am J Trop Med Hyg. 2017;96(1):100-9. doi: 10.4269/ajtmh.16-0403.

2. Li C-X, Shi M, Tian J-H, Lin X-D, Kang Y-J, Chen L-J, et al. Unprecedented genomic diversity of RNA viruses in arthropods reveals the ancestry of negative-sense RNA viruses. Elife. 2015;4. doi: 10.7554/eLife.05378.

3. Shi M, Lin XD, Tian JH, Chen LJ, Chen X, Li CX, et al. Redefining the invertebrate RNA virosphere. Nature. 2016. Epub 2016/11/24. doi: 10.1038/nature20167.

4. Makhsous N, Shean RC, Droppers D, Guan J, Jerome KR, Greninger AL. Genome Sequences of Three Novel Bunyaviruses, Two Novel Rhabdoviruses, and One Novel Nyamivirus from Washington State Moths. Genome Announc. 2017;5(7). doi: 10.1128/genomeA.01668-16.

5. Maes P, Song T, Mark S, Paweska J, Song Q, Ye G, et al. ICTV taxonomic report 2017.016M.R.: Taxonomic expansion and reorganization of the order Mononegavirales.

6. Kuhn JH, Bekal S, Caì Y, Clawson AN, Domier LL, Herrel M, et al. Nyamiviridae: proposal for a new family in the order Mononegavirales. Arch Virol. 2013;158(10):2209-26. doi: 10.1007/s00705-013-1674-y.

7. Mihindukulasuriya KA, Nguyen NL, Wu G, Huang HV, da Rosa APAT, Popov VL, et al. Nyamanini and midway viruses define a novel taxon of RNA viruses in the order Mononegavirales. J Virol. 2009;83(10):5109-16. doi: 10.1128/JVI.02667-08.

8. Rogers MB, Cui L, Fitch A, Popov V, Travassos da Rosa APA, Vasilakis N, et al. Whole genome analysis of sierra nevada virus, a novel mononegavirus in the family nyamiviridae. Am J Trop Med Hyg. 2014;91(1):159-64. doi: 10.4269/ajtmh.14-0076.

9. Bekal S, Domier LL, Niblack TL, Lambert KN. Discovery and initial analysis of novel viral genomes in the soybean cyst nematode. J Gen Virol. 2011;92(Pt 8):1870-9. doi: 10.1099/vir.0.030585-0.

10. Kleanthous E, Olendraite I, Lukhovitskaya NI, Firth AE. Discovery of three RNA viruses using ant transcriptomic datasets. Arch Virol. 2019;164(2):643-7. doi: 10.1007/s00705-018-4093-2.

11. Wang F, Fang Q, Wang B, Yan Z, Hong J, Bao Y, et al. A novel negative-stranded RNA virus mediates sex ratio in its parasitoid host. PLoS Pathog. 2017;13(3):e1006201. doi: 10.1371/journal.ppat.1006201.

12. Kuhn JH, Dietzgen RG, Easton AJ, Kurath G, Nowotny N, Rima BK, et al. ICTV taxonomic proposal 2015.010A: Five (5) new unassigned genera in the order Mononegavirales.

13. Viljakainen L, Holmberg I, Abril S, Jurvansuu J. Viruses of invasive Argentine ants from the European Main supercolony: characterization, interactions and evolution. J Gen Virol. 2018;99(8):1129-40. doi: 10.1099/jgv.0.001104.

14. Liu L, Xie J, Cheng J, Fu Y, Li G, Yi X, et al. Fungal negative-stranded RNA virus that is related to bornaviruses and nyaviruses. Proc Natl Acad Sci U S A. 2014;111(33):12205-10. doi: 10.1073/pnas.1401786111.

15. Drexler JF, Corman VM, Müller MA, Maganga GD, Vallo P, Binger T, et al. Bats host major mammalian paramyxoviruses. Nat Commun. 2012;3:796. doi: 10.1038/ncomms1796.

16. Shi M, Lin X-D, Chen X, Tian J-H, Chen L-J, Li K, et al. The evolutionary history of vertebrate RNA viruses. Nature. 2018;556(7700):197-202. doi: 10.1038/s41586-018-0012-7.

17. Presti RM, Zhao G, Beatty WL, Mihindukulasuriya KA, da Rosa APAT, Popov VL, et al. Quaranfil, Johnston Atoll, and Lake Chad viruses are novel members of the family Orthomyxoviridae. J Virol. 2009;83(22):11599-606. doi: 10.1128/JVI.00677-09.

18. Allison AB, Ballard JR, Tesh RB, Brown JD, Ruder MG, Keel MK, et al. Cyclic avian mass mortality in the northeastern United States is associated with a novel orthomyxovirus. J Virol. 2015;89(2):1389-403. doi: 10.1128/JVI.02019-14.

19. Dannevig BH, Falk K, Namork E. Isolation of the causal virus of infectious salmon anaemia (ISA) in a long-term cell line from Atlantic salmon head kidney. J Gen Virol. 1995;76 ( Pt 6):1353-9. doi: 10.1099/0022-1317-76-6-1353.

20. Jones LD, Matthewson M, Nuttall PA. Saliva-activated transmission (SAT) of Thogoto virus: dynamics of SAT factor activity in the salivary glands of Rhipicephalus appendiculatus, Amblyomma variegatum, and Boophilus microplus ticks. Exp Appl Acarol. 1992;13(4):241-8. doi: 10.1007/BF01195081

21. Eyngor M, Zamostiano R, Kembou Tsofack JE, Berkowitz A, Bercovier H, Tinman S, et al. Identification of a novel RNA virus lethal to tilapia. J Clin Microbiol. 2014;52(12):4137-46. doi: 10.1128/JCM.00827-14.

22. Contreras-Gutiérrez MA, Nunes MRT, Guzman H, Uribe S, Suaza Vasco JD, Cardoso JF, et al. Sinu virus, a novel and divergent orthomyxovirus related to members of the genus Thogotovirus isolated from mosquitoes in Colombia. Virology. 2017;501:166-75. doi: 10.1016/j.virol.2016.11.014.

23. Maes P, Kuhn JH. ICTV taxonomic proposal 2018.017M: Expansion of the order Bunyavirales.

24. Maes P, Alkhovsky S, Beer M, Briese T, Buchmeier MJ, Calisher CH, et al. ICTV taxonomic proposal 2017.012M: Taxonomic expansion and reorganization of the order Bunyavirales.

25. Elliott RM. Molecular biology of the Bunyaviridae. J Gen Virol. 1990;71 ( Pt 3):501-22. doi: 10.1099/0022-1317-71-3-501.

26. Mir MA, Brown B, Hjelle B, Duran WA, Panganiban AT. Hantavirus N protein exhibits genus-specific recognition of the viral RNA panhandle. J Virol. 2006;80(22):11283-92. doi: 10.1128/JVI.00820-06.

27. Marklewitz M, Zirkel F, Rwego IB, Heidemann H, Trippner P, Kurth A, et al. Discovery of a unique novel clade of mosquito-associated bunyaviruses. J Virol. 2013;87(23):12850-65. doi: 10.1128/JVI.01862-13.

28. Al'kovskhovskiĭ SV, Shchetinin AM, L'Vov DK, Shchelkanov MI, Deriabin PG, L'Vov DN, et al. The Khurdun virus (KHURV): a new representative of the orthobunyavirus (Bunyaviridae). Voprosy virusologii. 2013;58(4):10-3.

29. Galkina IV, L'Vov LN, Gromashevskiĭ VL, others. Khurdun virus, a presumably new RNA-containing virus associated with coots (Fulica atra), isolated in the Volga river delta. Voprosy virusologii. 2005;50(4):29-31.

30. Ballinger MJ, Bruenn JA, Hay J, Czechowski D, Taylor DJ. Discovery and evolution of bunyavirids in arctic phantom midges and ancient bunyavirid-like sequences in insect genomes. J Virol. 2014;88(16):8783-94. doi: 10.1128/JVI.00531-14.

31. Briese T, Alkhovsky S, Beer M, Calisher CH, Charrel R, Ebihara H, et al. ICTV taxonomic proposal 2016.030a-vM: Create a new order, Bunyavirales, to accommodate nine families (eight new, one renamed) comprising thirteen genera.

32. Marklewitz M, Zirkel F, Kurth A, Drosten C, Junglen S. Evolutionary and phenotypic analysis of live virus isolates suggests arthropod origin of a pathogenic RNA virus family. Proc Natl Acad Sci U S A. 2015;112(24):7536-41. doi: 10.1073/pnas.1502036112.

33. Webster CL, Waldron FM, Robertson S, Crowson D, Ferrari G, Quintana JF, et al. The discovery, distribution, and evolution of viruses associated with Drosophila melanogaster. PLoS biology. 2015;13(7):e1002210.

34. Schoonvaere K, De Smet L, Smagghe G, Vierstraete A, Braeckman BP, de Graaf DC. Unbiased RNA Shotgun Metagenomics in Social and Solitary Wild Bees Detects Associations with Eukaryote Parasites and New Viruses. PLoS One. 2016;11(12):e0168456. doi: 10.1371/journal.pone.0168456.

35. Shen S, Duan X, Wang B, Zhu L, Zhang Y, Zhang J, et al. ICTV taxonomic proposal 2018.013M: One (1) new species in the genus Banyangvirus (Bunyavirales: Phenuiviridae).

36. Marklewitz M, Handrick S, Grasse W, Kurth A, Lukashev A, Drosten C, et al. Gouleako virus isolated from West African mosquitoes constitutes a proposed novel genus in the family Bunyaviridae. J Virol. 2011;85(17):9227-34. doi: 10.1128/JVI.00230-11.

**References of supporting information legends**

1. Nieva, J. L., & Carrasco, L. Viroporins: Structures and functions beyond cell membrane permeabilization. Viruses. 2015;7(10): 5169–5171. doi: 10.3390/v7102866.
