# Supplementary figures and images for: Re-assessing the diversity of negative strand RNA viruses in insects
Source: PLoS Pathog. 2019 Dec 12;15(12):e1008224. doi: 10.1371/journal.ppat.1008224 (PMC6932829; doi:10.1371/journal.ppat.1008224)

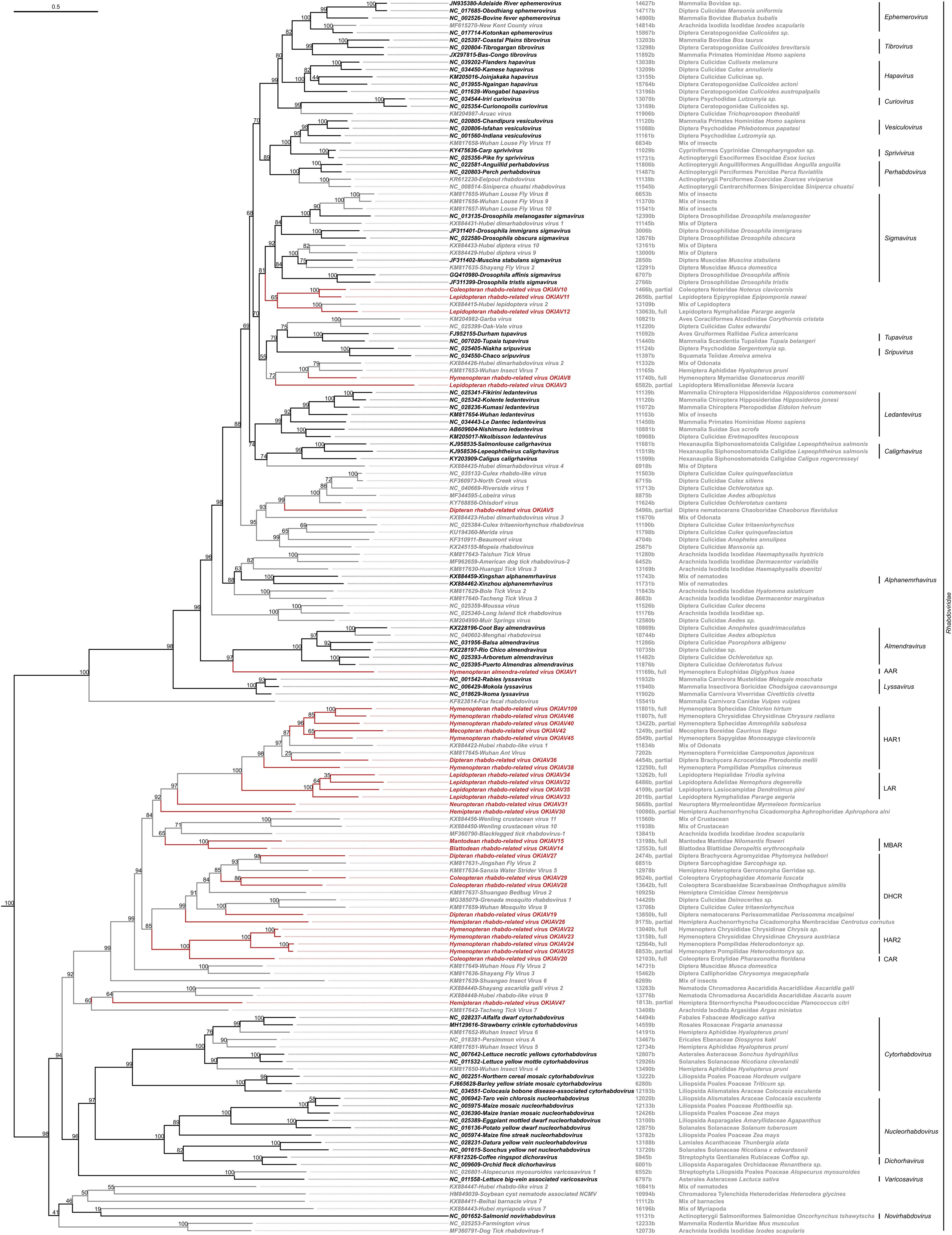

Supplement: S1 Fig — Maximum likelihood phylogenies based on RAxML. Black branches show ICTV-accepted taxa, grey branches show unclassified taxa, and red branches show OKIAVs. Columns on the right summarize contig length, genome completeness, taxonomic grouping of hosts, and viral genus and family. The outgroup taxon (not shown) is Mammalian 1 orthobornavirus (Bornaviridae). Analyses based on PhyML and MrBayes can be found in S2 and S3 Figs. (TIF) [file ppat.1008224.s006.tif]

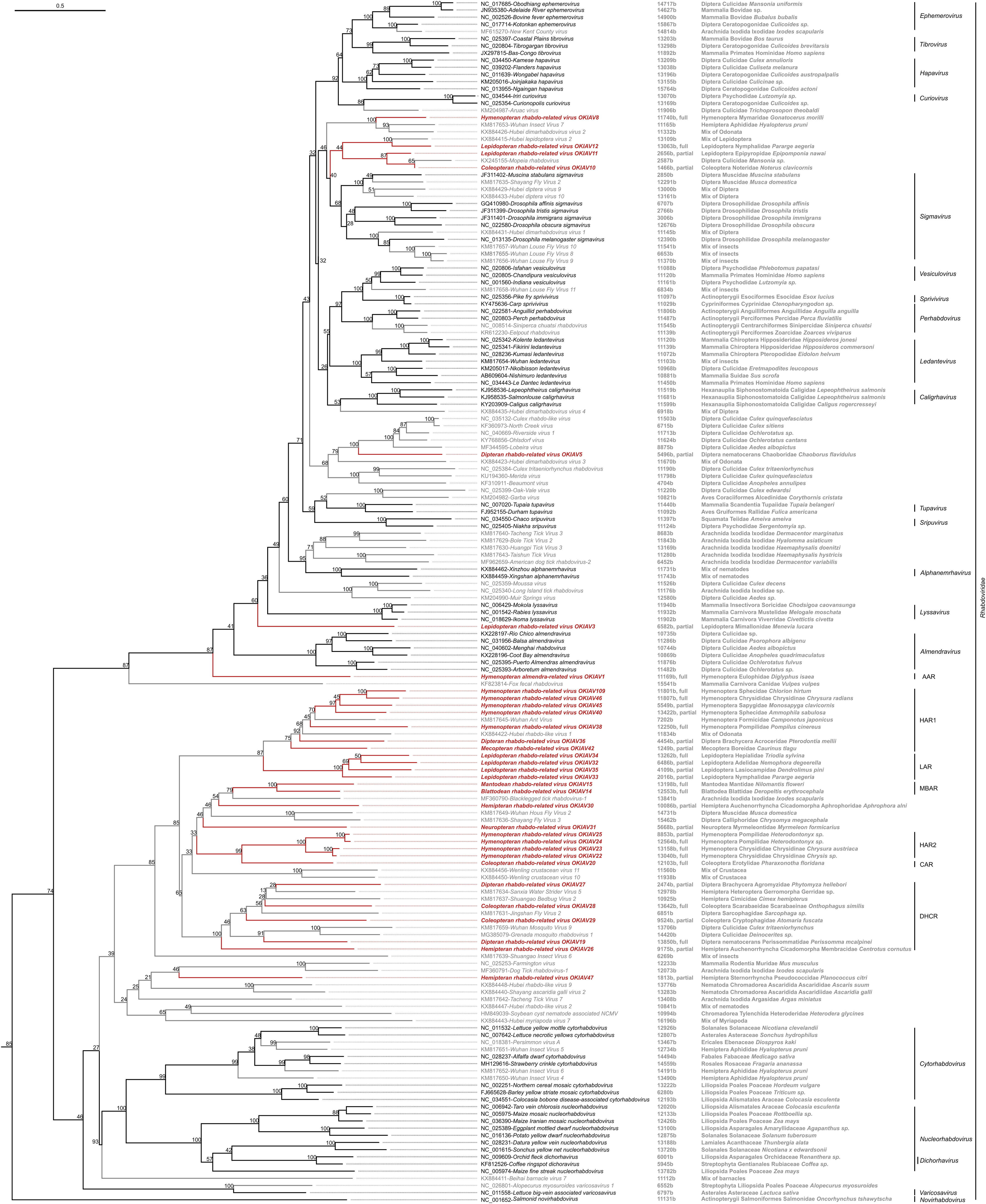

Supplement: S2 Fig — Black branches show ICTV-accepted taxa, grey branches show unclassified taxa, and red branches show OKIAVs. Columns on the right side summarize contig length, genome completeness, taxonomic grouping of hosts, and viral genus and family. The outgroup taxon not shown) is Mammalian 1 orthobornavirus (Bornaviridae). (TIF) [file ppat.1008224.s007.tif]

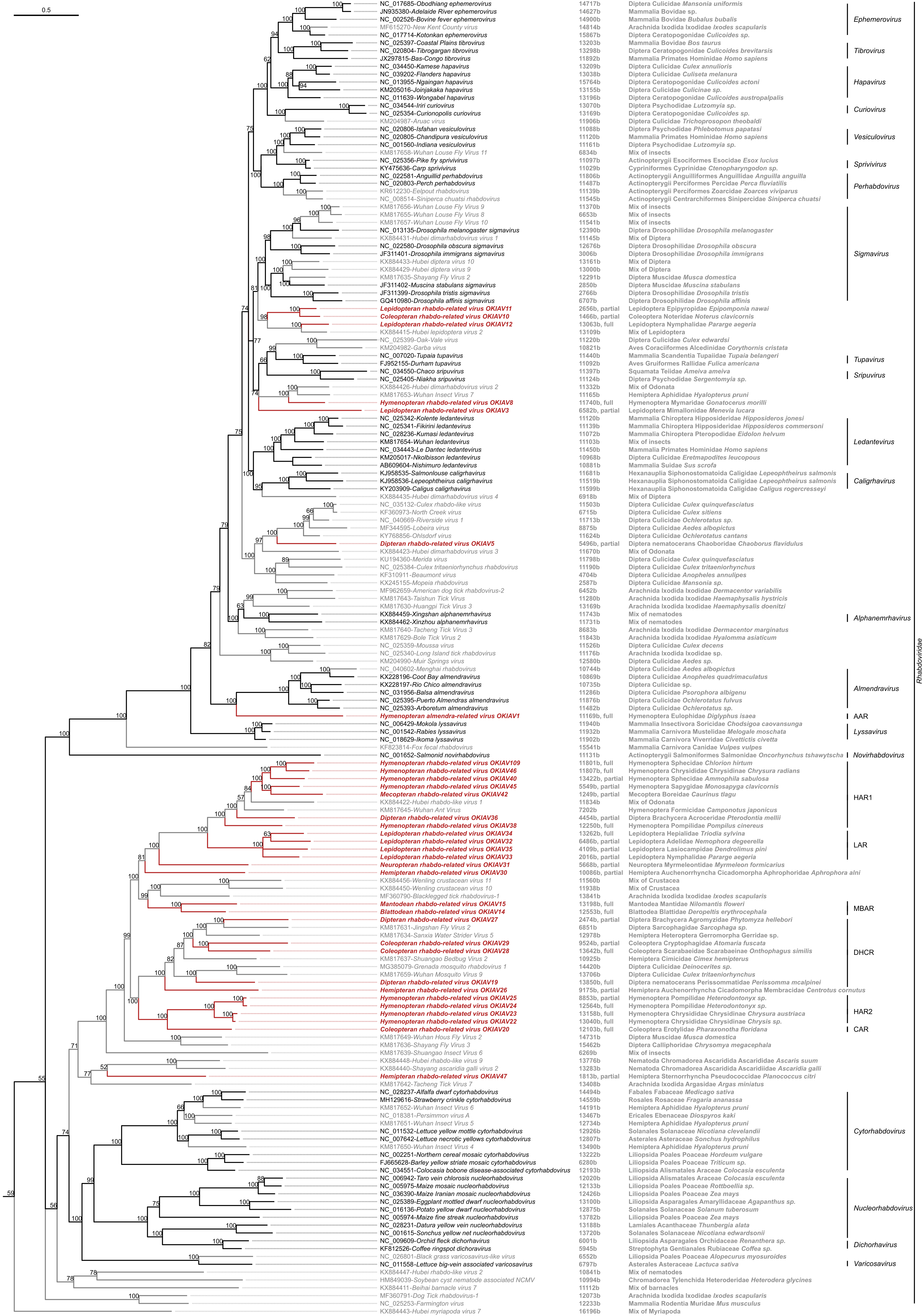

Supplement: S3 Fig — Black branches show ICTV-accepted taxa, grey branches show unclassified taxa, and red branches show OKIAVs. Columns on the right side summarize contig length, genome completeness, taxonomic grouping of hosts, and viral genus and family. The outgroup taxon not shown) is Mammalian 1 orthobornavirus (Bornaviridae). (TIF) [file ppat.1008224.s008.tif]

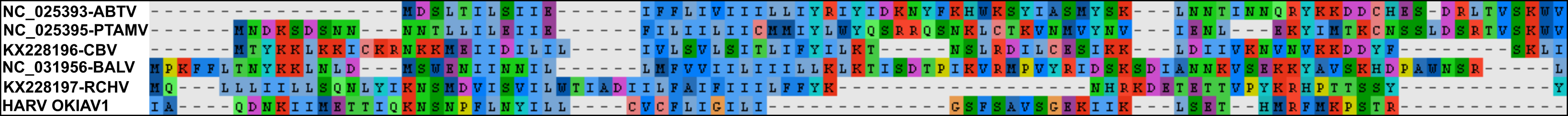

Supplement: S4 Fig — Reference sequences belong to Arboretum almendravirus (ABTV), Puerto Almendras almendravirus (PTAMV), Coot Bay almendravirus (CBV), Balsa almendravirus (BALV), and Rio Chico almendravirus (RCHV). The hydrophobic stretches with multiple leucins (L) and isoleucins (I) interact with the cell membrane to facilitate cell entry [1]. (TIF) [file ppat.1008224.s009.tif]

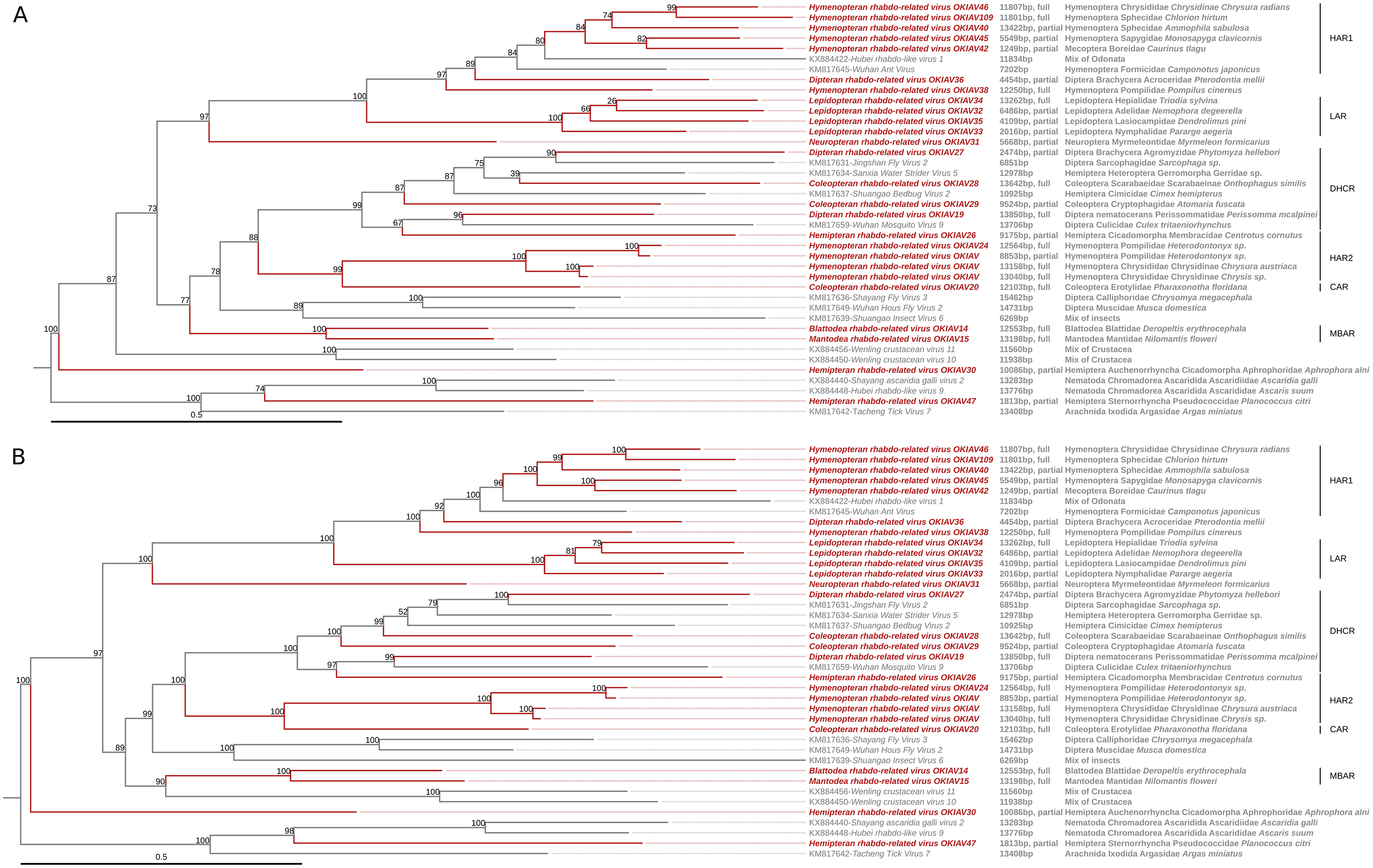

Supplement: S5 Fig — Maximum likelihood phylogenies based on RaxML (A) and Bayesian inference of phylogeny with MrBayes (B). Grey branches show unclassified taxa, and red branches show OKIAVs. Columns on the right side summarize contig length, genome completeness, and taxonomic grouping of hosts. (TIF) [file ppat.1008224.s010.tif]

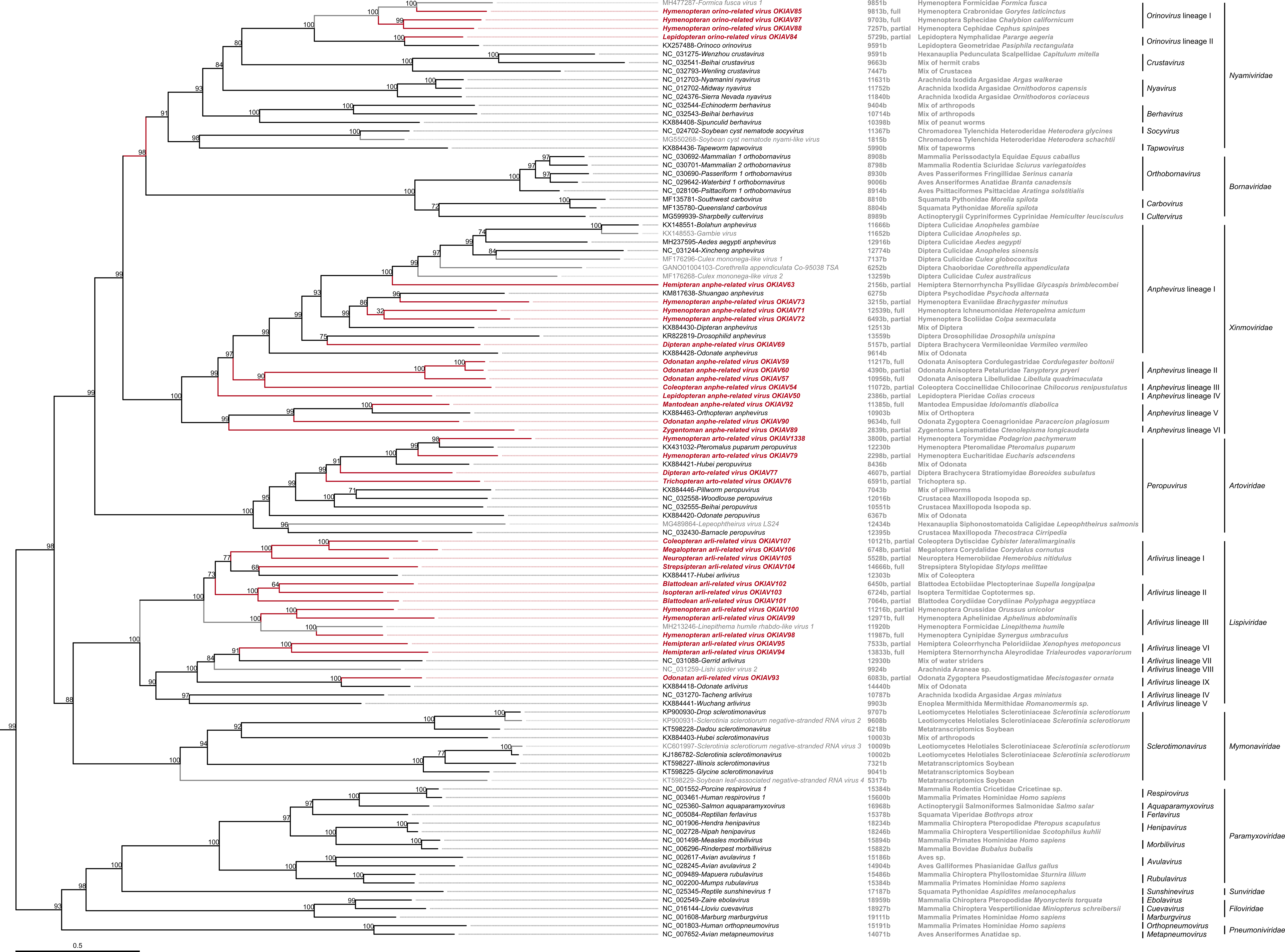

Supplement: S6 Fig — Maximum likelihood phylogenies based on RAxML. Black branches show ICTV-accepted taxa, grey branches show unclassified taxa, and red branches show OKIAVs. Columns on the right summarize contig length, genome completeness, taxonomic grouping of hosts, and viral genus and family. The outgroup taxon (not shown) is Salmonid rhabdovirus (Rhabdoviridae). Analyses based on PhyML and MrBayes can be found in S7 and S8 Figs. (TIF) [file ppat.1008224.s011.tif]

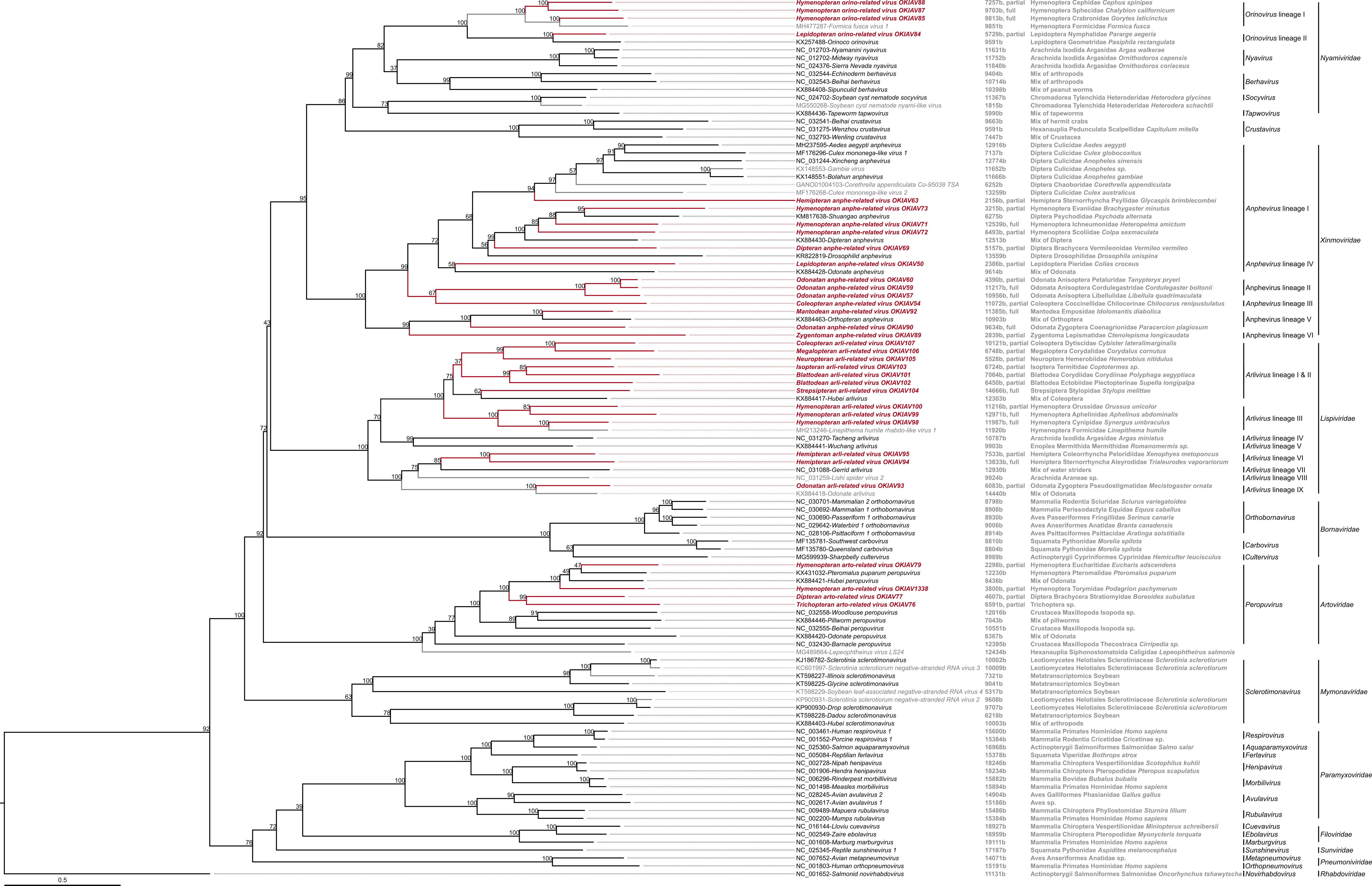

Supplement: S7 Fig — Black branches show ICTV-accepted taxa, grey branches show unclassified taxa, and red branches show OKIAVs. Columns on the right side summarize contig length, genome completeness, taxonomic grouping of hosts, and viral genus and family. The outgroup taxon (not shown) is Salmonid rhabdovirus (Rhabdoviridae). (TIF) [file ppat.1008224.s012.tif]

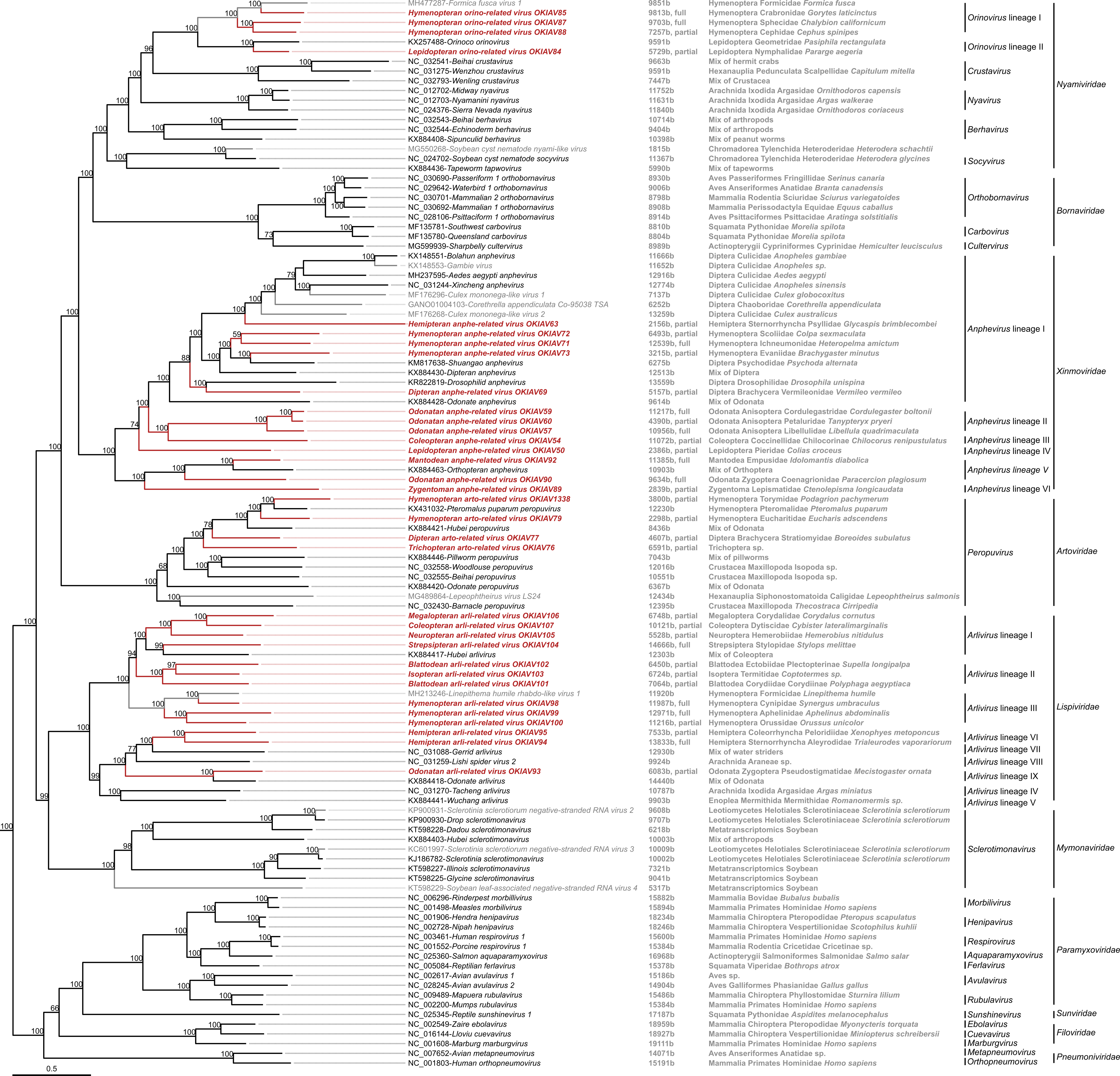

Supplement: S8 Fig — Black branches show ICTV-accepted taxa, grey branches show unclassified taxa, and red branches show OKIAVs. Columns on the right side summarize contig length, genome completeness, taxonomic grouping of hosts, and viral genus and family. The outgroup taxon (not shown) is Salmonid rhabdovirus (Rhabdoviridae). (TIF) [file ppat.1008224.s013.tif]

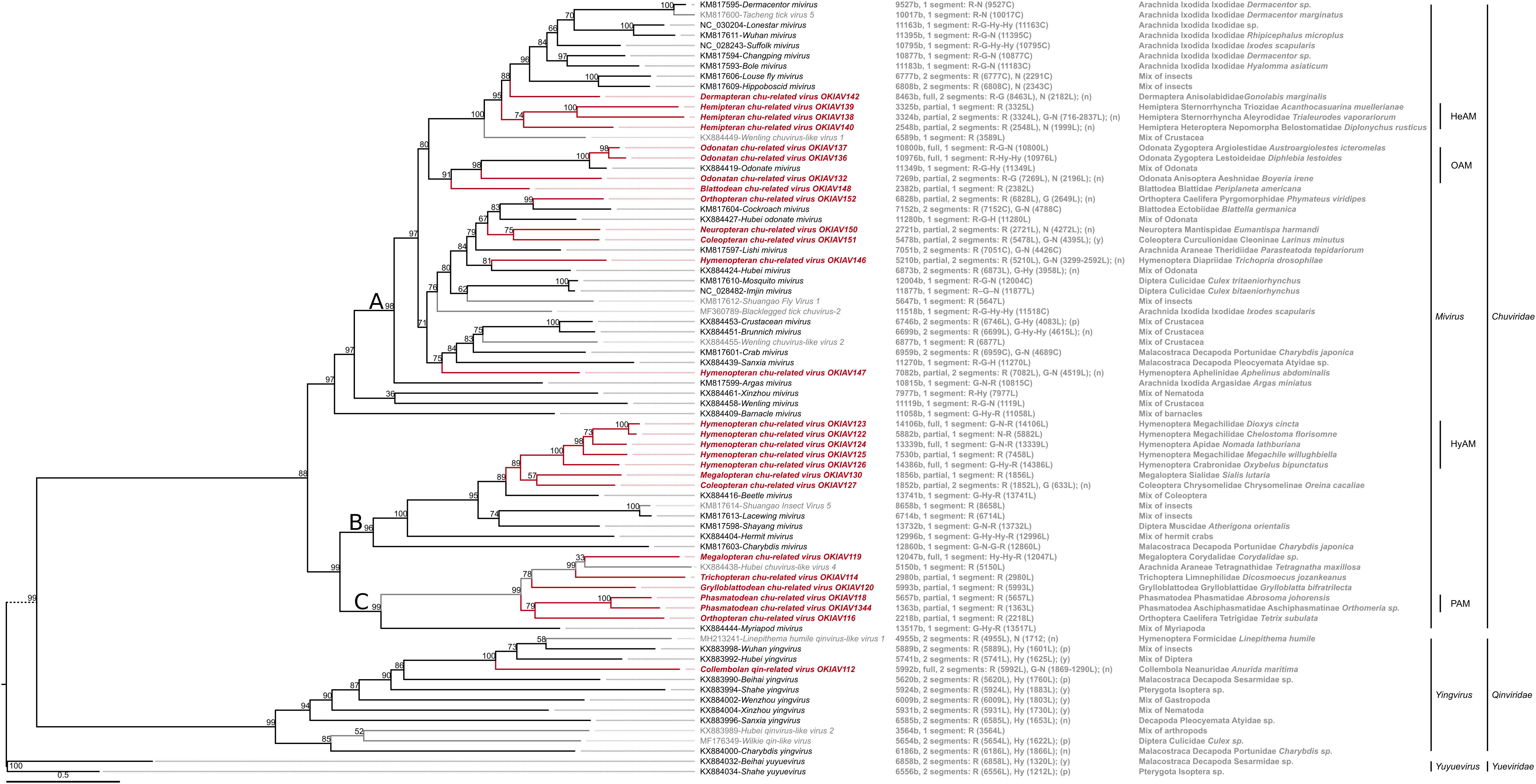

Supplement: S9 Fig — Maximum likelihood phylogenies based on RAxML. Black branches show ICTV-accepted taxa, grey branches show unclassified taxa, and red branches show OKIAVs. Columns on the right summarize contig length, genome completeness, number of segments, taxonomic grouping of hosts, and viral genus and family. Genomic protein-coding regions are: R = RdRp, G = glycoprotein, N = nucleoprotein, Hy = hypothetical protein with unknown function. Segment length and organization are shown in parentheses: linear (L) or circular (C). For linear segments, information on the segment ends is given as: (n) = segment ends not matching the ends of the RdRp segment, (y) = segment ends matching the ends of the RdRp segment, (p) = segment ends partially matching the ends of the RdRp segment. The tree is rooted to Yuevirus (Yueviridae). Analyses based on PhyML and MrBayes can be found in S10 and S11 Figs. (TIF) [file ppat.1008224.s014.tif]

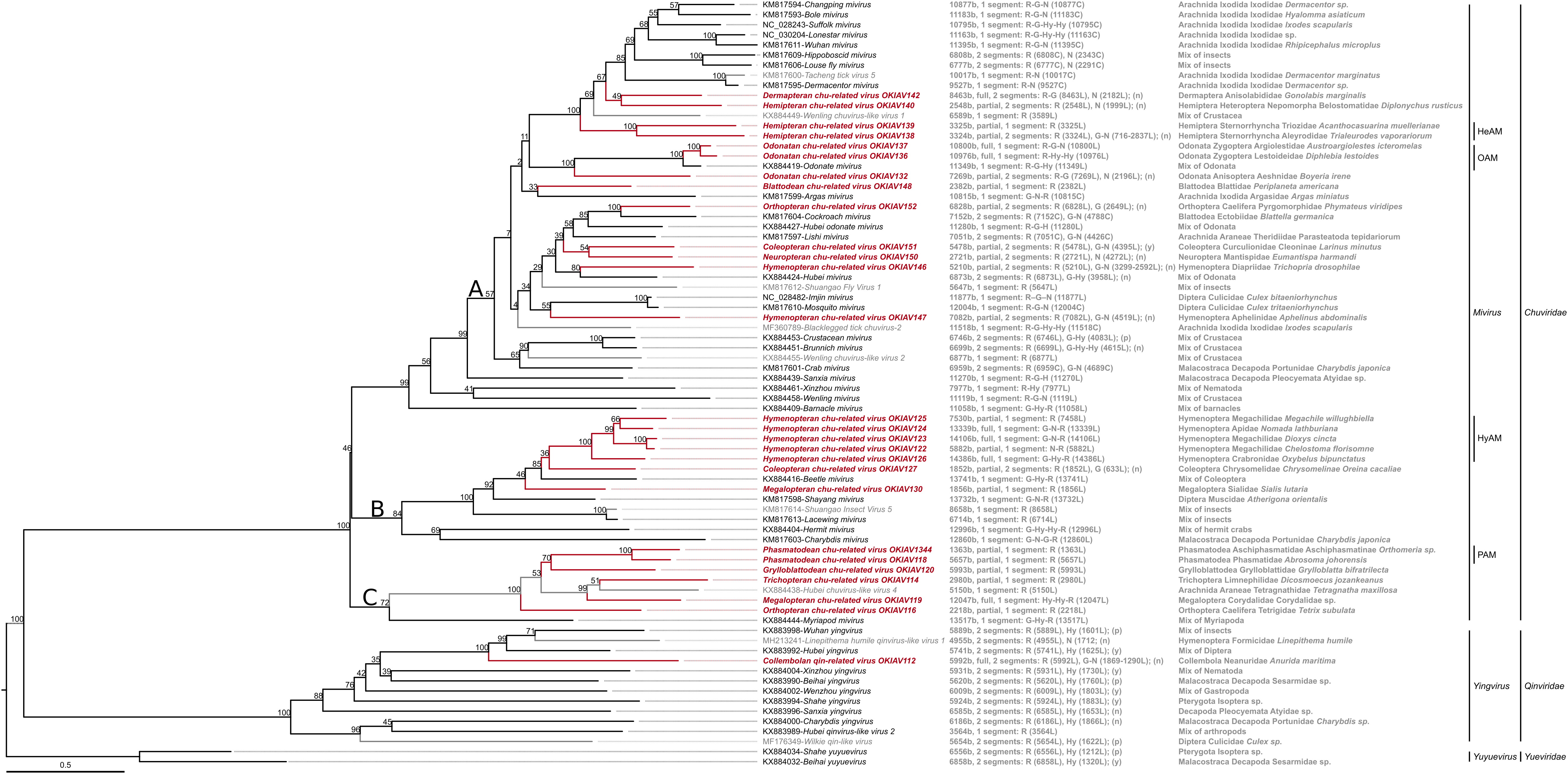

Supplement: S10 Fig — Black branches show ICTV-accepted taxa, grey branches show unclassified taxa, and red branches show OKIAVs. Columns on the right side summarize contig length, genome completeness, number of segments, taxonomic grouping of hosts, and viral genus and family. Genomic protein-coding regions are: R = RdRp, G = glycoprotein, N = nucleoprotein, Hy = hypothetical protein with unknown function. Segment length and organization are shown in parentheses: linear (L) or circular (C). For linear segments, information on the sequence similarity of segment ends among different genome segments is given as well: (n) = segment ends not matching the ends of the RdRp segment, (y) = segment ends matching the ends of the RdRp segment, (p) = segment ends partially matching the ends of the RdRp segment. The tree is rooted to Yuevirus (Yueviridae). (TIF) [file ppat.1008224.s015.tif]

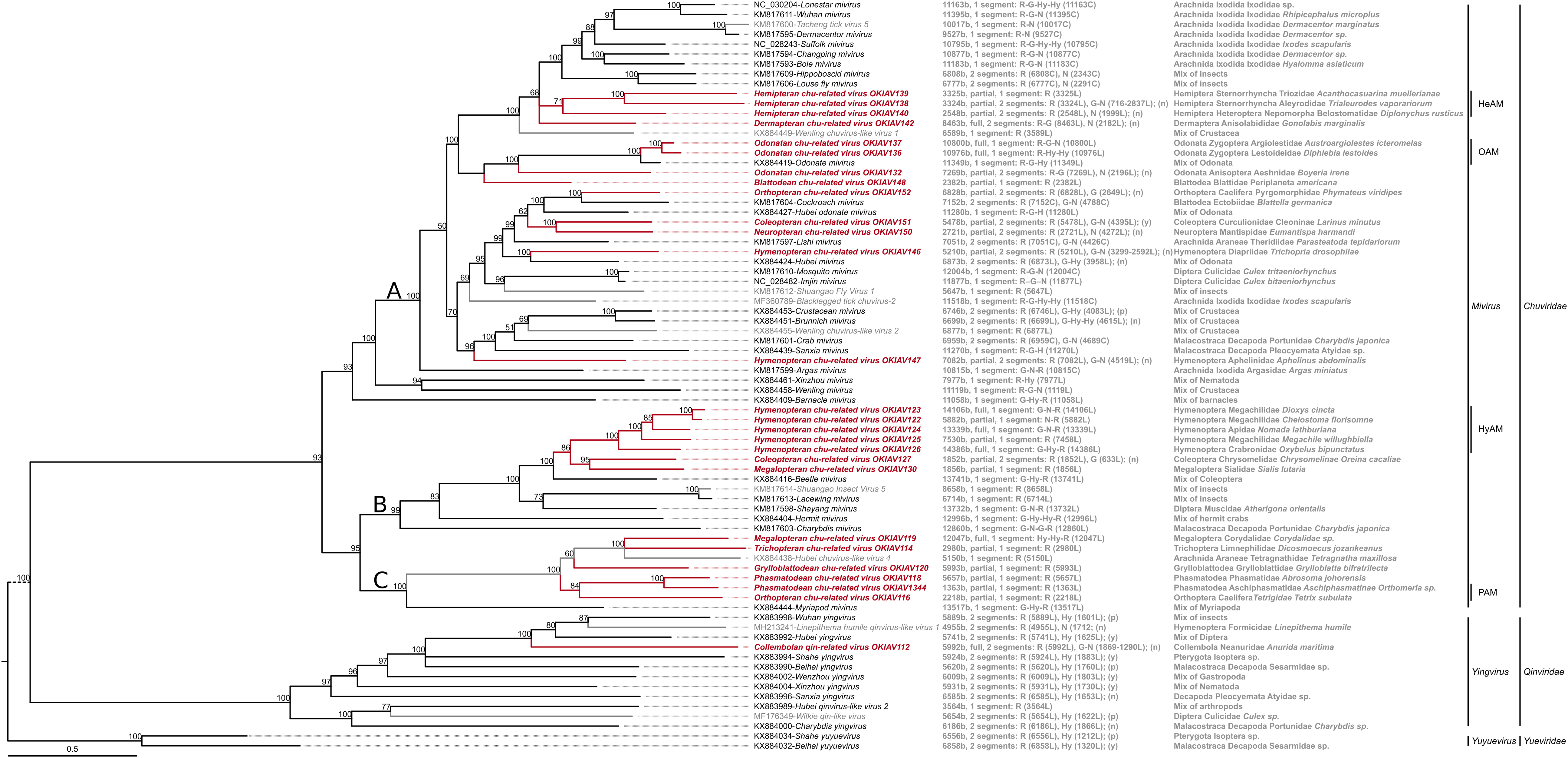

Supplement: S11 Fig — Black branches show ICTV-accepted taxa, grey branches show unclassified taxa, and red branches show OKIAVs. Columns on the right side summarize contig length, genome completeness, number of segments, taxonomic grouping of hosts, and viral genus and family. Genomic protein-coding regions are: R = RdRp, G = glycoprotein, N = nucleoprotein, Hy = hypothetical protein with unknown function. Segment length and organization are shown in parentheses: linear (L) or circular (C). For linear segments, information on the sequence similarity of segment ends among different genome segments is given as well: (n) = segment ends not matching the ends of the RdRp segment, (y) = segment ends matching the ends of the RdRp segment, (p) = segment ends partially matching the ends of the RdRp segment. The tree is rooted to Yuevirus (Yueviridae). (TIF) [file ppat.1008224.s016.tif]

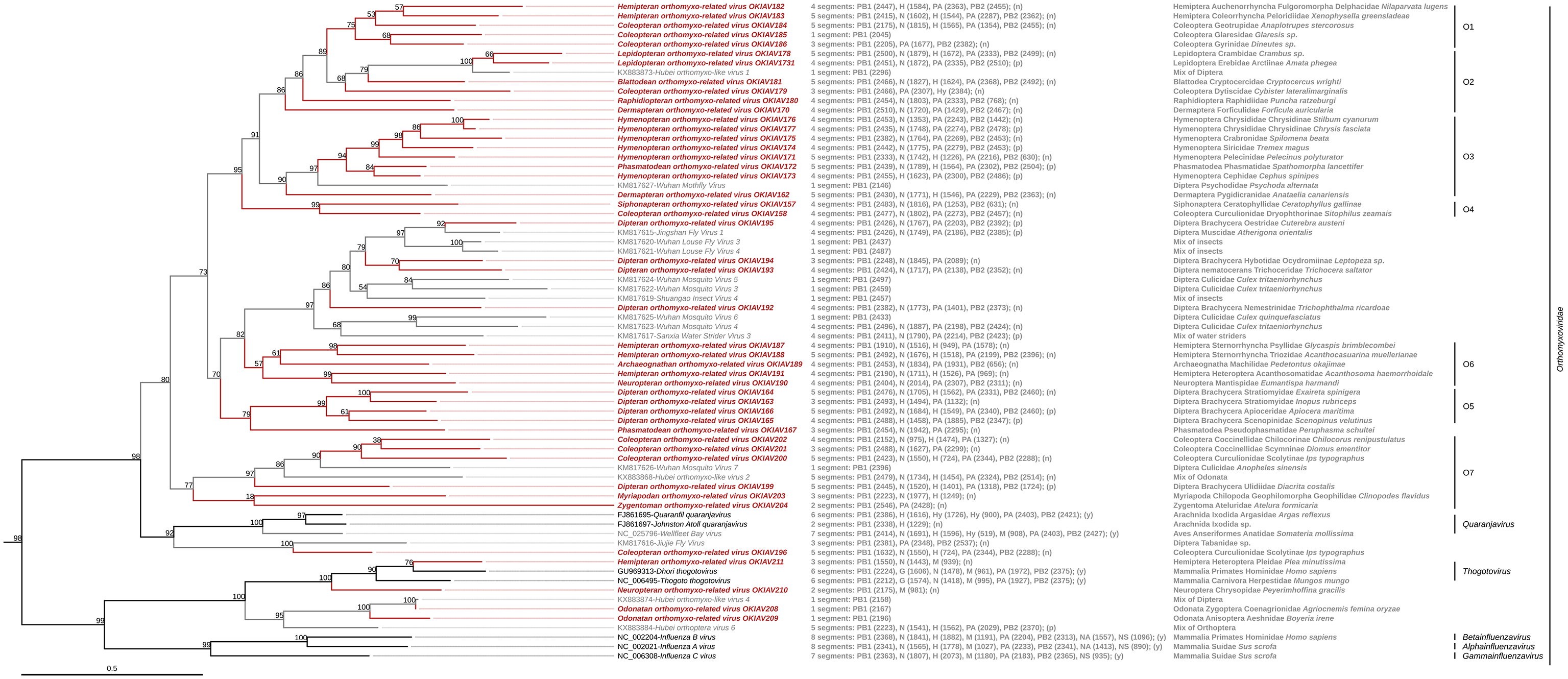

Supplement: S12 Fig — Maximum likelihood phylogenies based on RAxML. Black branches show ICTV-accepted taxa, grey branches show unclassified taxa, and red branches show OKIAVs. Columns on the right summarize contig length, genome completeness, number of segments, taxonomic grouping of hosts, and viral genus and family. Genomic protein-coding regions are: PB1 = polymerase subunit PB1, PB2 = polymerase subunit PB2, PA = polymerase subunit PA, G = glycoprotein, N = nucleoprotein, H = hemagglutinin, NA = neuraminidase, M = matrix protein, NS = non-structural protein, and Hy = hypothetical protein with unknown function. Segment lengths are shown in parentheses. Information on the segment ends is indicated by: (n) = segment ends not matching the ends of the PB1 segment, (y) = segment ends matching the ends of the PB1 segment, (p) = segment ends partially matching the ends of the PB1 segment. The outgroup taxon (not shown) is Salmon isavirus (Isavirus, Orthomyxoviridae). Analyses based on PhyML and MrBayes can be found in S13 and S14 Figs. (TIF) [file ppat.1008224.s017.tif]

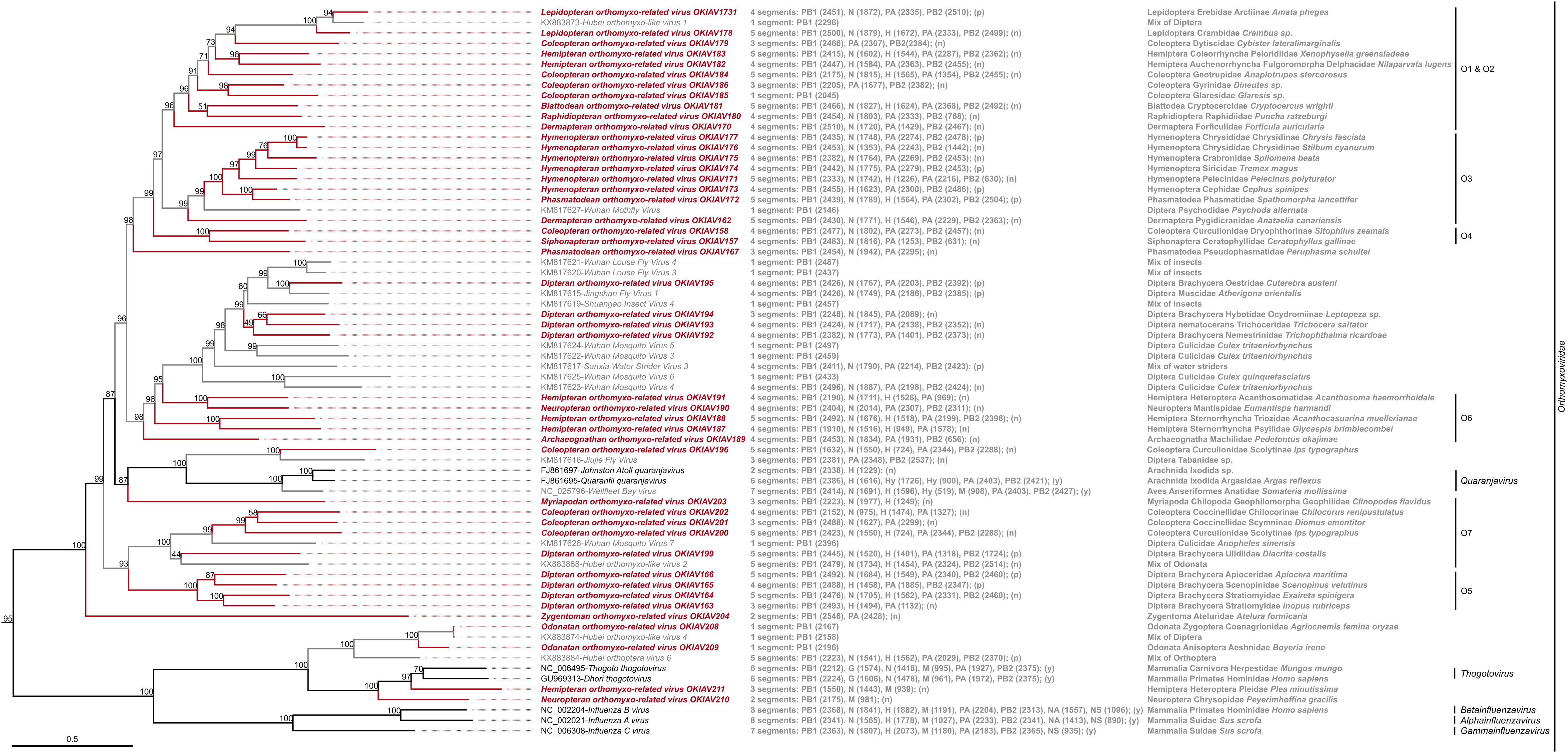

Supplement: S13 Fig — Black branches show ICTV-accepted taxa, grey branches show unclassified taxa, and red branches show OKIAVs. Columns on the right side summarize contig length, genome completeness, number of segments, taxonomic grouping of hosts, and viral genus and family. Genomic protein-coding regions are: PB1 = polymerase subunit PB1, PB2 = polymerase subunit PB2, PA = polymerase subunit PA, G = glycoprotein, N = nucleoprotein, H = hemagglutinin, NA = neuraminidase, M = matrix protein, NS = non-structural protein, and Hy = hypothetical protein with unknown function. Segment lengths are shown in parentheses. Information on the sequence similarity of segment ends among different genome segments is given as well: (n) = segment ends not matching the ends of the PB1 segment, (y) = segment ends matching the ends of the PB1 segment, (p) = segment ends partially matching the ends of the PB1 segment. The outgroup taxon (not shown) is Salmon isavirus (Isavirus, Orthomyxoviridae). (TIF) [file ppat.1008224.s018.tif]

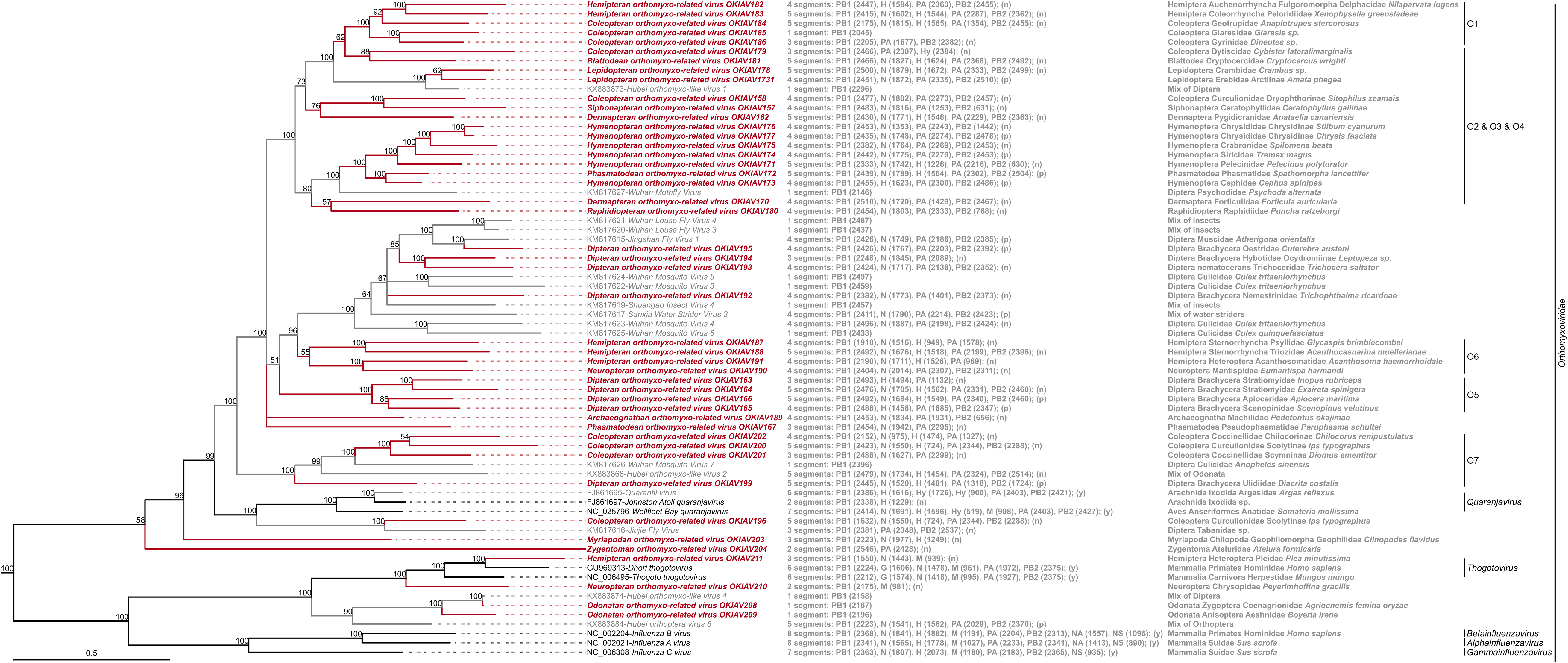

Supplement: S14 Fig — Black branches show ICTV-accepted taxa, grey branches show unclassified taxa, and red branches show OKIAVs. Columns on the right side summarize contig length, genome completeness, number of segments, taxonomic grouping of hosts, and viral genus and family. Genomic protein-coding regions are: PB1 = polymerase subunit PB1, PB2 = polymerase subunit PB2, PA = polymerase subunit PA, G = glycoprotein, N = nucleoprotein, H = hemagglutinin, NA = neuraminidase, M = matrix protein, NS = non-structural protein, and Hy = hypothetical protein with unknown function. Segment lengths are shown in parentheses. Information on the sequence similarity of segment ends among different genome segments is given as well: (n) = segment ends not matching the ends of the PB1 segment, (y) = segment ends matching the ends of the PB1 segment, (p) = segment ends partially matching the ends of the PB1 segment. The outgroup taxon (not shown) is Salmon isavirus (Isavirus, Orthomyxoviridae). (TIF) [file ppat.1008224.s019.tif]

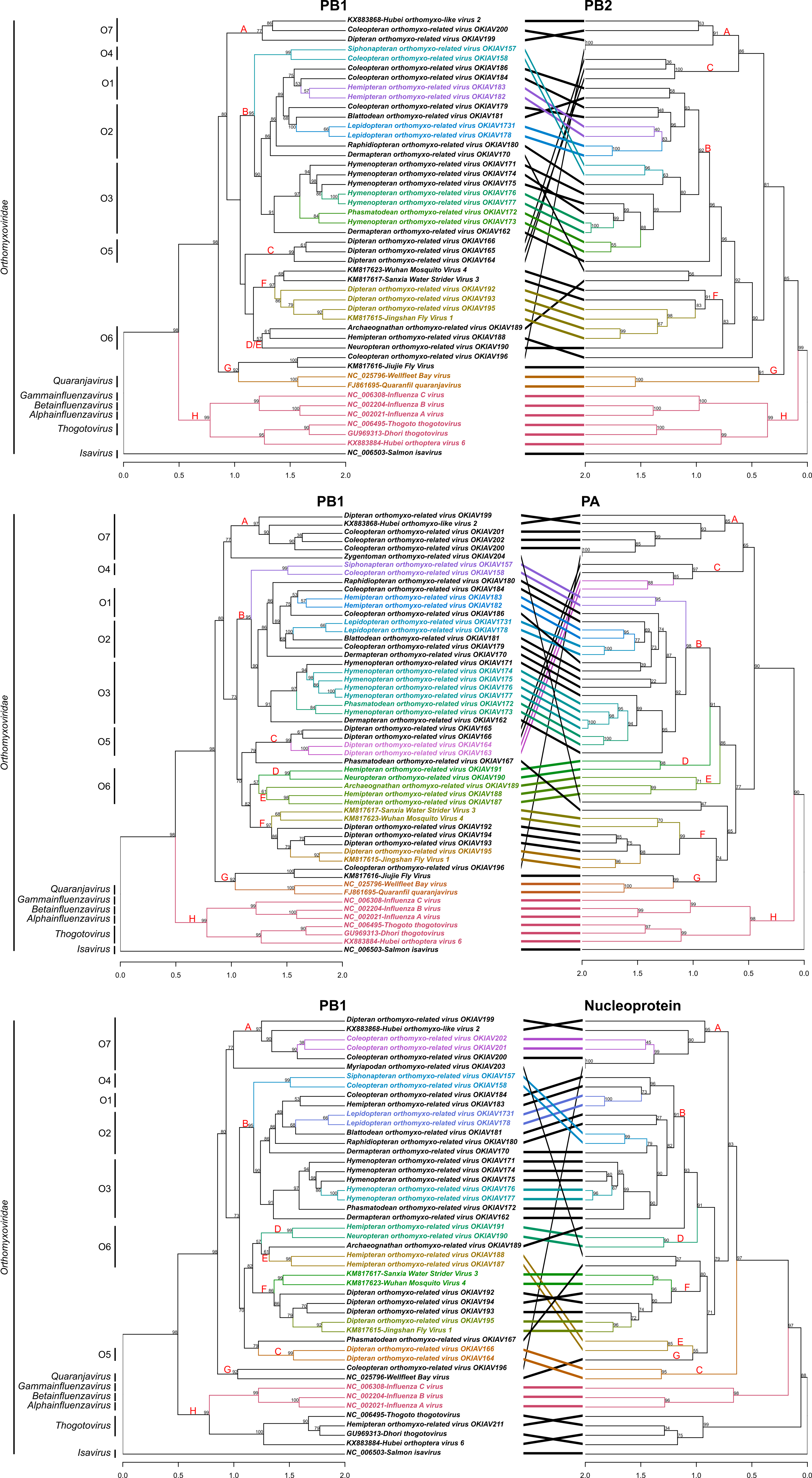

Supplement: S15 Fig — Topologically congruent clades are highlighted in color. Branches in black indicate taxa that do not share a common topological pattern in the respective tree pairs. Not all genomic segments have been identified for all viral taxa; therefore, the number of taxa varies among the tree pairs. Clade H, mainly composed of the Orthomyxoviridae genera, is not congruent between the PB1 and the nucleoprotein trees and shows two different topologies within Thogotovirus. The PB1 segments of Coleopteran orthomyxo-related virus OKIAV196 and -200 are both found within the same insect transcriptome (Ips typographus). Within this transcriptome we have also identified one PB2, one PA, and one nucleoprotein segments. The co-segregation analysis allowed us to assign all the latter segments to Coleopteran orthomyxo-related virus OKIAV200 rather than -196. In the phylogenies of PB1 and PA, 52 of 54 taxa are distributed in eight monophyletic clades. Clade B consists of three distinct inner clades that are topology-wise stable and share similarities within their inner parts. Whereas clades D and E are direct sisters in the PB1 tree, clade D is not directly linked to clade E in the PA tree. However, the PA tree has a higher support in this region of the tree. The position of clade G is maintained across all trees, except of Coleopteran orthomyxo-related virus OKIAV196 in the PA tree, which actually belongs next to Zygentoman orthomyxo-related virus OKIAV204 (clade A). Clade H consists exclusively of ICTV-accepted genera of Orthomyxoviridae, except Hubei orthoptera virus 6. These clades (G and H) show identical topology in both trees. In the phylogenies of PB1 and PB2, 43 of 46 taxa are distributed in six monophyletic clades. Clades D and E are merely represented by Archaeognathan orthomyxo-related virus OKIAV189, Hemipteran orthomyxo-related virus OKIAV188, and Neuropteran orthomyxo-related virus OKIAV190. Clade B shows a similar topology as in PA and PB1, and Clade G maintains its phyloge [file ppat.1008224.s020.tif]

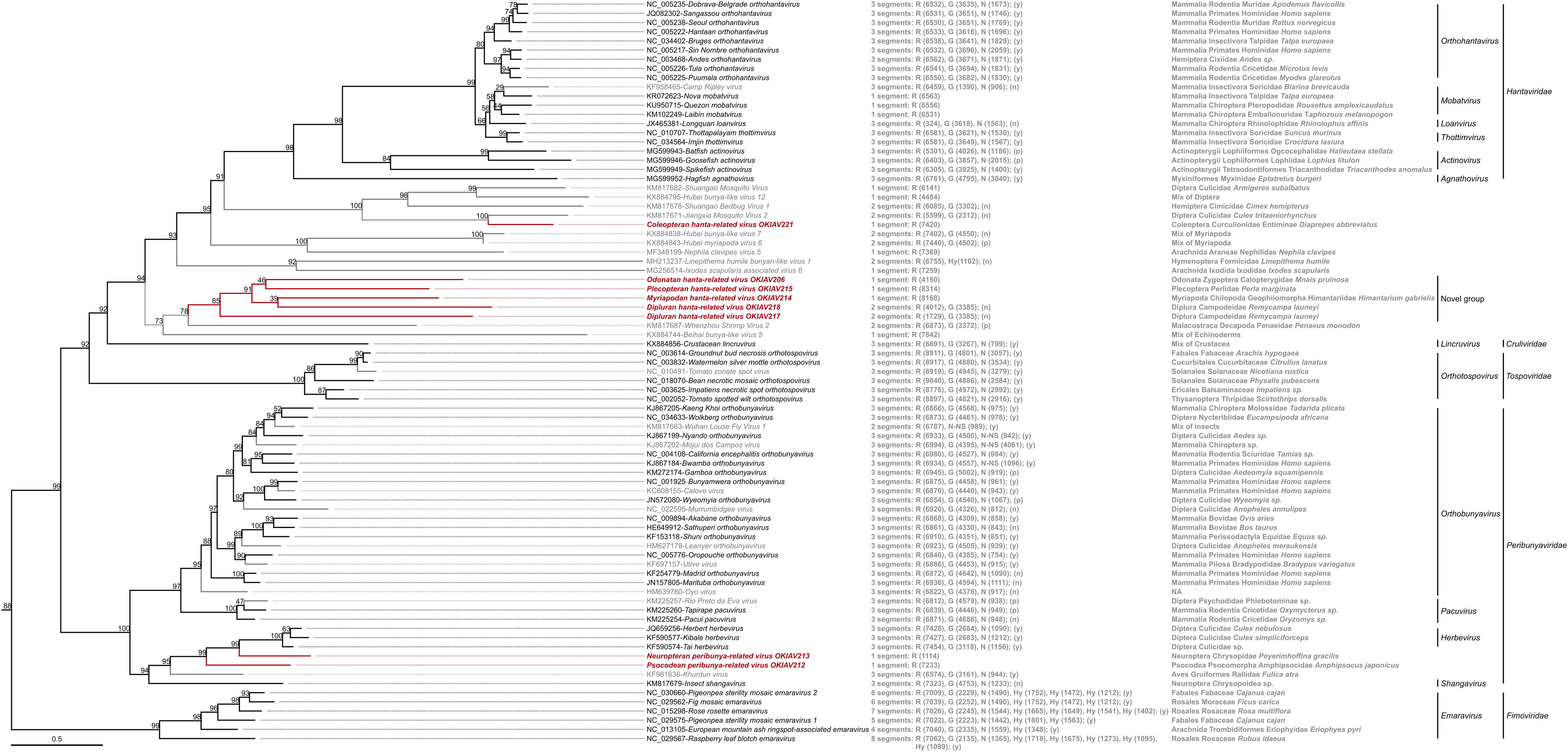

Supplement: S16 Fig — Maximum likelihood phylogenies based on RAxML. Black branches show ICTV-accepted taxa, grey branches show unclassified taxa, and red branches show OKIAVs. The outgroup taxon (not shown) is Collembolan hanta-related virus OKIAV223 (shown in S20 Fig). Analyses based on PhyML and MrBayes can be found in S17 and S18 Figs. (TIF) [file ppat.1008224.s021.tif]

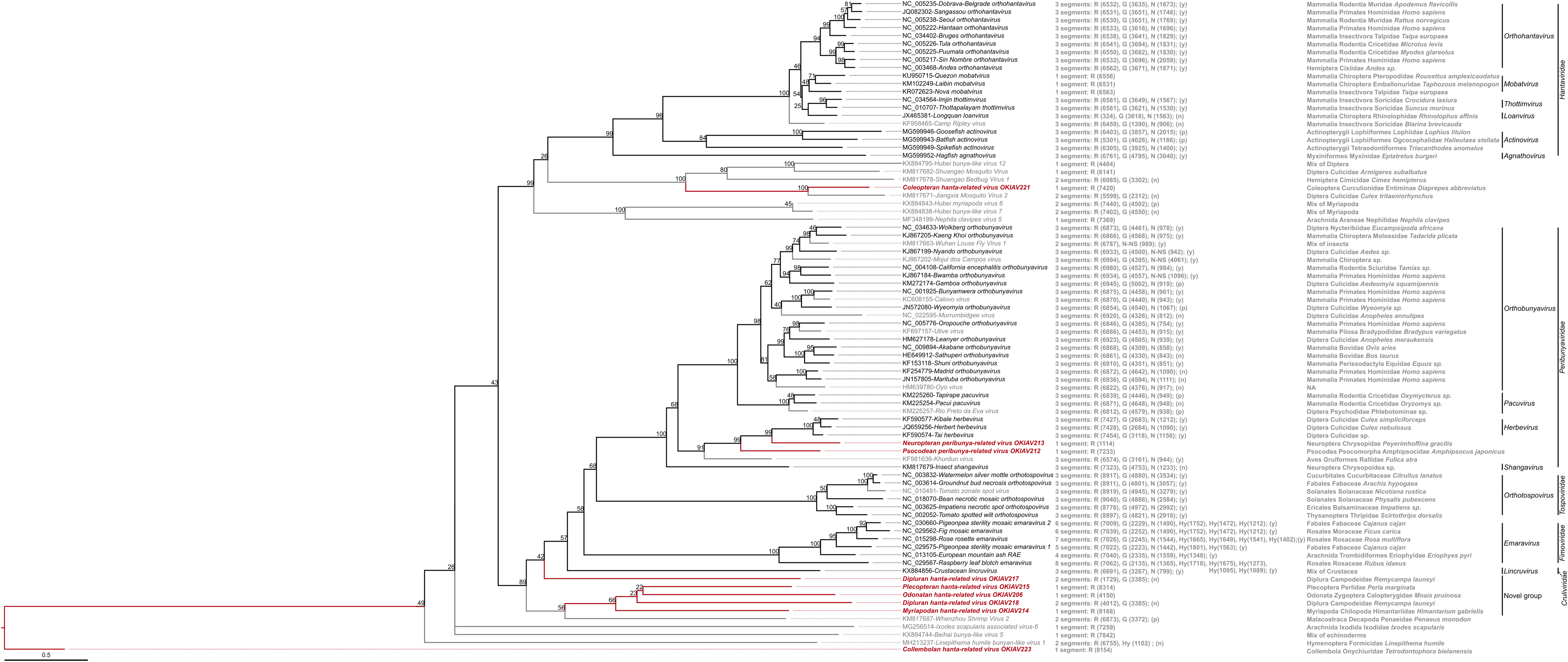

Supplement: S17 Fig — Black branches show ICTV-accepted taxa, grey branches show unclassified taxa, and red branches show OKIAVs. Columns on the right side summarize contig length, genome completeness, number of segments, taxonomic grouping of hosts, and viral genus and family. Genomic protein-coding regions are: R = RdRp, G = glycoprotein, N = nucleoprotein, Hy = hypothetical protein with unknown function. Segment lengths are shown in parentheses. Information on sequence similarity of the segment ends among different genome segments is given as well: (n) = segment ends not matching the ends of the RdRp segment, (y) = segment ends matching the ends of the RdRp segment, (p) = segment ends partially matching the ends of the RdRp segment. The outgroup taxon (not shown) is Collembolan hanta-related virus OKIAV223 (shown in S20 Fig). (TIF) [file ppat.1008224.s022.tif]

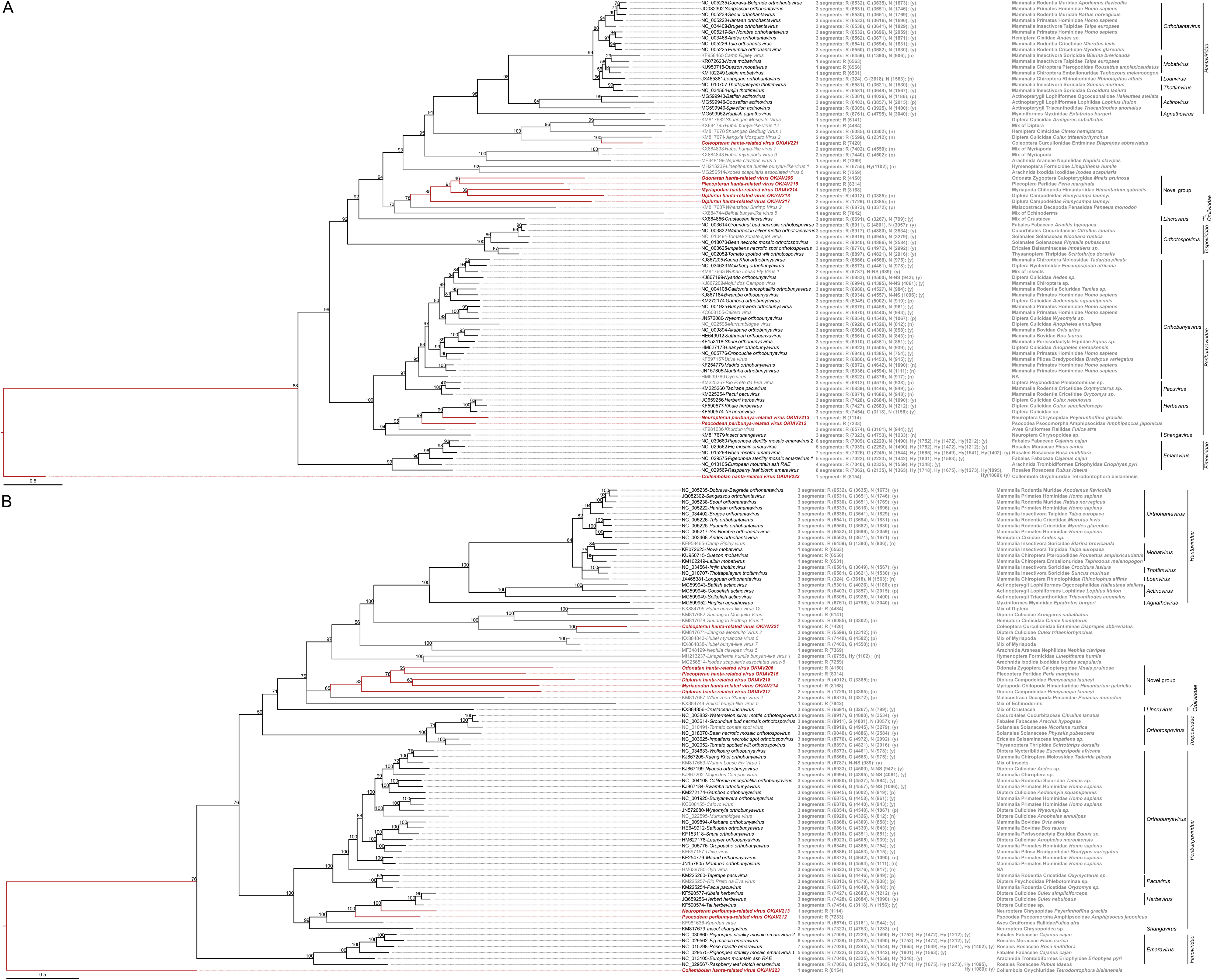

Supplement: S18 Fig — Maximum likelihood phylogeny based on RAxML (A) and Bayesian phylogeny inference with MrBayes (B) of viruses pertaining to Hantaviridae, Cruliviridae, Peribunyaviridae, and Fimoviridae. Black branches show ICTV-accepted taxa, grey branches show unclassified taxa, and red branches show OKIAVs. Columns on the right side summarize contig length, genome completeness, number of segments, taxonomic grouping of hosts, and viral genus and family. Genomic protein-coding regions are: R = RdRp, G = glycoprotein, N = nucleoprotein, Hy = hypothetical protein with unknown function. Segment lengths are shown in parentheses. Information on sequence similarity of the segment ends among different genome segments is given as well: (n) = segment ends not matching the ends of the RdRp segment, (y) = segment ends matching the ends of the RdRp segment, (p) = segment ends partially matching the ends of the RdRp segment. The outgroup taxon (not shown) is Collembolan hanta-related virus OKIAV223 (shown in S20 Fig). (TIF) [file ppat.1008224.s023.tif]

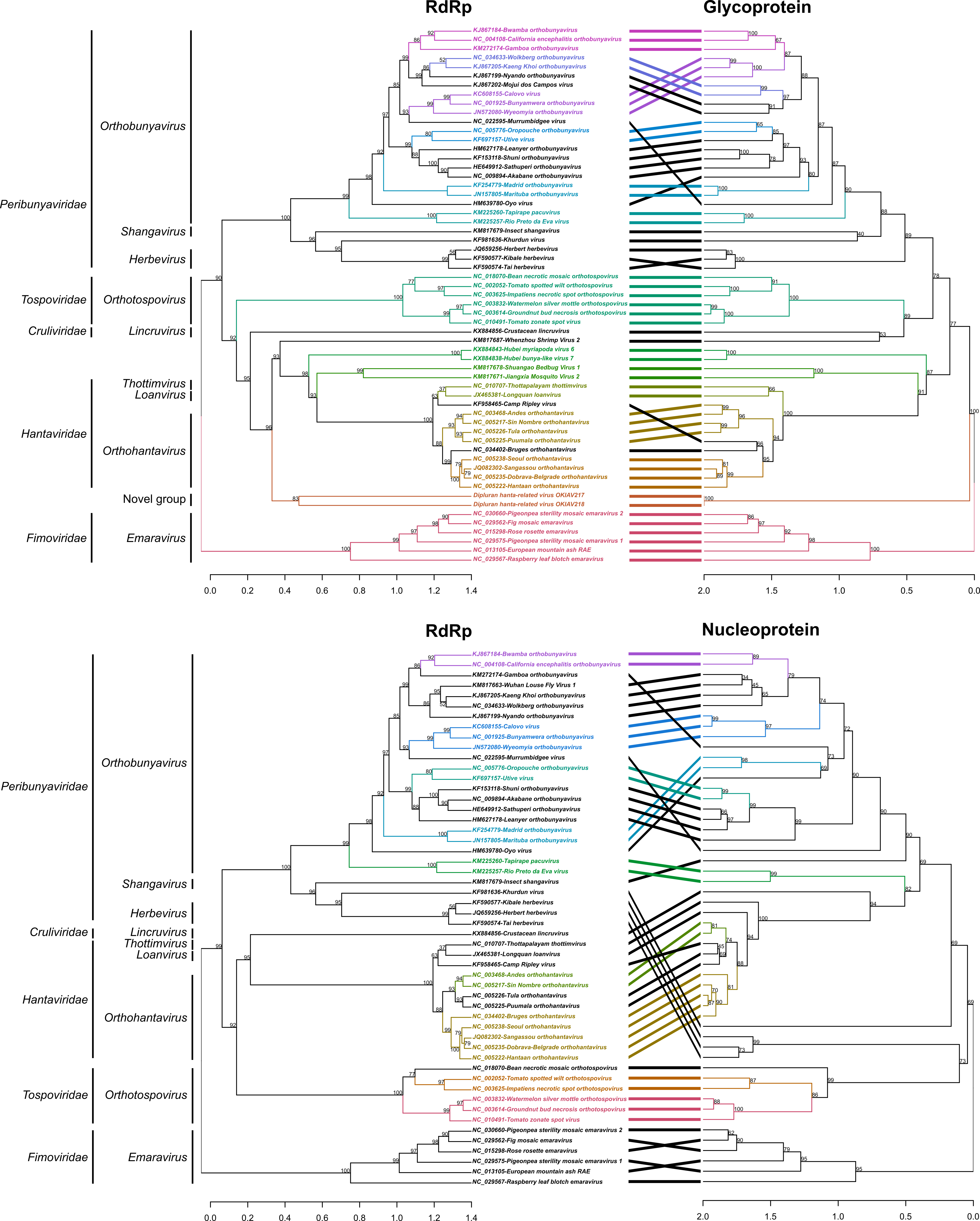

Supplement: S19 Fig — Topologically congruent clades are highlighted in color. Branches in black indicate taxa that do not share a common topological pattern in the respective tree pairs. In the phylogenies of RdRp and glycoprotein, 55 of 59 taxa were distributed in five monophyletic clades represented by the genera Orthobunyavirus, Herbevirus, Tospovirus, Orthohantavirus, and Emaravirus. Despite spanning four different families, the phylogenies are well-supported and some of the (sub)topologies can be confirmed. Tospovirus and Emaravirus have a completely congruent topology, while Orthobunyavirus and Orthohantavirus have some topologically stable subclades. Noteworthy are not only the congruent topologies, but also the very similar branch lengths of Tospovirus, Orthohantavirus, and Emaravirus. Based only on the tree topology, an assignment of the M-segment to either Dipluran hanta-related virus OKIAV217 or -218 cannot be assessed. In the phylogenies of RdRp and nucleoprotein, 44 of 49 taxa distributed in the genera Orthobunyavirus, Herbevirus, Tospovirus, Orthohantavirus, and Emaravirus. Tospovirus is the only genus that retains its inner topological structure among all trees, except of the position of Bean necrotic mosaic virus, that in the nucleoprotein tree is sister to all other tospoviruses. However, the position of Tospovirus within Peribunyaviridae is not supported by the nucleoprotein phylogeny. Apart from that, all genera are still monophyletic. The position of Khurdun virus as the first split from Herbevirus can be confirmed by both nucleoprotein and glycoprotein phylogenies. (TIF) [file ppat.1008224.s024.tif]

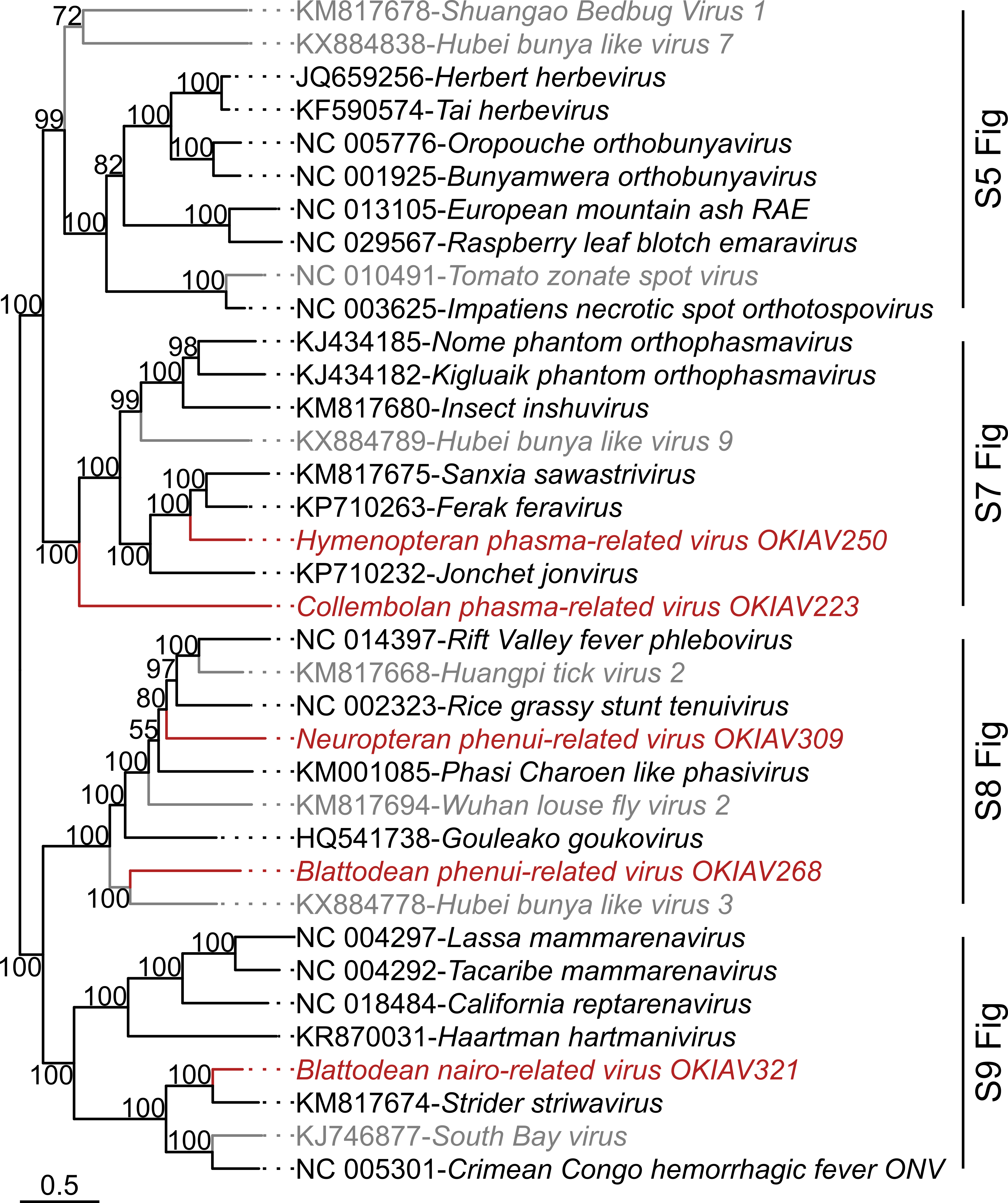

Supplement: S20 Fig — Bayesian inference of phylogeny based on MrBayes. Black branches show selected reference taxa, and red branches show some OKIAVs. (TIF) [file ppat.1008224.s025.tif]

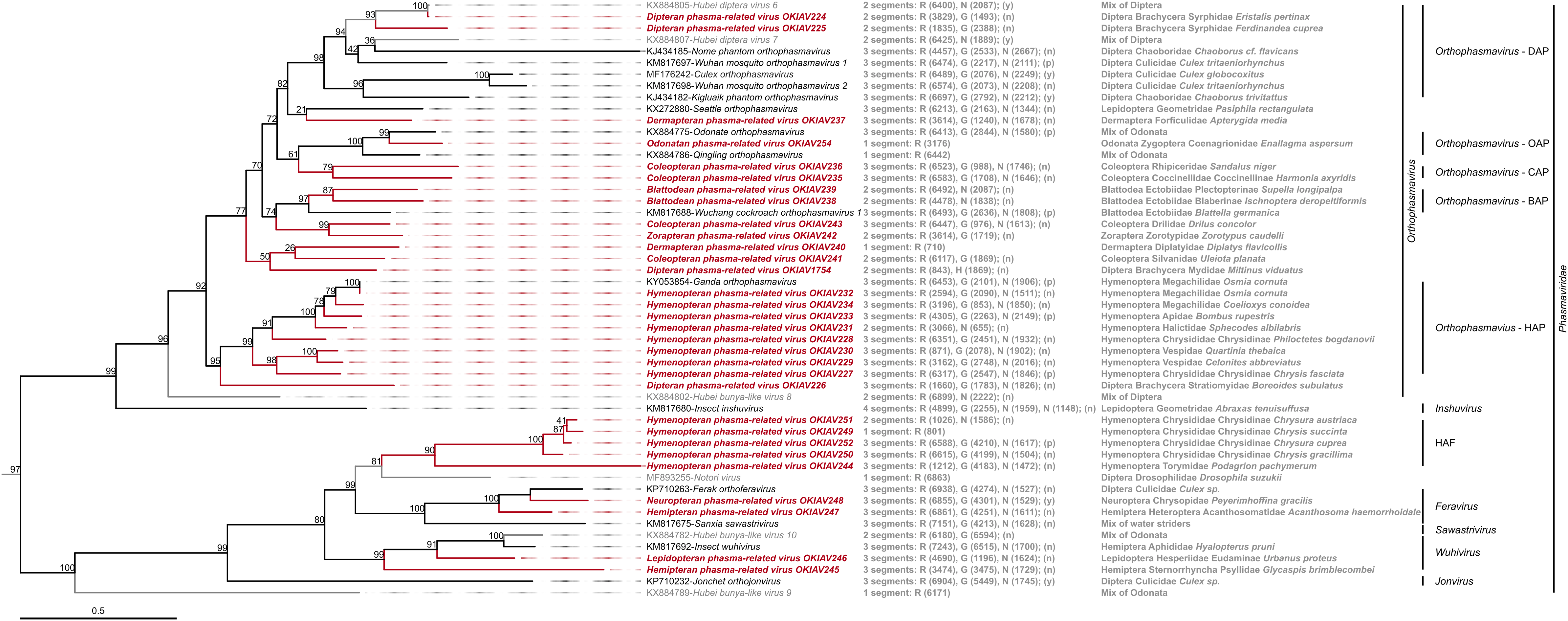

Supplement: S21 Fig — Maximum likelihood phylogenies based on RAxML. Black branches show ICTV-accepted taxa, grey branches show unclassified taxa, and red branches show OKIAVs. Columns on the right summarize contig length, genome completeness, number of segments, taxonomic grouping of hosts, and viral genus and family. Genomic protein-coding regions are: R = RdRp, G = glycoprotein, N = nucleoprotein, Hy = hypothetical protein with unknown function. Segment lengths are shown in parentheses. Information on the segment ends is indicated by: (n) = segment ends not matching the ends of the RdRp segment, (y) = segment ends matching the ends of the RdRp segment, (p) = segment ends partially matching the ends of the RdRp segment. The tree was rooted using a hantavirus outgroup. The outgroup was then removed, the tree recalculated, and the rooting between Orthophasmavirus vs. (HAF, Feravirus, Wuhivirus, and Jonvirus) was maintained. Analyses based on PhyML and MrBayes can be found in S22 and S23 Figs. (TIF) [file ppat.1008224.s026.tif]

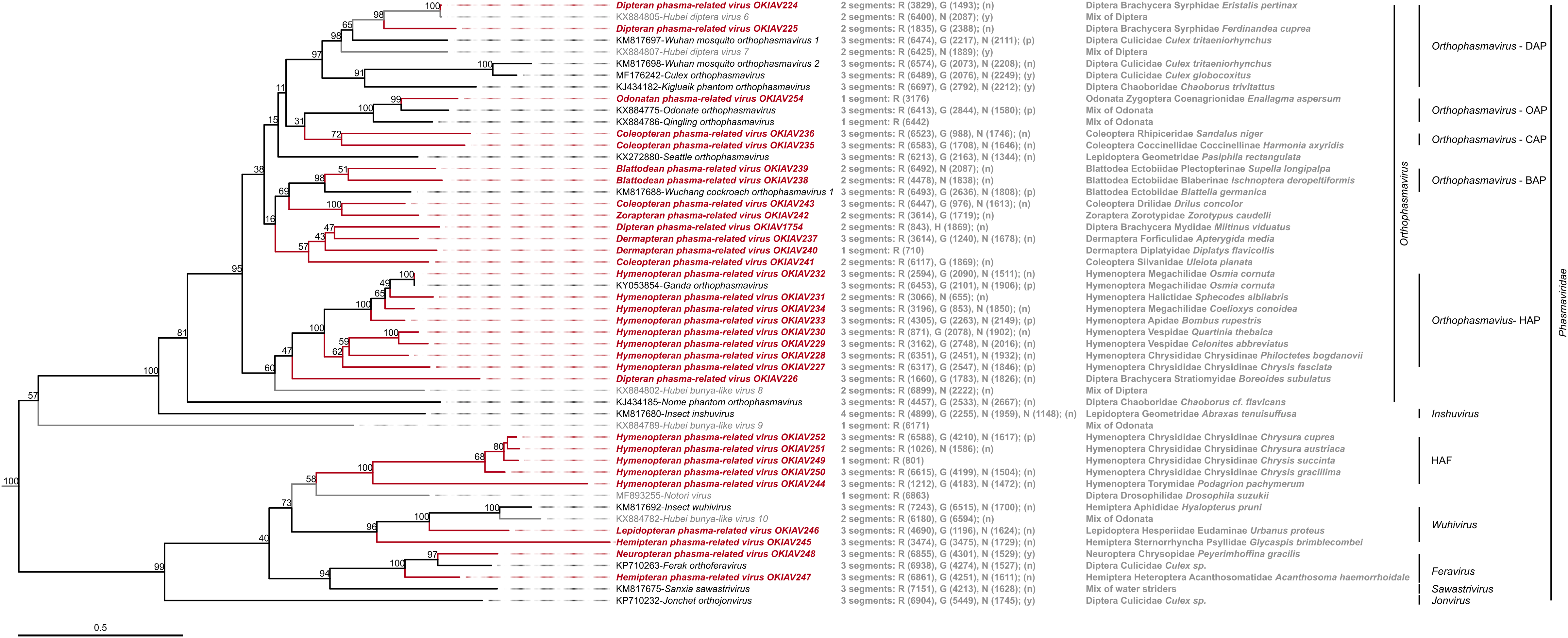

Supplement: S22 Fig — Black branches show ICTV-accepted taxa, grey branches show unclassified taxa, and red branches show OKIAVs. Columns on the right side summarize contig length, genome completeness, number of segments, taxonomic grouping of hosts, and viral genus and family. Genomic protein-coding regions are: R = RdRp, G = glycoprotein, N = nucleoprotein, Hy = hypothetical protein with unknown function. Segment lengths are shown in parentheses. Information on sequence similarity of the segment ends among different genome segments is given as well: (n) = segment ends not matching the ends of the RdRp segment, (y) = segment ends matching the ends of the RdRp segment, (p) = segment ends partially matching the ends of the RdRp segment. The tree is rooted to the exclusion of the lower clade of Phasmaviridae (HAF, Feravirus, Wuhivirus, Jonvirus) following preliminary analyses using a hantavirus outgroup. (TIF) [file ppat.1008224.s027.tif]

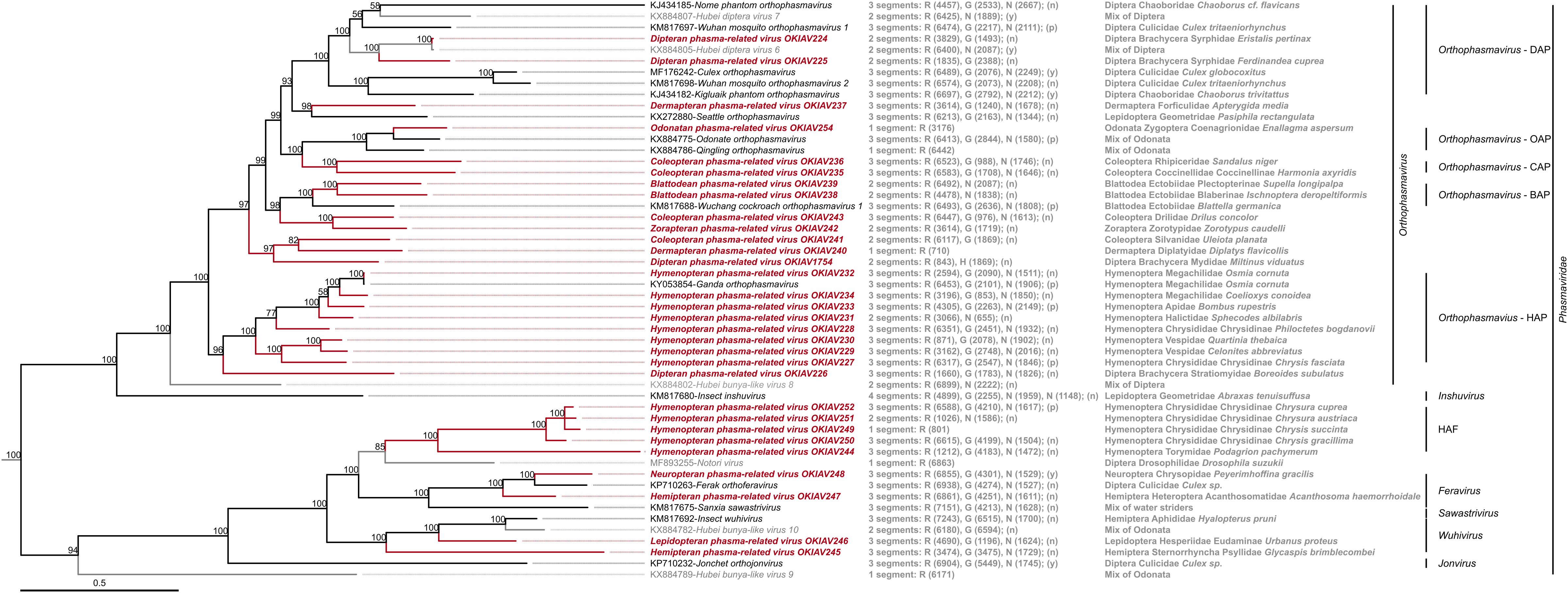

Supplement: S23 Fig — Black branches show ICTV-accepted taxa, grey branches show unclassified taxa, and red branches show OKIAVs. Columns on the right side summarize contig length, genome completeness, number of segments, taxonomic grouping of hosts, and viral genus and family. Genomic protein-coding regions are: R = RdRp, G = glycoprotein, N = nucleoprotein, Hy = hypothetical protein with unknown function. Segment lengths are shown in parentheses. Information on sequence similarity of the segment ends among different genome segments is given as well: (n) = segment ends not matching the ends of the RdRp segment, (y) = segment ends matching the ends of the RdRp segment, (p) = segment ends partially matching the ends of the RdRp segment. The tree is rooted to the lower clade of Phasmaviridae (HAF, Feravirus, Wuhivirus, Jonvirus) following preliminary analyses using a hantavirus outgroup. (TIF) [file ppat.1008224.s028.tif]

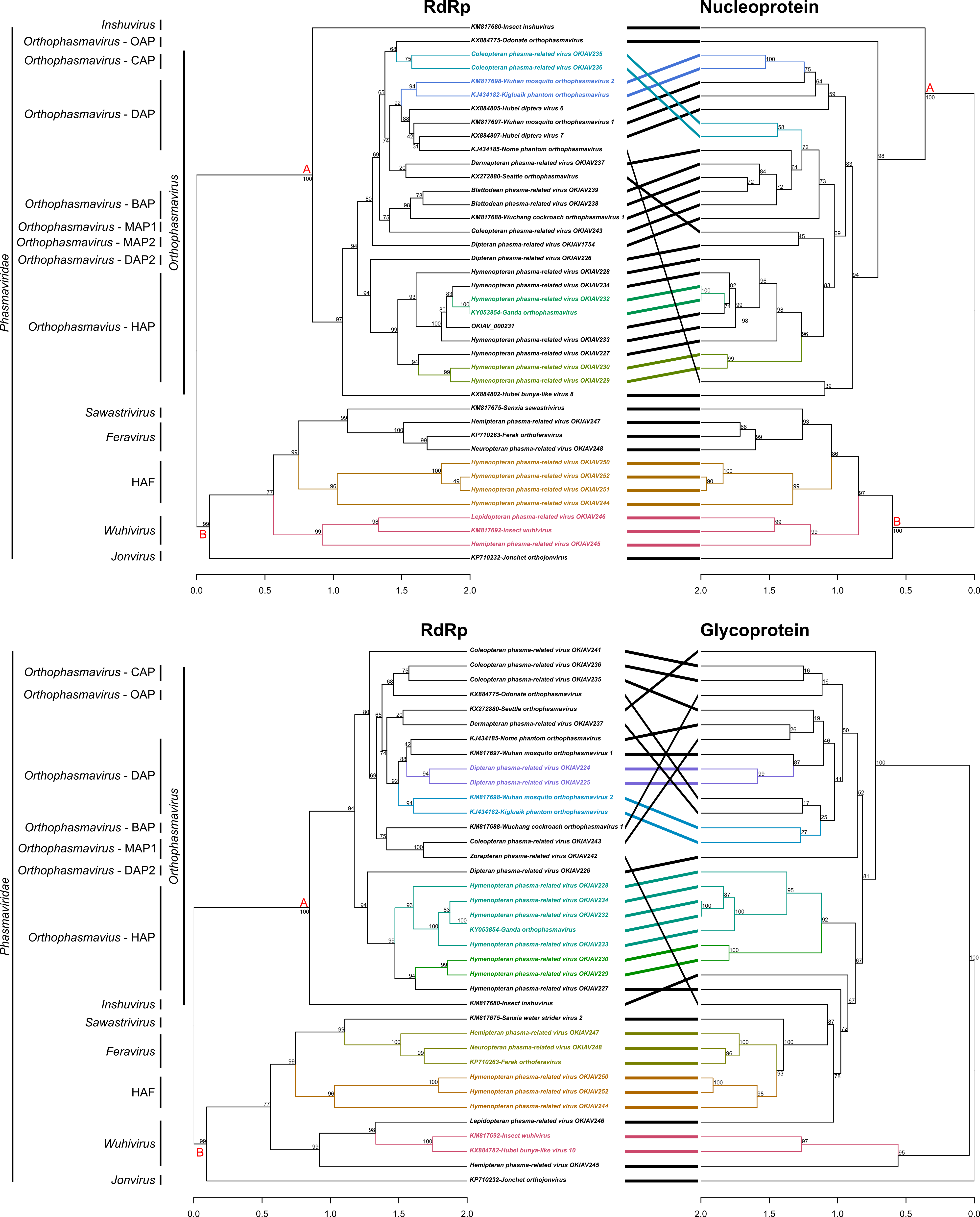

Supplement: S24 Fig — Topologically congruent clades are highlighted in color. Branches in black indicate taxa that do not share a common topological pattern in the respective tree pairs. In the phylogenies of RdRp and nucleoprotein, 34 of 40 taxa were distributed in three monophyletic clades that consist of the ICTV-accepted genera Feravirus, Wuhivirus, and Orthophasmavirus. In the nucleoprotein phylogeny, clades DAP and HAP form inner congruent monophyletic groups within Orthophasmavirus. The same pattern applies to 30 of 36 taxa in the glycoprotein phylogeny. Within Orthophasmavirus, HAP is the most stable clade, with a subclade of five taxa that have identical topology among the phylogenies. However, the bootstrap support of the Orthophasmavirus subclades is below 80%. The clear subdivision into clades A and B on the RdRp and nucleoprotein trees is not verified in the glycoprotein phylogeny, yet Feravirus and HAF group together with a high support (99%), and maintain their inner topological structures. Most of the Orthophasmavirus taxa have not been subjected to laboratory studies. Additionally, the OKIAV sequences are only fragmentarily assembled. Obtaining stable and congruent phylogenies among genomic segments can thus not be expected. (TIF) [file ppat.1008224.s029.tif]

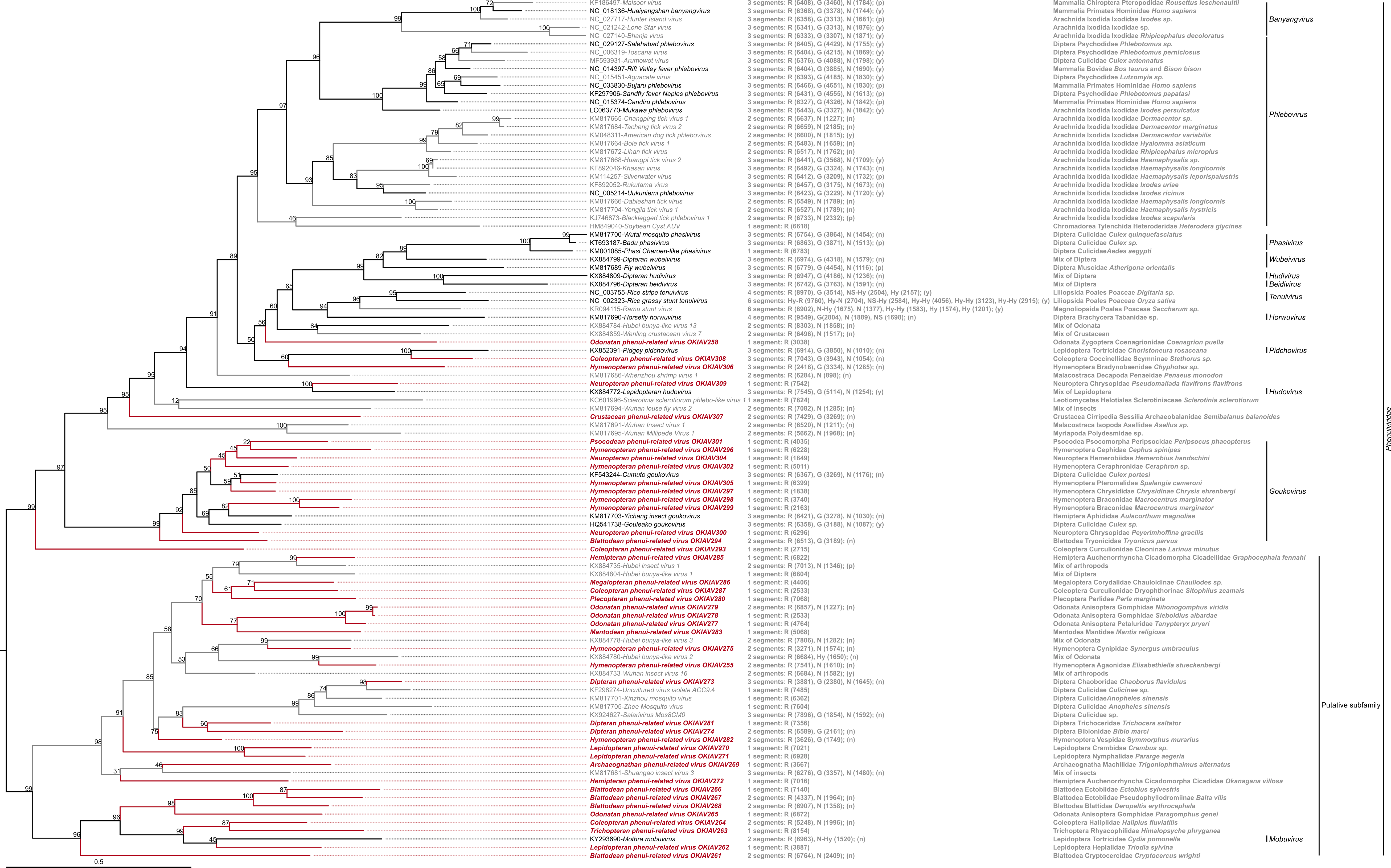

Supplement: S25 Fig — Maximum likelihood phylogenies based on RAxML. Black branches show ICTV-accepted taxa, grey branches show unclassified taxa, and red branches show OKIAVs. Columns on the right summarize contig length, genome completeness, number of segments, taxonomic grouping of hosts, and viral genus and family. Genomic protein-coding regions are: R = RdRp, G = glycoprotein, N = nucleoprotein, Hy = hypothetical protein with unknown function. Segment lengths are shown in parentheses. Information on the segment ends is indicated by: (n) = segment ends not matching the ends of the RdRp segment, (y) = segment ends matching the ends of the RdRp segment, (p) = segment ends partially matching the ends of the RdRp segment. The tree is rooted to the putative subfamily. Analyses based on PhyML and MrBayes can be found in S26 and S27 Figs. (TIF) [file ppat.1008224.s030.tif]

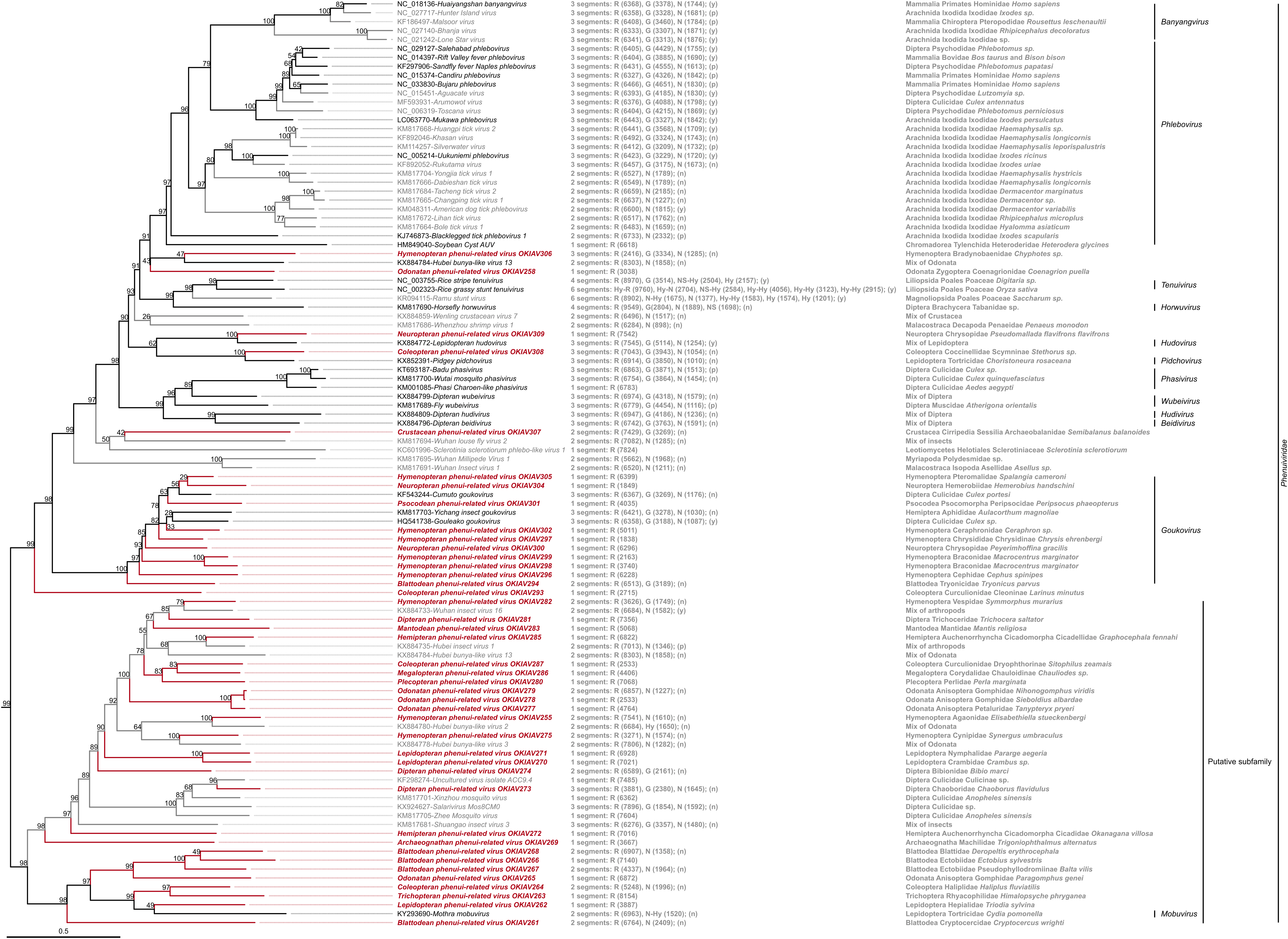

Supplement: S26 Fig — Black branches show ICTV-accepted taxa, grey branches show unclassified taxa, and red branches show OKIAVs. Columns on the right side summarize contig length, genome completeness, number of segments, taxonomic grouping of hosts, and viral genus and family. Genomic protein-coding regions are: R = RdRp, G = glycoprotein, N = nucleoprotein, Hy = hypothetical protein with unknown function. Segment lengths are shown in parentheses. Information on sequence similarity of the segment ends among different genome segments is given as well: (n) = segment ends not matching the ends of the RdRp segment, (y) = segment ends matching the ends of the RdRp segment, (p) = segment ends partially matching the ends of the RdRp segment. The tree is rooted to the putative subfamily. (TIF) [file ppat.1008224.s031.tif]

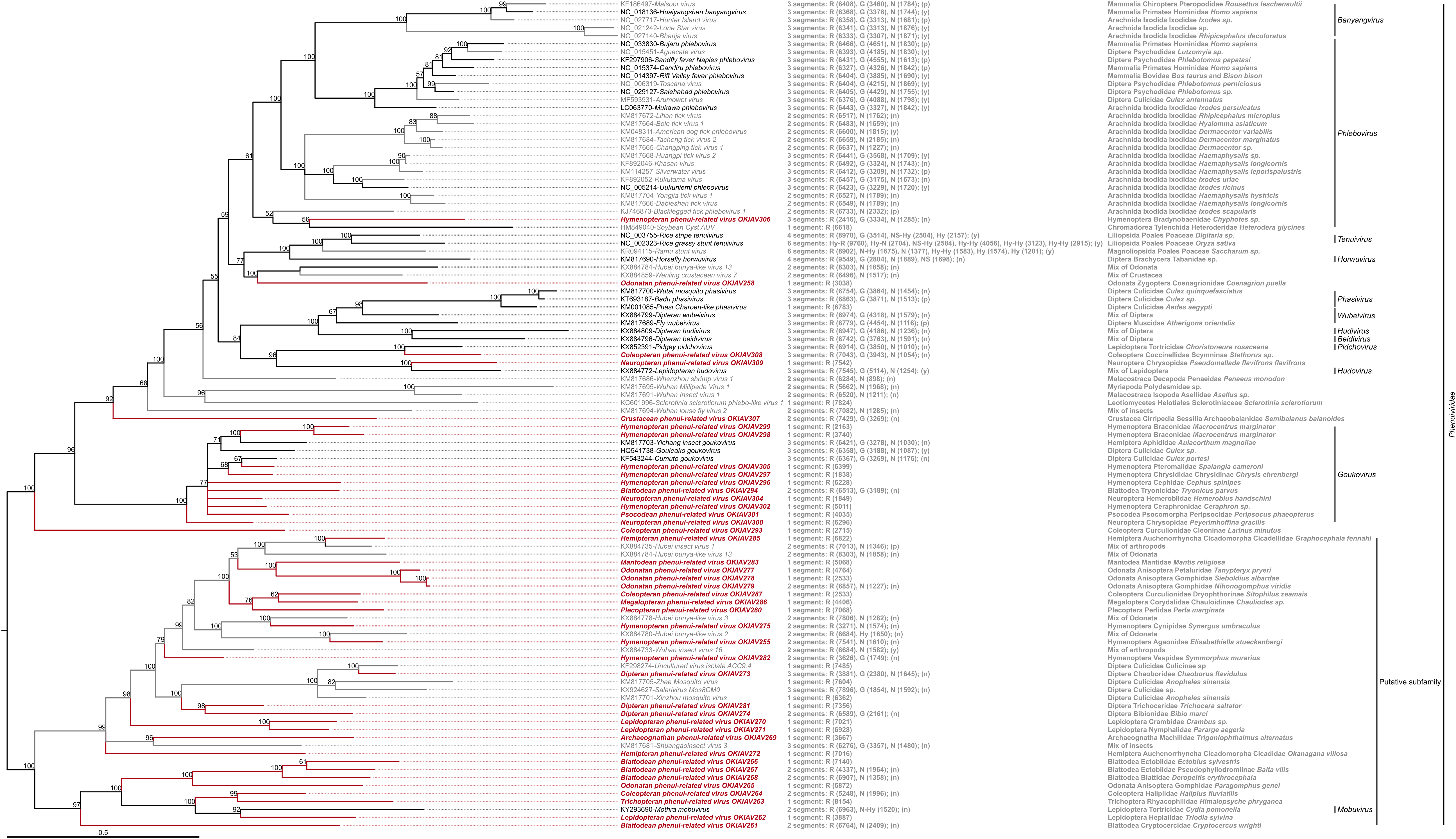

Supplement: S27 Fig — Black branches show ICTV-accepted taxa, grey branches show unclassified taxa, and red branches show OKIAVs. Columns on the right side summarize contig length, genome completeness, number of segments, taxonomic grouping of hosts, and viral genus and family. Genomic protein-coding regions are: R = RdRp, G = glycoprotein, N = nucleoprotein, Hy = hypothetical protein with unknown function. Segment lengths are shown in parentheses. Information on sequence similarity of the segment ends among different genome segments is given as well: (n) = segment ends not matching the ends of the RdRp segment, (y) = segment ends matching the ends of the RdRp segment, (p) = segment ends partially matching the ends of the RdRp segment. The tree is rooted to the putative subfamily. (TIF) [file ppat.1008224.s032.tif]

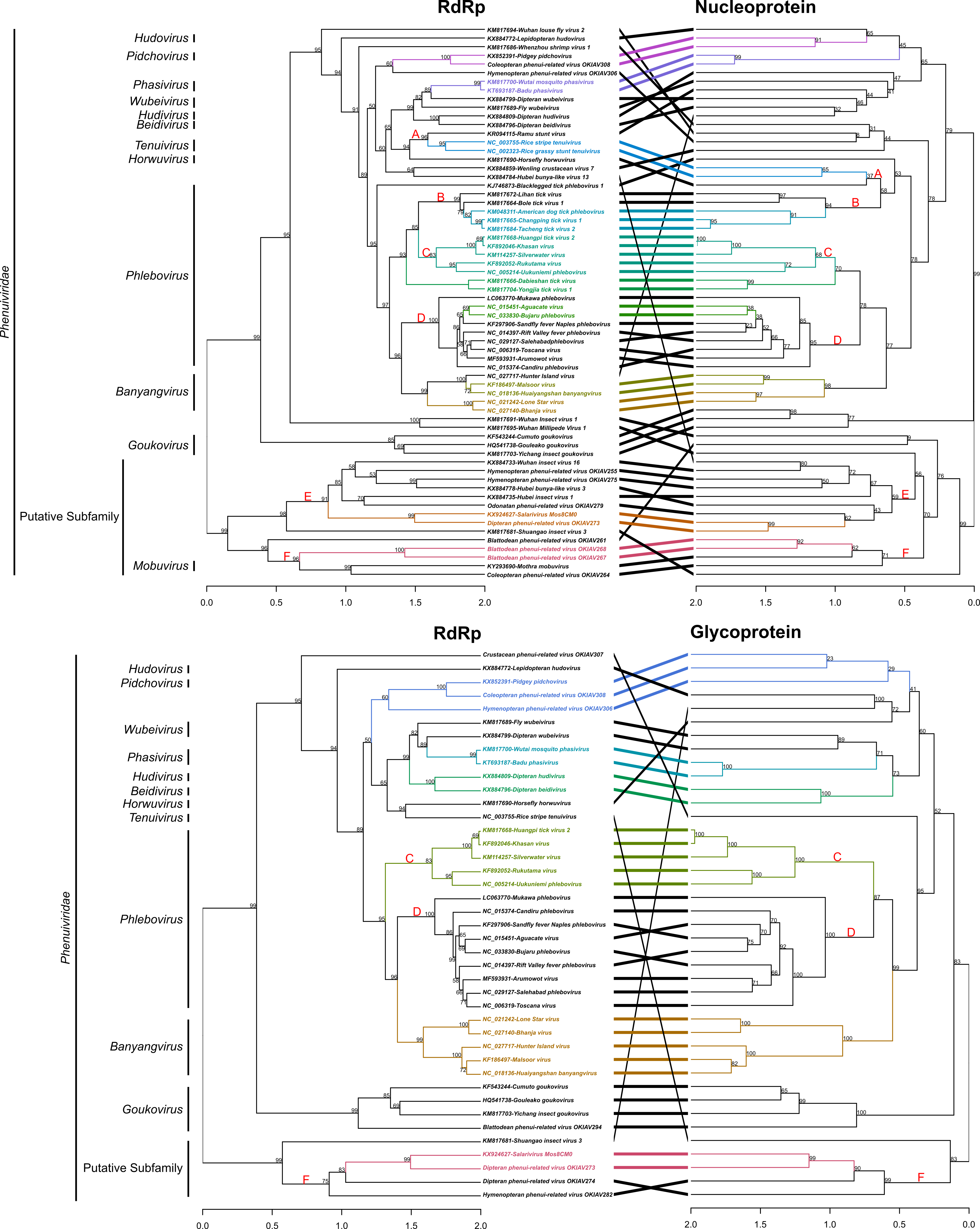

Supplement: S28 Fig — Topologically congruent clades are highlighted in color. Branches in black indicate taxa that do not share a common topological pattern in the respective tree pairs. The lack of complete genomes for most of the taxa that appear on the trees causes high topological conflict between the phylogenies of the different segments. The glycoprotein- and nucleoprotein-segments have not been identified for most of the OKIAV. The bootstrap support on the clades of the single-species genera Hudovirus, Pidchovirus, Hudivirus, Beidivirus, and Horwuvirus is low in comparison to the rest of the tree. Thus, the phylogenetic signal is probably not sufficient to draw meaningful conclusions on co-segregations for these genera. Additionally, within the putative subfamily, the lack of genomic segments for co-segregation analysis does not allow us drawing conclusions either. In the phylogenies of RdRp and nucleoprotein, 45 of 64 taxa are distributed in topologically stable monophyletic clades within the genera Phasivirus, Wubeivirus, Tenuivirus, Phlebovirus (with the exception of clade B), Banyangvirus, Goukovirus, and additionally the unclassified clades A, E, and F. Within Phlebovirus, clade B is sister to clade A and includes the genus Tenuivirus. Clade C is topologically congruent among all three phylogenies. Both clades C and D maintain their taxa composition across all three trees as well as their relation to Banyangvirus. The topological stability of the Banyangvirus clade within the Phlebovirus clade, suggests that Banyangvirus should rather be classified as a sub-genus of Phlebovirus. In the phylogenies of RdRp and glycoprotein, 35 of 41 taxa are distributed in topologically stable monophyletic clades that are accepted genera and the unclassified clades D, E, and F. Hudivirus and Beidivirus group together in this case, indicating a close relationship. A doubtful classification is the one of Wubeivirus: it is monophyletic only in the glycoprotein phylogeny, but groups consistently [file ppat.1008224.s033.tif]

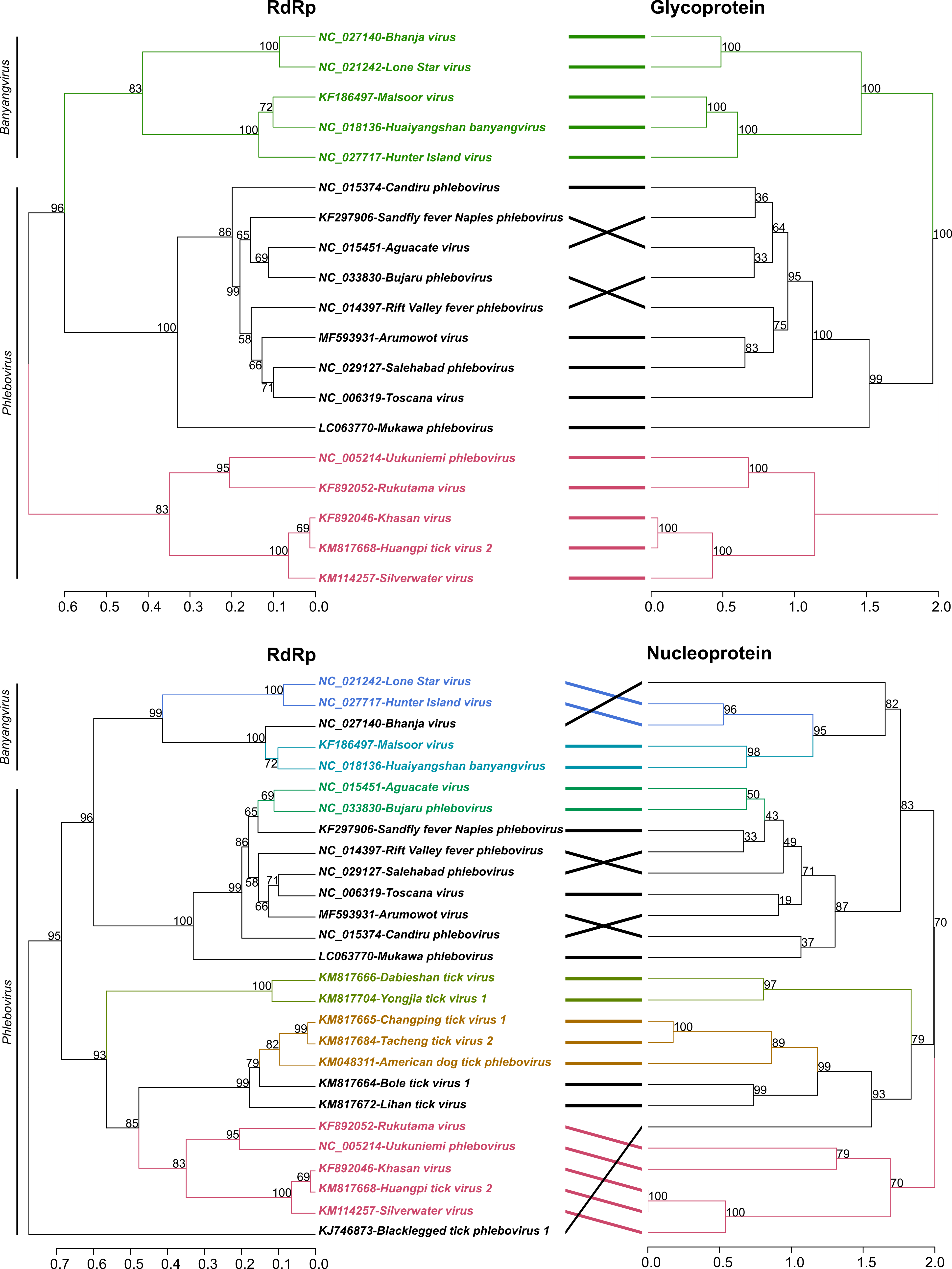

Supplement: S29 Fig — Topologically congruent clades are highlighted in color. Branches in black indicate taxa that do not share a common topological pattern in the respective tree pairs. Banyangvirus is sister to the main Phlebovirus clades in the RdRp phylogeny, indicating that Banyangvirus should not be regarded as an independent genus. (TIF) [file ppat.1008224.s034.tif]

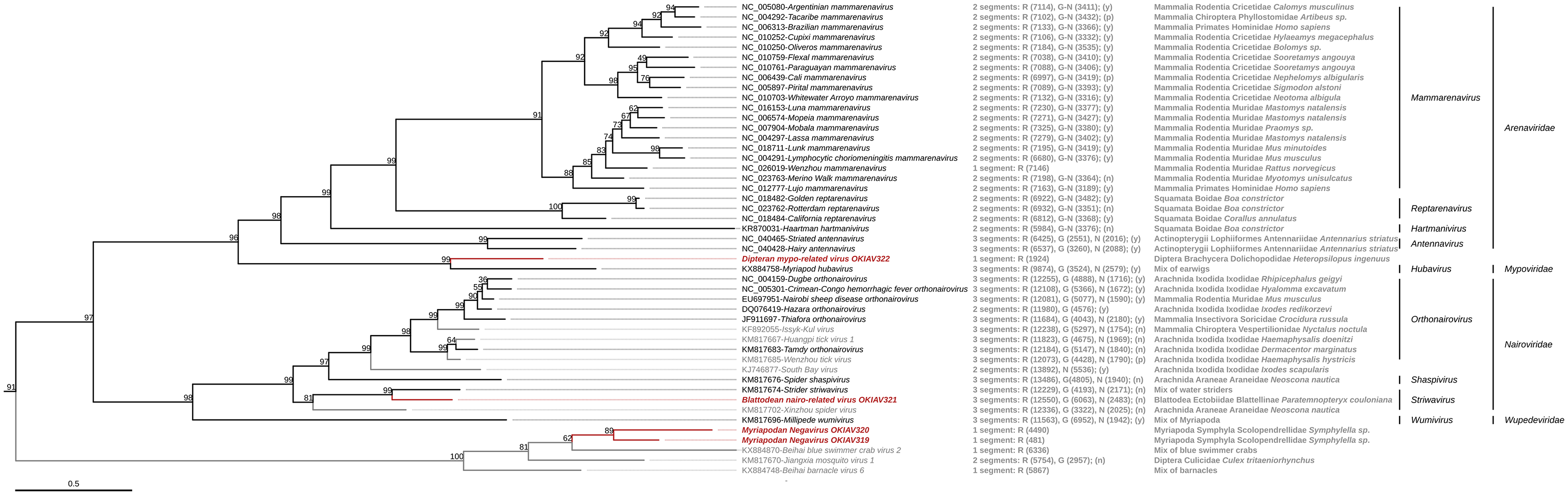

Supplement: S30 Fig — Maximum likelihood phylogenies based on RAxML. Black branches show ICTV-accepted taxa, grey branches show unclassified taxa, and red branches show OKIAVs. Columns on the right summarize contig length, genome completeness, number of segments, taxonomic grouping of hosts, and viral genus and family. Genomic protein-coding regions are: R = RdRp, G = glycoprotein, N = nucleoprotein, Hy = hypothetical protein with unknown function. Segment lengths are shown in parentheses. Information on the segment ends is indicated by: (n) = segment ends not matching the ends of the RdRp segment, (y) = segment ends matching the ends of the RdRp segment, (p) = segment ends partially matching the ends of the RdRp segment. The outgroup taxon (not shown) is Rift Valley fever phlebovirus (Phenuiviridae). Analyses based on PhyML and MrBayes can be found in S31 and S32 Figs. (TIF) [file ppat.1008224.s035.tif]

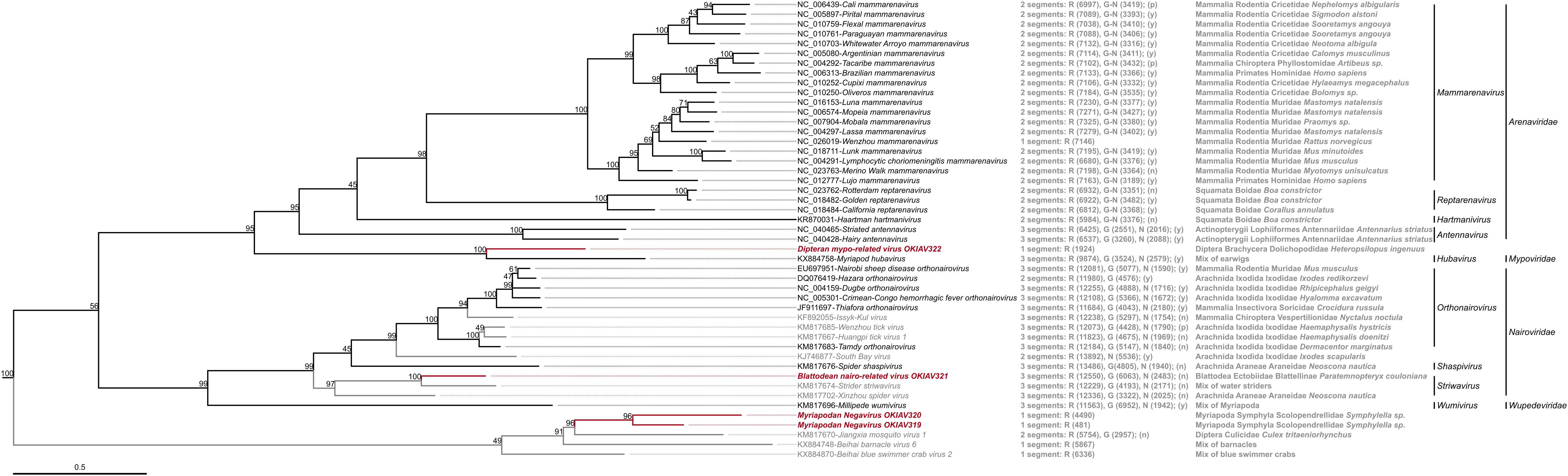

Supplement: S31 Fig — Black branches show ICTV-accepted taxa, grey branches show unclassified taxa, and red branches show OKIAVs. Columns on the right side summarize contig length, genome completeness, number of segments, taxonomic grouping of hosts, and viral genus and family. Genomic protein-coding regions are: R = RdRp, G = glycoprotein, N = nucleoprotein, Hy = hypothetical protein with unknown function. Segment lengths are shown in parentheses. Information on sequence similarity of the segment ends among different genome segments is given as well: (n) = segment ends not matching the ends of the RdRp segment, (y) = segment ends matching the ends of the RdRp segment, (p) = segment ends partially matching the ends of the RdRp segment. The outgroup taxon (not shown) is Rift Valley fever phlebovirus (Phenuiviridae). (TIF) [file ppat.1008224.s036.tif]

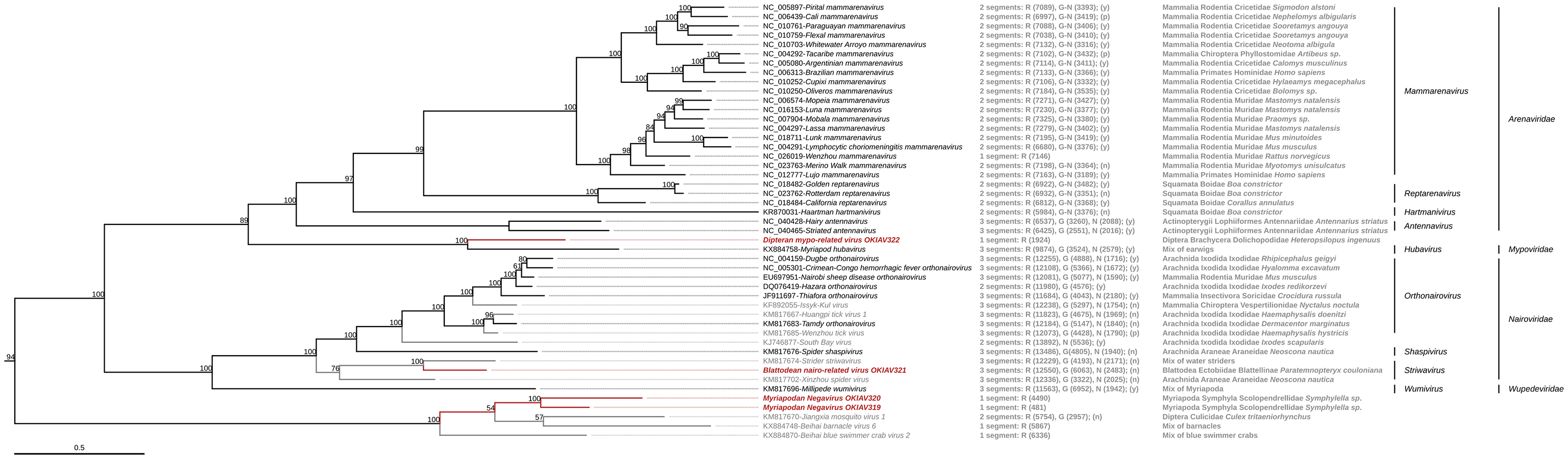

Supplement: S32 Fig — Black branches show ICTV-accepted taxa, grey branches show unclassified taxa, and red branches show OKIAVs. Columns on the right side summarize contig length, genome completeness, number of segments, taxonomic grouping of hosts, and viral genus and family. Genomic protein-coding regions are: R = RdRp, G = glycoprotein, N = nucleoprotein, Hy = hypothetical protein with unknown function. Segment lengths are shown in parentheses. Information on sequence similarity of the segment ends among different genome segments is given as well: (n) = segment ends not matching the ends of the RdRp segment, (y) = segment ends matching the ends of the RdRp segment, (p) = segment ends partially matching the ends of the RdRp segment. The outgroup taxon (not shown) is Rift Valley fever phlebovirus (Phenuiviridae). (TIF) [file ppat.1008224.s037.tif]

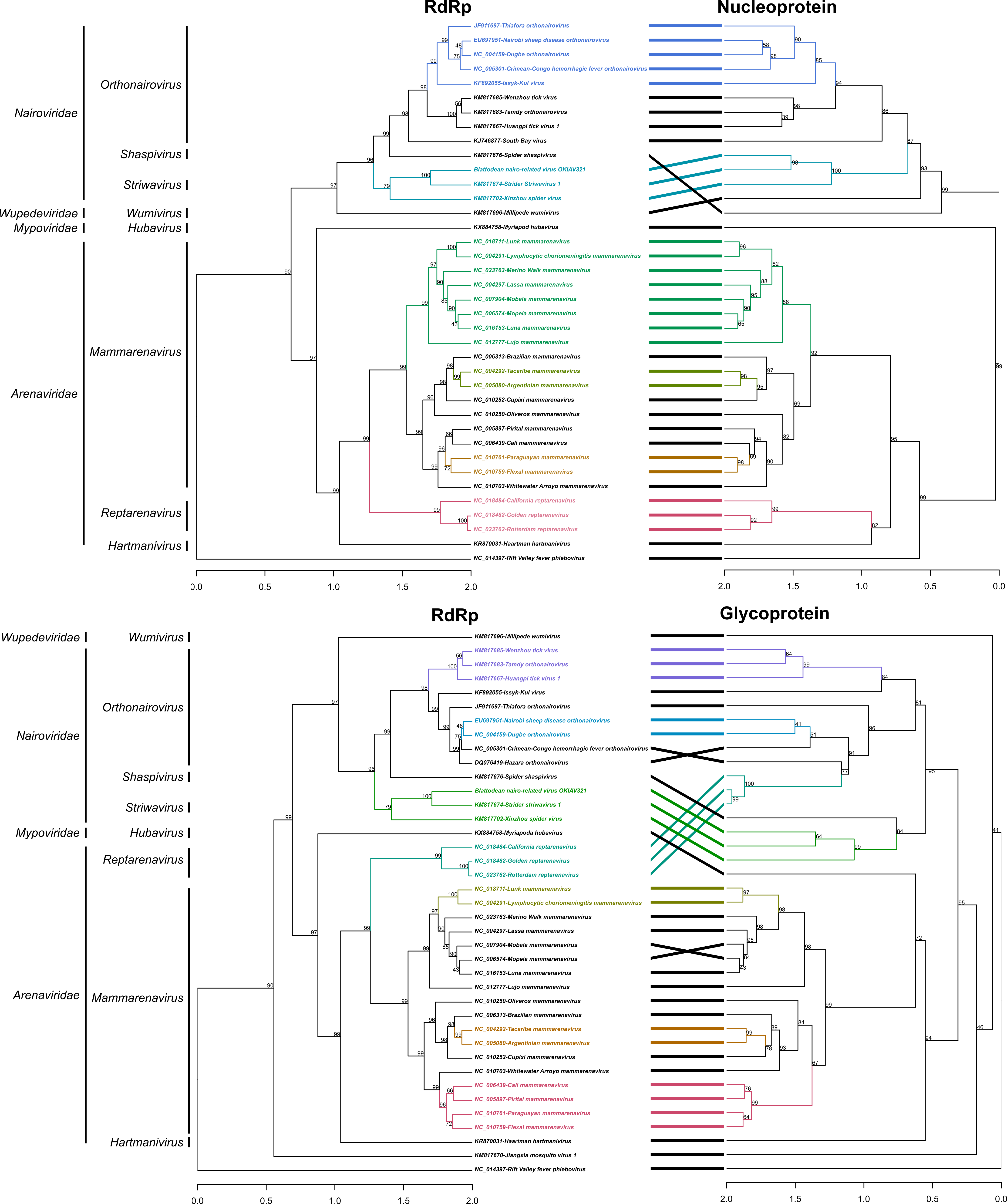

Supplement: S33 Fig — Topologically congruent clades are highlighted in color. Branches in black indicate taxa that do not share a common topological pattern in the respective tree pairs. In the phylogenies of RdRp and nucleoprotein, 31 of 38 taxa are distributed in the monophyletic genera Orthonairovirus, Reptarenavirus, and Mammarenavirus. Reptarenavirus is the only genus that consistently has a congruent topology among all trees. There are very few viruses that are not formally accepted by ICTV in this tree, and the ones that are new fit well in between the established genera, resulting in a stable backbone of the phylogeny. In the phylogenies of RdRp and glycoprotein, 30 of 39 taxa are distributed in Orthonairovirus, Reptarenavirus, and Mammarenavirus. The glycoprotein phylogeny shows similar topology to the nucleoprotein one, but only Reptarenavirus maintains its topological position. The biggest disagreement is the positioning of Reptarenavirus within the Nairoviridae clade. The backbones of the trees are not in agreement, therefore the positions of the single-species genera Shaspivirus, Wumivirus, and Hubevirus cannot be confirmed. However, in both phylogenies, the positions of Striwavirus, Blattodean nairo-related virus OKIAV321, and Xinzhou spider virus are stable. (TIF) [file ppat.1008224.s038.tif]

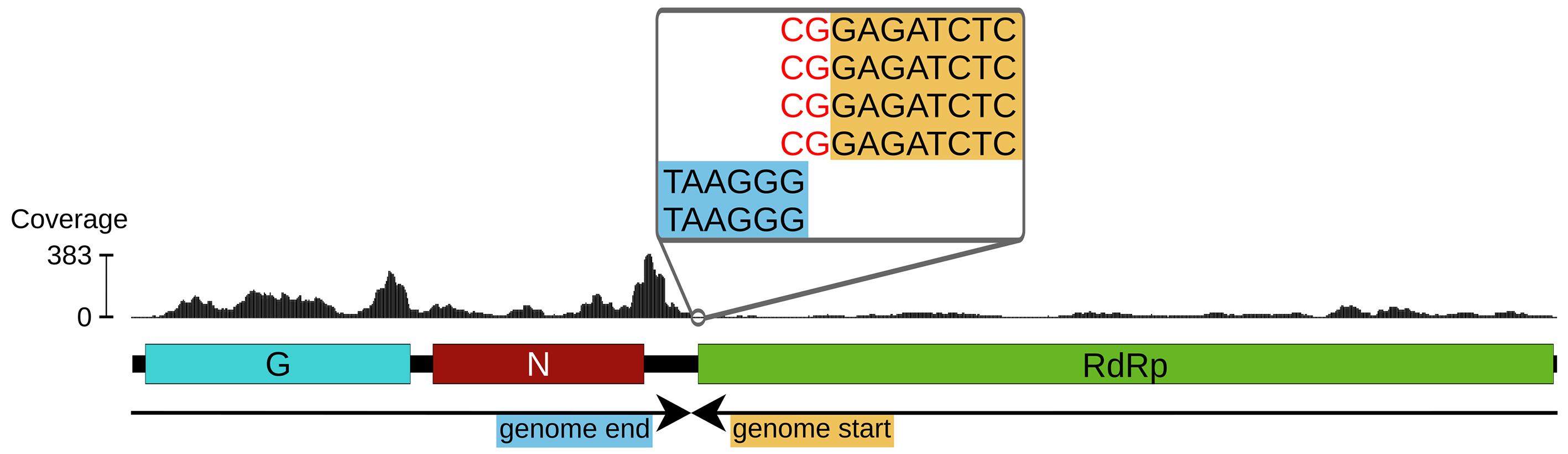

Supplement: S34 Fig — The sequence is joined head-to-tail, genome start and end are colored and indicated by arrows. The end-to-start gap is solely bridged by two flanking nucleotides of four reads (marked in red). The ORFs encoding for glycoprotein (G), nucleoprotein (N), and RdRp, as well as the read coverage are shown. (TIF) [file ppat.1008224.s039.tif]
